# Supplementary material for: Patient and healthcare provider knowledge, attitudes and barriers to handover and healthcare communication during chronic disease inpatient care in India: a qualitative exploratory study
Source: BMJ Open. 2019 Nov 11;9(11):e028199. doi: 10.1136/bmjopen-2018-028199 (PMC6858202; doi:10.1136/bmjopen-2018-028199)
Supplement: Supplementary data [file bmjopen-2018-028199supp001.pdf]

S1. Additional information regarding the Indian healthcare system

1. National context: structure of public healthcare system

The basic structure of the public healthcare system in India is as follows:[1]

- National level: Ministry of Health and Family Welfare.
- State-level: State Department of Health and Family welfare in each state.
- Regional level: covers 3 - 5 districts. Headed by State Directorate of Health.
- District level: Middle-level management organisation serving as a link between the regional and state structures and the peripheral and PHC structures.
- Sub-divisional/Taluk level: Hospitals/hospitals with specialty care (Taluk headquarters hospitals). Healthcare services are rendered via the office of Assistant District Health and Family Welfare Officer.
- Community level: CHCs that cater for 80,000-120,000 population and PHCs that cover approximately 20,000 – 30,000 population.

2. Public healthcare in India

The quality of public healthcare across India varies notably between states and urban and rural areas. Whilst there are some examples of excellence in a select few states, generally the public sector is generally failing to meet the basic healthcare needs of the growing population. Services being too far away, a lack of trained personnel, deficient medical supplies and limited facility opening times are some of the principal reasons for current shortfalls.[2] Regarding primary health

centres, government estimates have indicated that up to 10% are without a doctor, 37% are without a laboratory technician and 25% are without a pharmacist.[3] These issues appear to be particularly prevalent within socioeconomically vulnerable areas, where facilities have been reported to lack essential medical supplies and are closed more than 50% of the time. In addition, public healthcare facilities are often the sole source of qualified healthcare professionals in rural areas, which is where many of the most socioeconomically vulnerable live.[2]

3. Private healthcare in India

Due to a lack of prioritisation from economic planners, public healthcare in India has lacked funding over multiple decades. Therefore, the increasing prevalence of chronic diseases, as well as ongoing challenges of infectious diseases, has placed an enormous strain on public health systems that cannot be feasibly managed. Subsequently, the private healthcare sector has proliferated to meet the rising needs, expectations and earnings of the growing population. So much so in fact, that surveys now show that private providers dominate healthcare service provision in India.[2]

At the top end of the market, the private healthcare sector has grown extensively and now has world-class facilities. Private hospital care has become an export sector for medical tourism that cares for around 200,000 international patients each year.[4] However, private healthcare providers lack regulation and quality remains inconsistent across facilities. In many cases private facilities deliver services without appropriate equipment or expertise and, although it is preferential for many to visit private providers, high out-of-pocket costs are often incurred. It has been reported that more than 40% of private hospital patients have to sell assets or

borrow money to fund their care.[5] This means that many patients of lower socioeconomic status remain unable to access such care, while others fall further into poverty as a result of expenses.

REFERENCES

1. Care Continuum Alliance, India. India – Health Care System Structure and Organization. 2009. Available from: http://dmaa.pbworks.com/w/page/17960772/India%20-%20Health%20Care%20System%20Structure%20and%20Organization

2. Joumard I, Kumar A. Improving health outcomes and health care in India. Paris: Organisation for Economic Cooperation and Development (OECD) Working Papers, No. 1184, OECD Publishing. 2015. Available from: http://www.oecd-ilibrary.org/economics/improving-health-outcomes-and-health-care-in-india_5js7t9ptcr26-en

3. Ministry of Health and Family Welfare, Government of India. Rural Health Statistics in India. 2012. Available from: https://nrhm.gujarat.gov.in/images/pdf/publications/RHS_2012.pdf

4. Lunt N, Smith R, Exworthy M, Green S, Horsfall D, Mannion R. Medical Tourism: Treatments, Markets and Health System Implications: a Scoping Review. OECD Publishing. 2010. Available from: Paris http://www.oecd.org/els/health-systems/48723982.pdf

5. The World Bank - Health, Nutrition, Population Sector Unit India South Asian Region. Raising the sights: better health systems for India's poor. Available from: https://openknowledge.worldbank.org/handle/10986/14080

S2. Additional information regarding the study settings

1. Himachal Pradesh

Himachal Pradesh is principally a rural state in northern India. It has a population of 6.86 million people and the average literacy rate is 83.3%, which is higher than the national average (74%). However, rates remain notably lower for women compared to men (76.6% vs. 90.8%, respectively).[6] Private HCPs are less prevalent in Himachal Pradesh compared to many states and utilisation of public healthcare remains relatively high.[7] A recent study found that the availability of public health services in the state was deemed adequate as compared to standards of other hill states, but with an unequal distribution of resources across regions.[8]

2. Kerala

Kerala state is in the south-west of India. It has a population of 34.8 million people and a greater than national average urban-based population of 47.7%. It has the highest overall literacy rate of all the states in India (93.9%; men 96.1%, women 92.1).[9] There are a relatively large number of government healthcare facilities in Kerala but the healthcare environment has become increasingly complex due to the growing popularity and presence of private healthcare facilities, which are predominantly situated in urban settings. Despite this, public healthcare facilities in Kerala remain the first point of care for many patients and continue to deliver essential services.[10]

REFERENCES

6. Government of India. Himachal Pradesh Population Census Data 2011 [Internet]. Available from: https://www.census2011.co.in/census/state/himachal+pradesh.html accessed 13/06/18 2017.

7. Government of India. Himachal Pradesh Development Report. New Delhi: Academic Foundation, 2005.

8. Suman.; Bhutani S. Availability of health services in Himachal Pradesh *International Journal of Advanced Research and Development* 2017;2(6):231-38.

9. Government of Kerala. Kerala Population Census Data 2011 [Internet]. Available from: https://www.census2011.co.in/census/state/kerala.html accessed 13/06/18.

10. Government of Kerala. Kerala Development Report. New Delhi: Academic Foundation, 2008.
