# Supplementary material for: Patient and healthcare provider knowledge, attitudes and barriers to handover and healthcare communication during chronic disease inpatient care in India: a qualitative exploratory study
Source: BMJ Open. 2019 Nov 11;9(11):e028199. doi: 10.1136/bmjopen-2018-028199 (PMC6858202; doi:10.1136/bmjopen-2018-028199)
Supplement: Author's manuscript [file bmjopen-2018-028199.draft_revisions.pdf]

BMJ Open is committed to open peer review. As part of this commitment we make the peer review history of every article we publish publicly available.

When an article is published we post the peer reviewers' comments and the authors' responses online. We also post the versions of the paper that were used during peer review. These are the versions that the peer review comments apply to.

The versions of the paper that follow are the versions that were submitted during the peer review process. They are not the versions of record or the final published versions. They should not be cited or distributed as the published version of this manuscript.

BMJ Open is an open access journal and the full, final, typeset and author-corrected version of record of the manuscript is available on our site with no access controls, subscription charges or pay-per-view fees (http://bmjopen.bmj.com).

If you have any questions on BMJ Open's open peer review process please email info.bmjopen@bmj.com

BMJ Open

Investigating inpatient and healthcare provider knowledge, attitudes and barriers to handover communication in India: A qualitative study

Journal:	*BMJ Open*
Manuscript ID	bmjopen-2018-028199
Article Type:	Research
Date Submitted by the Author:	26-Nov-2018

Complete List of Authors:	Humphries, Claire; University of Birmingham, UK, Institute of Applied Health Research<br>Jaganathan, Suganthi ; Centre for Chronic Disease Control<br>Panniyammakal, Jeemon ; Centre for Chronic Disease Control<br>Singh, Sanjeev; Amrita Institute of Medical Sciences and Research Centre; Amrita Institute of Medical Sciences and Research Centre<br>Goenka, Shifalika; Public Health Foundation of India<br>Dorairaj, Prabhakaran ; Centre for Chronic Disease Control<br>Gill, Paramjit ; University of Warwick<br>Greenfield, Sheila; University of Birmingham, Primary Care Clinical Sciences<br>Lilford, Richard; University of Warwick, Division of Health and Population Sciences<br>Manaseki-Holland, Semira; University of Birmingham,

Keywords:	International health services < HEALTH SERVICES ADMINISTRATION & MANAGEMENT, Quality in health care < HEALTH SERVICES ADMINISTRATION & MANAGEMENT, PUBLIC HEALTH, QUALITATIVE RESEARCH

SCHOLARONE™
Manuscripts

Investigating inpatient and healthcare provider knowledge, attitudes and barriers to handover communication in India: A qualitative study

Claire Humphries[1¶], Suganthi Jaganathan[2,3¶], Jeemon Panniyammakal[2,3,4], Sanjeev Singh[5], Shifalika Goenka[2,3], Prabhakaran Dorairaj[2,3], Paramjit Gill[5], Sheila Greenfield[1], Richard Lilford[6], Semira Manaseki-Holland[1*]

[1] Institute of Applied Health Research, University of Birmingham, Birmingham, West Midlands, United Kingdom

[2] Public Health Foundation of India, New Delhi, Delhi, India

[3] Centre for Chronic Disease Control, New Delhi, Delhi, India

[4] Sree Chitra Tirunal Institute of Medical Sciences and Technology, Trivandrum, Kerala, India

[5] Hospital Administration, Amrita Institute of Medical Sciences, Kochi, Kerala, India

[6] Centre for Applied Health Research and Delivery, University of Warwick, Warwick, Warwickshire, United Kingdom

*Corresponding author

Email: S.ManasekiHolland@bham.ac.uk

¶ These authors contributed equally to this work.

Manuscript Word Count: 4993

For peer review only - http://bmjopen.bmj.com/site/about/guidelines.xhtml

ABSTRACT

Objectives 1. To investigate patient and healthcare provider knowledge (HCP), attitudes and barriers to handover communication during inpatient care. 2. To explore potential interventions for improving the storage and transfer of critical healthcare information.

Methods *Design:* Qualitative study comprising 41 semi-structured, individual interviews. Thematic analysis using the Framework Method with analyst triangulation. *Setting*: Three hospitals in Himachal Pradesh and Kerala, India. *Participants:* 20 male (n=10) and female (n=10) chronic NCD patients and 21 male (n=15) and female (n=6) HPCs. Purposive sampling was used to identify patients with chronic NCDs (Chronic Respiratory Disease, Cardiovascular Disease, Diabetes or Hypertension) and HCPs working in the study hospitals.

Results For chronic NCD patients, three themes emerged: (1) *Public healthcare service characteristics;* (2) *HCP-patient communication;* (3) *Attitudes regarding medical information.* For HCPs, three themes emerged: (1) *System factors;* (2) *Information exchange practices;* (3) *Quality improvement strategies.* Whilst some content within themes was unique to each participant group, there was substantial overlap. Both patients and HCPs recognised constraints affecting public healthcare; deficient primary care services placed increased pressure on hospitals, subsequently limiting HCP consultation times. HCP and IP reports also indicated an absence of structured referral formats, resulting in fragmented information transfer. Additionally, whilst patient-held documents were a key vehicle for information exchange between HCPs, not all patients transported them and HCPs stated that this hindered continuity of care. Inpatient descriptions of HCP communication indicated notable inconsistencies and a lack of patient-centeredness. HCPs reported systemic issues such as absence of formal handover communication systems and training.

For peer review only - http://bmjopen.bmj.com/site/about/guidelines.xhtml

Conclusions Handover communication for chronic NCD patients visiting public hospitals in India is currently suboptimal. Structured information exchange systems are urgently required to improve quality, continuity and safety of care. Our findings indicate that well-designed patient-held record booklets may be an acceptable and effective part of the solution.

STRENGTHS AND LIMITATIONS OF THIS STUDY

- This is the first qualitative study, as far as the authors are aware, to investigate handover communication within and between levels of healthcare in India.
- The number of interviews conducted with both patients and healthcare providers ensured data saturation and provided a variety of critical perspectives.
- Analyst triangulation corroborated data analysis and strengthened the credibility of the study.
- Recruitment challenges meant that inpatients were predominantly older (i.e. 45yrs+), therefore experiences of younger patients could not be thoroughly explored.
- Awareness of the interviewer's context as a public health researcher may have resulted in participants distorting their responses to minimise critical judgement.

For peer review only - http://bmjopen.bmj.com/site/about/guidelines.xhtml

1. INTRODUCTION

The increasing burden of chronic, non-communicable diseases (NCDs) such as cardiovascular disease, diabetes and chronic respiratory disease has become a global pandemic that is disproportionately affecting low and middle-income countries (LMICs).[1] This is placing great demand on under-resourced health systems that can only be relieved by employing efficient and integrated approaches to healthcare management. Central to efficiency and integration in healthcare is effective handover communication, which involves the exchange of patient-specific information between healthcare providers (HCPs) and between HCPs and patients/carers.[2]

Between HCPs, information exchange is critical during clinical handovers, which are the points in care where information, responsibility and accountability for patient care are transferred from one HCP to another.[3] Such exchange is vital because safe and effective treatment can only be maintained if all relevant information has been shared and understood.[4] A wealth of research from high-income countries (HICs) has evidenced the association between communicative breakdowns during care transitions and significant risks to patient safety. These risks include delays in diagnosis, medication errors and life-threatening adverse events.[5 6] Additionally, effective information exchange between HCPs and patients is vital, as patients can provide valuable information to HCPs involved at various stages of their care pathway.[4] Excellent HCP-patient communication also empowers patients to become active participants in their healthcare management; this is a key aspect of patient-centred care, which has been linked to improved patient satisfaction and outcomes.[7]

Despite the established importance of handover communication for health systems functioning and patient safety, there is currently a dearth of LMIC-based research on this topic. Only a few studies from India have evaluated and described deficiencies in information

For peer review only - http://bmjopen.bmj.com/site/about/guidelines.xhtml

exchange during hospital shift-change and discharge.[8-10] The authors of this study have completed a mixed-methods development project focussing on handover and continuity of care for chronic NCD patients in Kerala and Himachal Pradesh states, India. As we explored the quality of handover communication for outpatients, we found that whilst all patients received documented information during consultations, the contents of this varied substantially. Critically, many notes did not contain all items of information necessary for facilitating continuity of care.[11] In addition, our quantitative study of handover during hospital discharge has found significant associations between failure to receive key healthcare information on discharge notes and an increased likelihood of adverse health outcomes (Humphries, Jaganathan, Panniyammakal, *et al.* 2018).

The current study was conducted to gain further insight into the transfer of healthcare information for chronic NCD inpatients in the same study areas of India. The primary objective was to explore knowledge, attitudes and barriers to handover communication during the following points of care:

- Referral/transfer (i.e. communication between HCPs and between HCPs and patients when referring and/or transferring patients)
- Hospital admission and discharge (i.e. communication between HCPs and patients regarding condition, treatment and/or management during admission and discharge)

A secondary objective was to explore possible interventions to improve the storage and transfer of critical healthcare information.

For peer review only - http://bmjopen.bmj.com/site/about/guidelines.xhtml

2. METHODS

2.1 Overview

We report findings from a qualitative study of handover communication for chronic NCD inpatients in two Indian states. This study was conducted from December 2014 to November 2015 across three hospitals: one rural secondary-care hospital in Himachal Pradesh state, and one peri-urban secondary-care and one urban tertiary-care hospital in Kerala state. These settings were selected to capture a range of hospital types within different geographical settings. We selected public rather than private facilities as this is where a large proportion of vulnerable patients access healthcare. See supplementary files "S1" and "S2" for further information regarding the Indian healthcare system and study settings.

2.2 Participant recruitment

2.2.1 Inpatients

Inpatients were recruited opportunistically from hospitals by trained research assistants (n=6).[12] Purposive sampling was used to identify individuals who met the following inclusion criteria: adults (18 years+),[13] admitted to hospital within 24 hours of a researcher first meeting them, due to complications from one of the following chronic NCDs: cardiovascular disease, chronic respiratory disease, diabetes mellitus, or hypertension. The identification process took place via researchers approaching ward nurses and asking them about patient demographic and admission details; patients were excluded if judged too unwell to participate by ward nurses. Patients who met the inclusion criteria were provided with verbal and documented study information. Written consent was obtained from literate patients. For illiterate patients, oral consent was obtained along with a thumbprint and

For peer review only - http://bmjopen.bmj.com/site/about/guidelines.xhtml

signature from a literate witness (i.e. family member/carer) in line with World Health Organisation guidelines.[14] Inpatients were recruited until theoretical saturation was achieved;[15] A total of 20 inpatients participated.

2.2.2 Healthcare Professionals

Healthcare professionals (HCPs) were recruited from study hospitals by trained research assistants (n=6). Due to the busy nature of the study settings, opportunistic sampling was used to recruit as many HCPs as possible with a range of roles and experience.[12] If HCPs stated that they were too busy to answer questions they were deemed "unavailable" and not included in the study. HCPs were also recruited until theoretical saturation was achieved;[15] A total of 21 HCPs participated.

2.3 Sample Size

As well as saturation being reached for both participant groups independently, the resulting sample size of 41 participants for this study was in accordance with Baker and Edwards's review of sample sizes utilised in qualitative literature, indicating it was sufficient for achieving overall data saturation.[16]

2.4 Data collection

Interview data was collected entirely by the lead Indian researcher (SJ – an experienced public health researcher), who was not local to the study areas. Full consideration was given prior to and throughout data collection to ensure that SJ was aware

For peer review only

of the potential limitations of working with participants from culturally and linguistically diverse backgrounds. SJ was not involved in patient treatment or previously known to HCPs. Inpatient interviews took place either on hospital wards or at patients' homes (depending on convenience); interviews at patients' homes took place five weeks after discharge. HCP interviews took place in hospital offices. All interviews were conducted in either Hindi, English, Malayalam or a mixture depending on interviewee preference and audio recorded using a digital Dictaphone.

Data collection took place in two stages. In the first stage (December 2014–October 2015), pre-prepared topic guides were used to guide interviews (see supplementary file "S3"). Both inpatient and HCP guides included open-ended questions focussing on experiences and attitudes of healthcare visits and information exchange. The HCP topic guide differed slightly to capture information on health systems policy and practice; it also included questions regarding handover training and potential interventions for improving practices. Following this stage, on the 11th of October 2015, a handover expert's meeting took place in India to present preliminary findings and discuss possible interventions.

Researchers from the University of Birmingham and University of Warwick (UK) facilitated the presentation of results and group discussions at the meeting. Representatives (n=27) from the following international, Indian national and state-level organisations participated: The World Health Organisation; The World Bank; ACCESS Health International; The Ministry of Health and Family Welfare; The Public Health Foundation of India; The National Centre for Disease Control; The Centre for Chronic Disease Control; The National Health System Resource Centre; The All India Institute of Medical Sciences; Aga Khan Health Services; AMRITA Institute of Medical Sciences and Fortis Hospitals. During discussions, a consensus was reached that patient-held record booklets were likely to be an acceptable and sustainable intervention to improve information exchange. This was based on

For peer review only - http://bmjopen.bmj.com/site/about/guidelines.xhtml

the international success of similar patient-held records used in maternal healthcare around the world.[17-21] It also took into account the delays in developing universal electronic information systems. Overall, it was opted as the most pragmatic intervention and numerous experts felt that booklets could also improve patient self-management if they contained disease-specific advice.

Therefore, following the meeting the second stage of qualitative data collection (October–November 2015) commenced. Topic guides were updated to include questions regarding the utility of patient-held booklets (see supplementary file "S4"). In addition, if participants stated they had limited time then researchers interviewed them using a shortened topic guide containing targeted questions on patient-held booklets (see supplementary file "S4").

2.5 Data Analysis

All audio recordings of interviews were transcribed verbatim and, if necessary, translated into English by SJ. All transcripts were then sent to the lead UK researcher (CH – public health PhD student) for analysis. Data was analysed using the Framework Method,[22] as this is the method most commonly used for semi-structured interview transcripts. Analysis occurred through the following stages central to the Framework Method: transcription, familiarisation, coding, charting, and interpretation. Over a one-month period, familiarisation with the data took place via slow reading of transcripts and CH consulted with SJ to gain a clear understanding of interview contexts. Once this was complete, coding began and two transcripts were chosen at random from each batch of interviews (i.e. 2 inpatients and 2 HPC transcripts) for independent coding by an additional analyst (SG – professor of medical sociology) for analyst triangulation.[23] Inpatient and HCP transcripts were coded separately in

For peer review only - http://bmjopen.bmj.com/site/about/guidelines.xhtml

order to be able to assess similarities and differences between participant groups - Inpatient transcripts were coded first. The coding process involved further familiarisation with the data, followed by open coding where certain transcript content was highlighted and allocated descriptive labels (codes) to interpret the phenomena identified in the text. The development of codes and themes was entirely data-led and analysed manually.[24]

Microsoft Excel was used to organise participant codes. CH created initial categories by clustering similar codes developed from the two randomly selected inpatient and HCP transcripts. CH and the additional analyst (SG) then met to discuss their analyses. As both had produced similar codes and concepts, the categories that were created were mutually agreed upon. CH then continued with category development until all transcripts had been coded and inserted onto the spreadsheet. Following analysis of 20 inpatient and 26 HCP transcripts, no new categories had been produced. This served as confirmation that data saturation had been met.[15]

Following coding, categories were grouped into subcategories and linked to produce themes. Then, via the process of charting,[22 24] themes for each participant group were used to create a framework matrix into which participants' quotes were inserted, corresponding to their representative subcategory. This provided a visual representation of themes, which facilitated the mapping and interpretation of the data. After completing separate analysis of patient and HCP data, results of both participant groups were compared to assess similarities and differences between their reports of knowledge, attitudes and barriers to handover communication. A Venn diagram was used to summarise the separate and overlapping content, which was linked to sub-categories from original themes.

For peer review

For peer review only - http://bmjopen.bmj.com/site/about/guidelines.xhtml

3. RESULTS

3.1 Inpatient characteristics

20 male (n=10) and female (n=10) inpatients aged between 25 and 71 were interviewed. Participants' background characteristics were varied (Table 1). Inpatients completed interviews in English (n=11), Hindi (n=4), Malayalam (n=4) and a mixture of Hindi and English (n=1).

For peer review only

Table 1. Inpatient characteristics

Characteristics	1	2	3	4	5	6	7	8	9	10	11	12	13	14	15	16	17	18	19	20	N (%)
**Age**	65	45	70	58	71	56	57	70	55	25	72	50	55	69	70	50	70	70	70	70	25-71
**Sex**																					
Male	✓	✓	✓	✓	✓				✓	✓	✓	✓				✓					10 (50)
Female						✓	✓	✓					✓	✓	✓		✓	✓	✓	✓	10 (50)
**Literacy**																					
Illiterate								✓					✓		✓	✓	✓	✓	✓	✓	8 (40)
Literate	✓	✓	✓	✓	✓	✓	✓		✓	✓	✓	✓		✓							12 (60)
**Education Level**																					
None/minimal primary school-level								✓				✓	✓		✓	✓	✓	✓	✓	✓	9 (45)
Completed lower primary school					✓		✓														2 (10)
Completed upper primary school						✓															1 (5)
Completed secondary school									✓												1 (5)
Completed higher vocational studies																					0 (0)
University graduate (or above)	✓			✓						✓	✓										4 (20)
No data		✓	✓											✓							3 (15)
**Employment Status**																					
Employed		✓		✓	✓				✓			✓	✓	✓		✓					8 (40)
Unemployed						✓	✓	✓							✓		✓	✓	✓	✓	8 (40)
Student										✓											1 (5)
Retired	✓		✓								✓										3 (15)
**Chronic NCD(s) (related to admission)**																					
Chronic Respiratory Disease			✓	✓		✓		✓					✓			✓					6 (30)
Diabetes	✓													✓	✓	✓	✓	✓	✓	✓	8 (40)
Hypertension									✓		✓	✓				✓	✓	✓			6 (30)
Cardiovascular Disease (other than Hypertension alone)	✓	✓			✓		✓	✓	✓	✓	✓	✓									9 (45)
**Language(s) used during Interview**																					
English (only)	✓	✓	✓	✓										✓	✓	✓	✓	✓	✓	✓	11 (55)
Hindi (only)						✓	✓	✓	✓												4 (20)
Malayalam (only)										✓	✓	✓	✓								4 (20)
English & Hindi (mixture)					✓																1 (5)

For peer review only - http://bmjopen.bmj.com/site/about/guidelines.xhtml

3.2 Healthcare Professional characteristics

21 male (n=15) and female (n=6) HCPs aged between 22 and 55 were interviewed. HCP roles included doctors (n=17), nurses (n=2), pharmacists (n=1) and medical records officers (n=1). HCP qualifications and experience were varied (Table 2). HCPs completed interviews in English (n=15), Hindi (n=2), Malayalam (n=2) and a mixture of Hindi and English (n=2).

For peer review only

For peer review only - http://bmjopen.bmj.com/site/about/guidelines.xhtml

Table 2. Healthcare professional characteristics

Characteristics	1	2	3	4	5	6	7	8	9	11	14	15	18	19	20	21	22	23	24	25	26	N (%)
**Age**	44	24	33	25	23	39	44	35	52	50	50	43	50	40	46	55	22	35	35	45	35	22-55
**Sex**																						
Male	√		√	√	√		√		√	√	√	√	√	√	√	√	√			√		15 (71.4)
Female		√				√		√										√	√		√	6 (28.6)
**Qualification/s***																						
MD	√		√				√	√	√	√	√	√	√	√	√	√	√					13 (61.9)
MPH						√																1 (4.8)
MBBS	√	√	√	√	√	√	√	√	√	√	√	√	√	√	√	√	√					17 (81.0)
BSc Nursing																		√			√	2 (9.5)
BS Pharmacy																			√			1 (4.8)
Graduate (non-medical degree)																				√		1 (4.8)
**Official position**																						
Medical Superintendent						√																1 (4.8)
Chief Medical Officer									√													1 (4.8)
Medical Officer												√										1 (4.8)
Consultant	√		√				√	√					√	√	√	√	√					9 (42.9)
Surgeon		√								√												2 (9.5)
General Medicine											√											1 (4.8)
Intern Doctor				√	√																	2 (9.5)
Ward Nurse																		√			√	2 (9.5)
Pharmacist																			√			1 (4.8)
Medical Records Officer																				√		1 (4.8)
**Years of experience in position**																						
<1		√			√																	2 (9.5)
1 – 3				√																		1 (4.8)
4 – 6						√		√										√		√	√	5 (23.8)
7 – 10			√				√												√			3 (14.3)
>10	√								√	√	√	√	√	√	√	√	√					10 (47.6)
**Place of work**																						
General Hospital		√	√	√	√									√		√	√	√	√			9 (42.9)
Regional Hospital	√								√	√	√		√							√	√	7 (33.3)
Taluk Hospital						√	√	√				√			√							5 (23.8)
**Language(s) used during interview**																						
English (only)		√	√	√	√	√	√	√	√	√	√	√		√	√	√	√					15 (71.4)
Hindi (only)																				√	√	2 (9.5)
Malayalam (only)																		√	√			2 (9.5)
English & Hindi (mixture)	√												√									2 (9.5)

For peer review only

3.3 Charted Data

During analysis of patient and HCP data, three themes (with subcategories) emerged for each participant group. Patient themes were 1.Public healthcare service characteristics, 2. HCP to patient communication and 3.Attitudes regarding medical information (Table 3). HCP themes were 1.System factors, 2.Information exchange practices and 3.Quality improvement strategies (Table 4).

Following separate analysis of patient and HCP data, the results of both participant groups were compared to assess similarities and differences between their reports of knowledge, attitudes and barriers to handover communication. The results of this comparison are displayed in Figure 1 (see supplementary file "S5").The similarities will be described first, followed by the differences. To ensure confidentiality, numerical pseudonyms have been used when presenting quotes.

For peer review only

Table 3. Summary of charted data for inpatients (IPs)

	Public healthcare service characteristics		HCP to patient communication			Attitudes regarding medical information		
**IP**	**Large patient loads (overcrowding)**	**Deficient primary care services**	**Verbal healthcare information (during admission)**	**Referral information**	**Impoliteness/ impatience**	**Transportation of medical documents**	**Patient-held booklet intervention**	**Dissatisfaction with lifestyle advice**
**1**						✓		
**2**			✓					
**3**	✓		✓		✓	✓		
**4**			✓			✓		
**5**		✓	✓	✓	✓			
**6**				✓		✓		
**7**	✓	✓		✓		✓		
**8**				✓				
**9**			✓					
**10**			✓					
**11**	✓		✓			✓		
**12**			✓	✓		✓		
**13**			✓			✓		
**14**		✓	✓				✓	✓
**15**		✓	✓		✓		✓	✓
**16**		✓	✓			✓	✓	
**17**			✓			✓	✓	✓
**18**		✓	✓			✓	✓	
**19**			✓			✓	✓	
**20**			✓				✓	✓

For peer review only

For peer review only - http://bmjopen.bmj.com/site/about/guidelines.xhtml

Table 4. Summary of charted data for healthcare professionals (HCPs)

	System factors				Information exchange practices				Quality improvement strategies			
**HCP**	**Time & resource constraints**	**Absence of handover communication training**	**Absence of structured formats for information exchange between HCPs**	**Hospital record keeping**	**Ad-hoc phone calls**	**Patient-held medical documents**	**Discharge instructions**	**Hierarchical transfer of responsibility**	**Increase resource provision**	**Introduce formal referral systems**	**Implement “e-health” systems**	**Patient-held booklet intervention**
**1**			✓			✓	✓		✓			
**2**			✓			✓	✓					
**3**						✓			✓	✓	✓	
**4**	✓		✓			✓	✓					
**5**			✓	✓		✓						
**6**	✓		✓						✓		✓	
**7**	✓		✓		✓							
**8**	✓				✓							
**9**									✓			
**11**	✓	✓	✓		✓		✓					
**14**	✓	✓	✓						✓	✓		
**15**				✓		✓			✓		✓	
**18**	✓										✓	✓
**19**	✓											✓
**20**	✓					✓						✓
**21**												✓
**22**	✓							✓				✓
**23**				✓		✓	✓	✓				
**24**	✓			✓								
**25**				✓								
**26**	✓							✓				

For peer review only

3.4 Overlapping content

3.4.1 Public healthcare constraints

During interviews, a number of patients reported that they chose to visit public hospitals because of the better availability of healthcare staff compared to local healthcare facilities, such as smaller hospitals and primary/community health centres:

> "*We have very limited time, we did go to local hospital but doctors are not there. So if we get time we will come here rather than going to a hospital where there are no doctors. (IP 15)*"

However, multiple patients also reported that government hospitals were often crowded with high daily patient loads:

> *"There is so much crowd there you can't ask or hear anything there… so many people are there now, you cannot do anything. (IP 11)"*

The human resource issues at government primary and community healthcare facilities were also mentioned by HCPs:

> *"It will be useful if availability of doctors is ensured at the peripheral institutions around the clock. At times it is not there. (DOC 1)"*

Additionally, in our study settings most hospital doctors worked in both outpatient clinics and inpatient wards on a daily basis. Many doctors expressed concerns of time pressures due to the large patient volumes seen at hospital outpatient clinics and the subsequent lack of time they had to attend to all patients:

> *"We can hardly spend five minutes with each patient, seeing the crowd you will just want to finish everyone soon. (DOC 7)"*

For peer review only - http://bmjopen.bmj.com/site/about/guidelines.xhtml

Some doctors also reported that human and medical resource constraints across public healthcare facilities were hindering quality of care:

"[It's] not [about] motivation, [it's about] resource limitation. It's not humanly possible to see people every day for seven days. Quality definitely gets compromised. (DOC 3)"

3.4.2 Referral communication

A number of patients who recalled being referred from a previous healthcare facility to the hospital reported that they were not provided with any referral information:

"No, they didn't give any parchi [papers]. We were getting medicines right only that is with us. (IP 8)"

HCPs also discussed referral communication. Doctors explained that there were no structured processes to follow for information exchange during referrals:

"Yeah there is no proper way of doing it… inpatients sometimes we have to [refer] but as I told you we never had a structured format. (DOC 14)"

However, despite a lack of structured systems, some doctors described making ad-hoc calls to ensure that some information was transferred when referring a patient:

"Sometimes I call the doctor to tell them that so and so is coming. Please do the needful. If I know the patient or doctor. (DOC 11)"

For peer review only - http://bmjopen.bmj.com/site/about/guidelines.xhtml

3.4.3 Patient-held medical information

At the point of hospital admission, patient-held notes and/or medical records can facilitate optimal care by providing HCPs with key patient-specific information. When asked about whether they brought medical papers to the hospital, most patients reported that they regularly stored and transported papers to HCP visits; these included referral notes, prescription cards, test results and other records from inpatient/outpatient/primary care:

> *“"Yeah we have always kept everything safely. [Shows researcher a bag with all sort of papers like reports, lab tests, etc.] (IP 3)”*

Doctors also talked about patient-held medical information during interviews. For example, some doctors reported that patients regularly kept and transported their medical records:

> *“Almost everyone comes with medical reports. (DOC 11)”*

However, other doctors described that, in their experience, the availability of patient-held records was less consistent and that this could negatively impact continuity of care:

> *“Some of them do bring investigations and all others don’t bring much and we have to work out what happened from the start. (DOC 3)”*

3.4.4 Healthcare management communication

When asked about verbal HCP communication, many patients reported that during admission and/or discharge a HCP had provided them with some basic verbal healthcare management information (i.e. medication, treatment, lifestyle and/or follow-up requirements). However, the quantity of information received appeared to vary notably between patients. For example, some recalled being given detailed instructions:

For peer review only - http://bmjopen.bmj.com/site/about/guidelines.xhtml

"Doctor says everything. I was given medicines and now they asked me to take injections also. Doctor is saying I am not controlling my sugar. The nurse taught me how to take injection. (IP 19)"

Conversely, others appeared to receive relatively limited information and one carer reported having to seek healthcare advice from alternative sources:

"Doctors don't explain everything. We speak to our friends and get details from them. (Carer - IP 16)"

HCPs also discussed their healthcare communication practices with inpatients. Whilst talking about discharge, a nurse described the usual amount of time taken to explain information to each patient:

"Usually we take 20-25 minutes to instruct the patients. If the patients understand then it can be even faster. (NUR 1)"

Doctors reported that they provided patients with documented information on discharge cards and verbally advised inpatients to return to their local HCP/healthcare institution during the discharge consultation:

"We give them a discharge card. Discharge card is there we have written and then we refer them to the local hospital or where they come from. (DOC 15)"

For peer review only - http://bmjopen.bmj.com/site/about/guidelines.xhtml

3.4.5 Booklet intervention

During interviews that took place after the handover expert meeting, patients were asked for their opinion regarding the utility of a patient-held booklet where records could be stored and transported to HCP visits. Most appeared to think that it might be effective and could help with self-management, including those who were illiterate:

> *"Yeah, sometimes we don't know what to do so it would be good if some paper is there to help us. We can't read it ourselves but our son or daughter-in-law can help us. (IP 17)"*

HCPs were also asked for their opinions regarding the booklet intervention. Many generally felt it could be useful, but various conditions and/or reservations were also expressed. For example, doctors felt that the success of the booklet would rely on patient attitudes:

> *"That will depend on the patients, if they maintain that and bring it every time. For us there is no change, we write our observations in paper or notebook, doesn't matter… Might be helpful. (DOC 22)"*

Related to this, one doctor felt that to see the most benefit, patients needed to be regularly instructed to keep and transport their medical documents:

> *"We write the communication but the patients don't keep them proper. I think we have to tell the patients to keep the letters and papers. (DOC 4)"*

For peer review only - http://bmjopen.bmj.com/site/about/guidelines.xhtml

3.5 Content unique to Inpatients

3.5.1 Attitudes regarding HCP communication during admission

A few patients recalled receiving some unfavourable/impolite treatment from healthcare staff during their hospital admission:

> *"The doctor's don't speak much. They explain but get angry if you don't understand them. (IP 3)"*

In addition, some patients expressed dissatisfaction with the lifestyle advice provided. In particular, patients of lower socio-economic status felt that nutritional instructions were not suitable for them due to their time and financial constraints:

> *"We are daily labourers we can't follow all the instructions… We can't follow that, we are poor we do hard work and we just can't concentrate on eating. Whatever is there we just eat. (IP 15)"*

3.6 Content unique to HCPs

3.6.1 Institutional/systemic factors

Despite displaying good knowledge of what information should be transferred during patient handovers, when asked about training opportunities numerous doctors mentioned that they had not received any formal handover training. Some recalled that this type of training was not provided at medical school:

> *"I think it was not there in medical curriculum. (IP 1)"*

For peer review only - http://bmjopen.bmj.com/site/about/guidelines.xhtml

Others reported that training was not provided in their workplace/s and instead they learned on the job:

> *"We are sent to the wards, we see what our seniors do and we do that's all. We have to develop our communication skills ourselves no formal training is there. (DOC 14)"*

When asked about hospital record keeping, a medical records officer stated that inpatient records are stored in hospitals following patient discharge for up to ten years. However, the same officer also indicated that these paper-based records are not easily accessible:

> *"Definitely I can locate any record but it might take some time to locate them. (MRO 1)"*

3.6.2 Organisational culture

Based on reports from both doctors and nurses, it appeared as though some hierarchical transfer of responsibility for documented handover communication took place in hospitals. For example, a senior doctor mentioned that they instructed medical interns to write notes for them when their patient load was high:

> *"We do write in the papers, whether it's discharge card or outpatient sheets. When patient load is high, then we tell our interns to do it for us, we check that and then sign. (DOC 22)"*

For peer review only - http://bmjopen.bmj.com/site/about/guidelines.xhtml

3.6.3 Requirements for improving information exchange

During interviews, HCPs were asked for their thoughts on intervention ideas to improve information exchange between HCPs and between HCPs and patients. Numerous doctors felt that there needed to be a notable increase in public healthcare resource provision:

> *"Infrastructure is very small but the outpatient department is ten times more than it can manage, so more posts should be created… We have to increase the manpower and also our materials. (DOC 15)"*

In addition, doctors discussed the idea of introducing structured referral documents and systems to improve referral communication:

> *"You can supply people with [referral] forms and make it mandatory that residents have to maintain a register. In that case they will maintain the register. (DOC 3)"*

Whilst discussing current information systems one doctor in Kerala reported that an application had been made for a near-future transition to computerised healthcare information systems. This appeared to be a state-wide plan for public healthcare facilities:

> *"We have submitted a proposal for paperless computerisation system for doctors, so I think state-wide they are planning to do that. (DOC 6)"*

For peer review only - http://bmjopen.bmj.com/site/about/guidelines.xhtml

4. DISCUSSION

4.1 Main findings

This study presents qualitative data on inpatient and HCP knowledge, attitudes and barriers to handover communication in public hospitals in Kerala and Himachal Pradesh states, India. Overall, the results show that verbal and documented information exchange between HCPs and between HCPs and patients is often suboptimal, with a lack of structured systems and HCP education in place to ensure sufficient continuity of care. Whilst three themes emerged for each participant group, comparison of the results showed that there was also a notable amount of overlapping content. These results have highlighted the multifaceted nature of handover communication within and between levels of healthcare in India. With regard to public health, the findings have also elucidated a number of key areas to address to improve the continuity and safety of chronic NCD patient care.

During interviews, both inpatients and HCPs recognised the resource constraints affecting public healthcare. The main issue reported was deficient primary healthcare services, which is in line with well-established findings of limited primary care infrastructure across India and numerous LMICs.[25] In our study settings, under-resourced primary care resulted in many patients preferring to visit hospitals. Subsequently, large patient loads were seen in outpatient and inpatient departments. Doctors reported that this limited their consultation times and ultimately affected the quality of information they could provide to patients. Notably varied patient recollections of healthcare information provided by HCPs during admission further evidenced the impact of time barriers on information exchange. The pressures reported here have also been identified in our previous outpatient research and may provide some explanation for the deficient provision of discharge information found in our quantitative inpatient study.[11] During interviews, senior doctors reported often being so busy

For peer review only - http://bmjopen.bmj.com/site/about/guidelines.xhtml

that they would pass the duty of writing discharge notes to interns or nurses. Additionally, it seemed that more time was spent on verbal communication; one nurse reported taking approximately twenty minutes per patient to explain discharge instructions. Such practices may be compromising the retention of key healthcare information, as global literature suggests that patients can struggle to absorb verbal details provided during consultations.[26] The potential implications of this are significant, given the associations we have found between failing to receive key documented discharge information and an increased likelihood of adverse events (Humphries, Jaganathan, Panniyammakal, *et al.* 2018).

Another topic discussed by both participant groups was referral communication. Reports revealed that that documented information was not always given to patients, and when it was, it was often in the form of minimal, hand-written notes on papers provided for other purposes (e.g. prescription cards). These findings reflect results from other LMIC studies that have evidenced the exchange of poor-quality referral documents.[27-29] A small number of doctors in our study reported calling HCPs to discuss a referral case, but this was dependent on how well they knew the patient and/or HCP. These deficits are perhaps unsurprising given that multiple HCPs reported that there was an absence of structured systems and education provided for handover communication. These findings concur with the few previous descriptions from India of an absence of training and protocols for handover practices.[8-10]

Another key area of discussion was the use of patient-held medical documents. Whilst many inpatients reportedly transported records to HCP visits, doctors recalled seeing many who did not bring information to hospital. This was problematic as if patients did not bring their records then doctors had to gather details from scratch. It was also reported that inpatient hospital records were not easily accessible and rarely retrieved. This lack of available and accurate medical information carries notable risks for patient safety, as without

For peer review only - http://bmjopen.bmj.com/site/about/guidelines.xhtml

key background and/or treatment details, critical oversights can be made that result in adverse events.[4 5 30] When asked about the potential utility of introducing patient-held record booklets, patients were generally positive and felt that the inclusion of self-management information would be beneficial. Doctors had mixed views but generally thought that they could be useful if patients had positive attitudes towards their maintenance and use. Given the unstructured, paper-based systems utilised across the study sites, this is a promising area for development. Patient-held records have been used successfully in maternity care throughout the world and have improved referral networks as well as patient satisfaction and knowledge.[17-21]

Other issues affecting handover communication were mentioned within each participant group. For inpatients, some recalled receiving impolite treatment from hospital doctors during admission. Additionally, a small number of patients were dissatisfied with the take-home nutritional advice provided, as they felt it failed to take into account their socio-economic deprivation. These results may be explained by the reported lack of communication training in medical education, as well as a historic tendency for paternalistic physician conduct in India.[31] In other areas of India and Asia, research on HCP-patient communication has also evidenced asymmetric power balances and patient dissatisfaction during both inpatient and outpatient consultations.[32] Such findings reveal the need for more patient-centred communication, particularly for the poorer patients that make up a significant proportion of public healthcare users. As for HCPs, during interviews many doctors recognised the need for an increase in public healthcare resource provision, as well as structured systems for information exchange. Some also discussed the promise of implementing "e-health" systems, with a doctor in Kerala reporting that public healthcare facilities across the state will be transitioning to computerised systems. Whilst our colleagues from Kerala report that this development is in its early stages, it has great potential as similar

For peer review only - http://bmjopen.bmj.com/site/about/guidelines.xhtml

systems have advanced the accessibility and quality of healthcare information across the globe.[33 34]

4.2 Strengths and limitations

A key strength of this study is that, as far as the authors are aware, it is the first to qualitatively report on handover communication with and between levels of care in India. The use of multiple sites and qualitative methodology has revealed a number of key issues that are supported among the HIC and emerging LMIC literature, suggesting likely transferability to other LMIC settings. Additionally, interviews with both patients and HCPs have provided an overview of handover communication from a multiple important perspectives. The number of interviews conducted helped to ensure data saturation for both participant groups and study credibility was strengthened via use of multi-analyst triangulation.[23]

With regard to limitations, recruitment challenges meant that patient participants were predominantly older (i.e. 45yrs+). Therefore, experiences of younger patients could not be thoroughly explored. In addition, the cross-cultural nature of this research may have resulted in constraints during data collection and analysis; for example, the presence of in-group bias could have affected participant's willingness to openly converse with a non-local researcher.[35] Social desirability bias from the use of individual interviews and participant's awareness that the interviewer was a public health professional may have also affected truthfulness of the data.[36] However, the recurrence of themes indicating data saturation and the finding that our results are supported by existing literature suggests this had minimal impact.

For peer review only - http://bmjopen.bmj.com/site/about/guidelines.xhtml

4.3 Conclusions and next steps

This study has found that handover communication for chronic NCD patients during care transitions and hospital admission is often fragmented. The critical barriers appear to be an absence of structured information exchange systems and HCP education. There is also a growing need for the government to strengthen the primary healthcare infrastructure in line with the declaration of Alma Alta.[37] This will greatly assist in increasing accessibility of care and reduce pressure on hospital services. It will also be required to address the United Nations sustainable development goals regarding universal health coverage and reducing premature deaths from NCDs.[38] In addition, the implementation of structured documentation, systems and training is urgently required to manage care transitions such as referral and discharge. Research from both HIC and LMIC settings has proven that such interventions can improve continuity and safety of care.[4 10 27 39]

During HCP interviews, it was reported that public healthcare facilities in Kerala will transitioning to computerised “e-health” information systems. Whilst this development holds promise, issues regarding patient access to healthcare information and information exchange between public and private providers are likely to remain an issue. Therefore, with regard to research recommendations, a mixed-methods pilot study exploring the design and implementation of patient-held record booklets is suggested. This idea was welcomed by Indian national and international experts, as well as patients and HCPs in our study areas and could improve patient self-management as well as communication and integration between HCPs.

Finally, given the rising burden of NCDs across LMICs, this research is timely and crucial for effective health systems development. It is important that further LMIC research is conducted to explore critical factors affecting quality, continuity and safety of care and to develop sustainable and cost-effective interventions.

For peer review only - http://bmjopen.bmj.com/site/about/guidelines.xhtml

ACKNOWLEDGEMENTS

This research was supported by the National Institute for Health Research (NIHR) Collaboration for Leadership in Applied Health Research and Care West Midlands (NIHR CLAHRC WM). We would like to extend our thanks to all patients, healthcare staff and researchers who kindly took the time to participate in this project. We are also indebted and give thanks to the participating hospitals and community healthcare facilities. Finally, we are very grateful to the Directors of Health from both Himachal Pradesh and Kerala States, India, for their assistance in facilitating this project. Without their support this research would not have been possible.

AUTHOR CONTRIBUTIONS

In order of the authors list: CH: Data curation, formal analysis, visualisation, writing (both original draft and final review and editing). SJ: Investigation, data curation, project administration, writing (review and editing). JP: Conceptualisation, funding acquisition, project administration, supervision, writing (review and editing). SS: Funding acquisition, project administration, supervision, writing (review and editing). SG(oenka): Funding acquisition, projection administration, supervision, writing (review and editing). PD: Conceptualisation, funding acquisition, project administration, supervision, writing (review and editing). PG: Conceptualisation, data curation, funding acquisition, writing (review and editing). SG(reenfield): Funding acquisition, formal analysis, writing (review an editing). RL: Conceptualisation, funding acquisition, writing (review and editing). SMH:

For peer review only - http://bmjopen.bmj.com/site/about/guidelines.xhtml

Conceptualisation, funding acquisition, investigation, methodology, project administration, supervision, writing (review and editing).

FUNDING STATEMENT

This research was joint-funded by the Department of International Development, the Economic and Social Research Council, the Medical Research Council and the Wellcome Trust - grant number: MR/M00287X/1. The funders had no role in study design, conduct or reporting.

COMPETING INTERESTS STATEMENT

The authors of this manuscript have no competing interests to disclose.

ETHICS APPROVAL

This study was reviewed and approved by the Centre for Chronic Disease Control Independent Ethics Committee, India, and the Amrita Institute of Medical Sciences Institutional Ethics Committee, India. Data archives will be stored at the University of Birmingham, in accordance with the University's code of practice.

For peer review only

For peer review only - http://bmjopen.bmj.com/site/about/guidelines.xhtml

DATA SHARING STATEMENT

No additional data are available.

For peer review only

For peer review only - http://bmjopen.bmj.com/site/about/guidelines.xhtml

REFERENCES

1. Islam SMS, Purnat TD, Phuong NTA, et al. Non-Communicable Diseases (NCDs) in developing countries: a symposium report. *Globalization and health* 2014;10(1):81.
2. World Health Organisation. Communication during patient hand-overs. *Patient safety solutions* 2007;1(3):1-4.
3. British Medical Association. Safe handover: safe patients. *Guidance on clinical handover for clinicians and managers London: BMA* 2004;7:141.
4. Merten H, van Galen LS, Wagner C. Safe handover. *Bmj* 2017;359:j4328. doi: https://dx.doi.org/10.1136/bmj.j4328
5. Safety ACf, Care QiH. Clinical handover and patient safety: Literature review report. 2005
6. Hesselink G, Schoonhoven L, Barach P, et al. Improving patient handovers from hospital to primary care: a systematic review. *Ann Intern Med* 2012;157(6):417-28. doi: https://dx.doi.org/10.7326/0003-4819-157-6-201209180-00006
7. Bradley S, Mott S. Adopting a patient-centred approach: an investigation into the introduction of bedside handover to three rural hospitals. *J Clin Nurs* 2014;23(13-14):1927-36. doi: https://dx.doi.org/10.1111/jocn.12403
8. Kumar PJ, V.; Vij, A.; Gupta, S K. . Who is more hands on with hand-offs? A comparative study of clinical handovers among doctors and nurses in a tertiary care center in India. *International Journal of Research Foundation of Hospital & Healthcare Administration* 2015;3(1):33-40.
9. Kumar P, Jithesh V, Vij A, et al. Need for a hands-on approach to hand-offs: A study of nursing handovers in an Indian Neurosciences Center. *Asian journal of neurosurgery* 2016;11(1):54.
10. Rajavelu P, Rajagopalan A. Pre-formatted written discharge summary—a step towards quality assurance in the emergency department. *International journal of emergency medicine* 2008;1(4):321-25.
11. Humphries C, Jaganathan S, Panniyammakal J, et al. Investigating clinical handover and healthcare communication for outpatients with chronic disease in India: A mixed-methods study. *PLoS One* Forthcoming
12. Marshall MN. Sampling for qualitative research. *Fam Pract* 1996;13(6):522-26.
13. Palys T. Purposive Sampling. In: Given LM, ed. The Sage Encyclopedia of Qualitative Research Methods. Los Angeles: Sage 2008:697-98.
14. World Health Organistion. Information for Researchers Concerning Informed Decision Making [Internet]. Available from: http://www.who.int/rpc/research_ethics/Process_seeking_IF_printing2.pdf accessed 01/05/18.
15. Seale C. Quality in qualitative research. *Qualitative inquiry* 1999;5(4):465-78.
16. Baker SE, Edwards R, Doidge M. How many qualitative interviews is enough?: Expert voices and early career reflections on sampling and cases in qualitative research. 2012
17. Shah PM, Selwyn BJ, Shah K, et al. Evaluation of the home-based maternal record: a WHO collaborative study. *Bull World Health Organ* 1993;71(5):535-48.
18. Osaki K, Hattori T, Kosen S. The role of home-based records in the establishment of a continuum of care for mothers, newborns, and children in Indonesia. *Glob Health Action* 2013;6:1-12. doi: https://dx.doi.org/10.3402/gha.v6i0.20429
19. Turner KE, Fuller S. Patient-held maternal and/or child health records: meeting the information needs of patients and healthcare providers in developing countries? *Online journal of public health informatics* 2011;3(2)
20. Takeuchi J, Sakagami Y, Perez RC. The Mother and Child Health handbook in Japan as a health promotion tool: An overview of its history, contents, use, benefits, and global influence. *Global pediatric health* 2016;3:2333794X16649884.

For peer review only - http://bmjopen.bmj.com/site/about/guidelines.xhtml

21. Walton S, Bedford H, Dezateux C. Use of personal child health records in the UK: findings from the millennium cohort study. *Bmj* 2006;332(7536):269-70.
22. Spencer L, Ritchie J. Qualitative data analysis for applied policy research. Analyzing qualitative data: Routledge 2002:187-208.
23. Lincoln YS, Guba EG. Naturalistic inquiry. Beverley Hills: ca: sage, 1985.
24. Gale NK, Heath G, Cameron E, et al. Using the framework method for the analysis of qualitative data in multi-disciplinary health research. *BMC medical research methodology* 2013;13(1):117.
25. Das J, Hammer J. Quality of primary care in low-income countries: facts and economics. *Annu Rev Econ* 2014;6(1):525-53.
26. Kessels RP. Patients' memory for medical information. *Journal of the Royal Society of Medicine* 2003;96(5):219-22.
27. Janati A, Amini A, Adham D, et al. Assessing the quality of referral letters written by general practitioners: a cross-sectional study in rural Iran. *Cadernos de Saude Publica* 2017;33(2):e00043016.
28. Gyedu A, Baah EG, Boakye G, et al. Quality of referrals for elective surgery at a tertiary care hospital in a developing country: an opportunity for improving timely access to and cost-effectiveness of surgical care. *Int J Surg* 2015;15:74-8. doi: https://dx.doi.org/10.1016/j.ijsu.2015.01.033
29. Osinaike BO, Esezobor CI, Akinsola OJ. Quality of Referral Letters to the Paediatric Department of a Tertiary Hospital in Nigeria. *Nigerian Quarterly Journal of Hospital Medicine* 2013;23(4):273-9.
30. Forster AJ, Murff HJ, Peterson JF, et al. The incidence and severity of adverse events affecting patients after discharge from the hospital. *Ann Intern Med* 2003;138(3):161-7.
31. Ghooi R, Deshpande S. Patients' rights in India: an ethical perspective. *Indian J Med Ethics* 2012;9(277):e8.
32. Pun J, Chan EA, Wang S, et al. Health professional-patient communication practices in East Asia: An integrative review of an emerging field of research and practice in Hong Kong, South Korea, Japan, Taiwan, and Mainland China. *Patient education and counseling* 2018
33. Piette JD, Lun KC, Moura LA, Jr., et al. Impacts of e-health on the outcomes of care in low- and middle-income countries: where do we go from here? *Bull World Health Organ* 2012;90(5):365-72. doi: https://dx.doi.org/10.2471/BLT.11.099069
34. Campanella P, Lovato E, Marone C, et al. The impact of electronic health records on healthcare quality: a systematic review and meta-analysis. *Eur J Public Health* 2016;26(1):60-4. doi: https://dx.doi.org/10.1093/eurpub/ckv122
35. Tajfel H. Social psychology of intergroup relations. *Annual review of psychology* 1982;33(1):1-39.
36. Spector P. Social desirability bias. *Encyclopedia of social science research methods* 2004:1045-46.
37. Organization WH. Declaration of Alma-Ata: International Conference on Primary Health Care, Alma-Ata, USSR, 6–12 September 1978. *Retrieved February* 1978;14:2006.
38. United Nations. Sustainable Development Goals. [Internet]. Available from: https://www.un.org/sustainabledevelopment/sustainable-development-goals/2018.
39. Ramanayake R. Structured printed referral letter (form letter); saves time and improves communication. *Journal of family medicine and primary care* 2013;2(2):145.

For peer review only - http://bmjopen.bmj.com/site/about/guidelines.xhtml

S1. Additional information regarding the Indian healthcare system

1. National context: structure of public healthcare system

The basic structure of the public healthcare system in India is as follows:[1]

- National level: Ministry of Health and Family Welfare.
- State level: State Department of Health and Family welfare in each state.
- Regional level: covers 3 - 5 districts. Headed by State Directorate of Health.
- District level: Middle level management organisation serving as a link between the regional and state structures and the peripheral and PHC structures.
- Sub-divisional/Taluk level: Hospitals/hospitals with specialty care (Taluk headquarters hospitals). Healthcare services are rendered via the office of Assistant District Health and Family Welfare Officer.
- Community level: CHCs that cater for 80,000-120,000 population and PHCs that cover approximately 20,000 – 30,000 population (often upgrades of rural dispensaries).

For peer review only

2. Public healthcare across India

The quality of public healthcare across India varies notably between states and between urban and rural areas. Aside from some pockets of excellence in a select few states, the public sector is generally falling short of meeting the basic healthcare needs of the growing population. Some of the main reasons for this include: services being too far away, a lack of trained personnel and supplies, and limited facility opening times that are often unreliable.[2] With regard to primary health centres, government estimates indicate that 10% are without a

doctor, 37% are without a laboratory technician and 25% are without a pharmacist.[3] Issues with public health centres are particularly rife within poor communities, where facilities have been found to be closed more than half the time and lack basic medical supplies. Public facilities are the often the only source of qualified healthcare professionals in rural areas, which is where much of the poor live.[2]

3. National context: private healthcare

Public healthcare in India has lacked funding over a series of decades, resulting from a lack of prioritisation from economic planners. Therefore, the increasing prevalence of chronic, non-communicable diseases alongside unresolved challenges of infectious diseases has placed more strain on public health systems than what can feasibly be managed. The private healthcare sector has subsequently proliferated to meet rising needs, expectations and incomes and surveys indicate that private healthcare providers now dominate service provision. According to reports, public facilities provided just 20% of primary and community-level healthcare services, and 40% of hospital visits in 2004-05 (down from 25% and 60% respectively in 1986-87).[2] At the higher end of the market the private sector has world class facilities that have grown substantially. As a result, hospital care is now an export sector for medical tourism that cares for approximately 200,000 foreign patients per year.[4] However, private healthcare providers are poorly regulated, with uneven quality across facilities. This is resulting in a large number of private facilities delivering services without appropriate equipment or expertise. Additionally, although visiting private providers is preferential for many, it is common for high out-of –pocket costs to be incurred; more than 40% of all private hospital inpatients have to borrow money or sell assets in order to fund their care.[5] This means that many poorer patients are unable to access healthcare while others fall into poverty as a result of spending.

For peer review only

REFERENCES

1. Care Continuum Alliance, India. India – Health Care System Structure and Organization. 2009. Available from: http://dmaa.pbworks.com/w/page/17960772/India%20-%20Health%20Care%20System%20Structure%20and%20Organization

2. Joumard I, Kumar A. Improving health outcomes and health care in India. Paris: Organisation for Economic Cooperation and Development (OECD) Working Papers, No. 1184, OECD Publishing. 2015. Available from: http://www.oecd-ilibrary.org/economics/improving-health-outcomes-and-health-care-in-india_5js7t9ptcr26-en

3. Ministry of Health and Family Welfare, Government of India. Rural Health Statistics in India. 2012. Available from: https://nrhm.gujarat.gov.in/images/pdf/publications/RHS_2012.pdf

4. Lunt N, Smith R, Exworthy M, Green S, Horsfall D, Mannion R. Medical Tourism: Treatments, Markets and Health System Implications: a Scoping Review. OECD Publishing. 2010. Available from: Paris http://www.oecd.org/els/health-systems/48723982.pdf

5. The World Bank - Health, Nutrition, Population Sector Unit India South Asian Region. Raising the sights: better health systems for India's poor. Available from: https://openknowledge.worldbank.org/handle/10986/14080

For peer review only

S2. S1. Additional information regarding the study settings

2.1.1. Study setting - Himachal Pradesh

Himachal Pradesh is principally a rural state in northern India. It has a population of 6.86 million people and the average literacy rate is 83.3%, which is higher than the national average (74%). However, rates remain notably lower for women compared to men (76.6% vs. 90.8%, respectively).[6] Private HCPs are less prevalent in Himachal Pradesh and public healthcare utilisation remains relatively high.[7] A recent study found that the availability of public health services in the state was deemed adequate as compared to standards of other hill states, but with an unequal distribution of resources across regions.[8]

2.1.2 Study setting - Kerala

Kerala state is in the south-west of India. It has a population of 34.8 million people and a greater than national average urban-based population of 47.7%. It has the highest overall literacy rate in India (93.9%; men 96.1%, women 92.1).[9] There are a relatively large number of government healthcare facilities in Kerala but the healthcare environment is becoming increasingly complex due to a growing presence of private healthcare providers. Despite this, public health facilities in Kerala are generally the first point of care and continue to deliver essential services.[10]

For peer review only

REFERENCES

6. Government of India. Himachal Pradesh Population Census Data 2011 [Internet]. Available from: https://www.census2011.co.in/census/state/himachal+pradesh.html accessed 13/06/18 2017.

7. Government of India. Himachal Pradesh Development Report. New Delhi: Academic Foundation, 2005.

8. Suman.; Bhutani S. Availability of health services in Himachal Pradesh *International Journal of Advanced Research and Development* 2017;2(6):231-38.

9. Government of Kerala. Kerala Population Census Data 2011 [Internet]. Available from: https://www.census2011.co.in/census/state/kerala.html accessed 13/06/18.

10. Government of Kerala. Kerala Development Report. New Delhi: Academic Foundation, 2008.

For peer review only

S3. Qualitative interview topic guides (Dec 2014 – Oct 2015)

Interview topic guide for healthcare providers in the hospital (and community providers – reverse the order of asking section A and B)

Please explain your experience or observations in the circumstances below with patients with cardiovascular conditions, diabetes, chronic chest disease or other chronic conditions in your place of work:

A - Primary to secondary care:

1. In your experience, how frequently do patients bring notes from primary care when they come to the hospital? What type of note is it? (Forms, letters, prescription etc.)
2. Usually what type of information does it contain? (medication, past history, tests, etc.)
3. In what way is this referral information helpful/could be helpful, and how could it be improved? What do you think are the most important details to include in the referral information?
4. In the absence of patient records or referral notes, how do you decide what the patient has been taking medically?
5. How would you improve this situation – guide us on the problems and things that would help?
6. If these problems are sorted, how do you think we can encourage community and primary care doctors to send the information when they refer a patient? (i.e. motivators)

B - Secondary to Primary care:

7. If you or your colleagues have a system of sending information about the patient to the primary care providers (even if you do not know who this may be), what form does this information take?
8. In your opinion, what way is the information you send to primary care doctors helpful for the care of the patient, and how could these communications be improved?
9. When seeing patients at outpatient clinics, given the fact that the patients may not come back, what do you do to ensure they continue their correct treatment?
10. How would you improve this situation – guide us on the problems and things that would help?

For peer review only

Interview topic guide for healthcare providers (continued)

11. If these problems are sorted, how do you think we can encourage hospital doctors to send the information when they refer a patient? (motivators)

C - Training:

12. As far as you recall, as an undergraduate or while working, have you had any structured training for hospital shifts or for transfer for information at shift times in hospital practice? If so, what were you trained to do?

13. As far as you recall, as an undergraduate or while working, have you had any structured training for writing referral notes when referring patients to hospitals or for writing handover notes for primary care doctors to pass on patient's clinical information to them? If so, what where you trained to do?

D - Health care provider information to be collected:

- Speciality
- Years of relevant experience
- Place of work
- Designation

For peer review only

Interview topic guide for patients and carers

Please explain your experience or observations in the below circumstances with health care providers in your town in small clinics near you or at hospitals:

A - Primary to secondary care:

1. Is there any way that your treating community doctor tells the hospital about your condition when they refer you there?

2. Do you think this is important and why? (please explore if they think it is or is not important)

3. How do you share information with the hospital doctor about your previous medical treatments and conditions (in other hospitals or when seeing your local doctors)?

Interview topic guide for patient and carer (continued)

B - Secondary to primary care:

a. How does your local treating doctor/close to your house, get to know about what was done in hospital when you were seen at the outpatient clinic and/or admitted?

b. Do you think this is important and why?

c. What do you do with your medical papers when you get home after being seen at the outpatient clinic?

C - Secondary care to patient:

d. What advice/instructions are you given when you see the doctor at the outpatient clinic?

e. After you leave hospital, how do you know what to do to take best care of yourself (for the months ahead)?

D - Participant information to be collected:

- Age
- Gender
- Religion
- Highest level of education
- Condition(s)
- Years of having this conditions
- Place village and district

For peer review only

S4. Qualitative interview topic guides (Oct 2015 – Dec 2015)

Interview topic guide for healthcare providers in the hospital (and community providers – reverse the order of asking section A and B)

Note to Researchers: if a healthcare provider states they have limited time to be interviewed, please use the shortened topic guide below.

Please explain your experience or observations in the circumstances below with patients with cardiovascular conditions, diabetes, chronic chest disease or other chronic conditions in your place of work:

A - Primary to secondary care:

14. In your experience, how frequently do patients bring notes from primary care when they come to the hospital? What type of note is it? (Forms, letters, prescription etc.)

15. Usually what type of information does it contain? (medication, past history, tests, etc.)

16. In what way is this referral information helpful/could be helpful, and how could it be improved? What do you think are the most important details to include in the referral information?

17. In the absence of patient records or referral notes, how do you decide what the patient has been taking medically?

18. How would you improve this situation – guide us on the problems and things that would help? Do you think patient-held booklets for storing and transporting medical records would be useful?

19. If these problems are sorted, how do you think we can encourage community and primary care doctors to send the information when they refer a patient? (i.e. motivators)

B - Secondary to Primary care:

20. If you or your colleagues have a system of sending information about the patient to the primary care providers (even if you do not know who this may be), what form does this information take?

21. In your opinion, what way is the information you send to primary care doctors helpful for the care of the patient, and how could these communications be improved?

For peer review only

Interview topic guide for healthcare providers (continued)

22. When seeing patients at outpatient clinics, given the fact that the patients may not come back, what do you do to ensure they continue their correct treatment?

23. How would you improve this situation – guide us on the problems and things that would help? Do you think patient-held booklets for storing and transporting medical records would be useful?

24. If these problems are sorted, how do you think we can encourage hospital doctors to send the information when they refer a patient? (motivators)

C - Training:

25. As far as you recall, as an undergraduate or while working, have you had any structured training for hospital shifts or for transfer for information at shift times in hospital practice? If so, what were you trained to do?

26. As far as you recall, as an undergraduate or while working, have you had any structured training for writing referral notes when referring patients to hospitals or for writing handover notes for primary care doctors to pass on patient's clinical information to them? If so, what where you trained to do?

D - Health care provider information to be collected:

- Speciality
- Years of relevant experience
- Place of work
- Designation

Shortened interview topic guide for healthcare providers

1. Would you prefer it if patients came to consultations with all their previous medical records?

2. Will having previous records of patients in a booklet format help with clinical management?

3. Do you think that introducing patient-held booklets for recording key information (e.g. blood pressure, sugar levels etc.) would help clinical management?

4. Would you like to write in patient-held booklets about patients' clinical management?

5. Please share your views on how to improve the clinical management of chronic non-communicable disease patients

For peer review only

Shortened interview topic guide for healthcare providers (continued)

Health care provider information to be collected:

- Speciality
- Years of relevant experience
- Place of work
- Designation

For peer review only

Interview topic guide for patients and carers

Note to Researchers: if a patient states they have limited time to be interviewed, please use the shortened topic guide below.

Please explain your experience or observations in the below circumstances with health care providers in your town in small clinics near you or at hospitals:

A - Primary to secondary care:

4. Is there any way that your treating community doctor tells the hospital about your condition when they refer you there?

5. Do you think this is important and why? (please explore if they think it is or is not important)

6. How do you share information with the hospital doctor about your previous medical treatments and conditions (in other hospitals or when seeing your local doctors)?

B - Secondary to primary care:

a. How does your local treating doctor/close to your house, get to know about what was done in hospital when you were admitted?

b. Do you think this is important and why?

c. What do you do with your medical papers when you get home after being seen at the hospital?

Interview topic guide for patients and carers (continued)

C - Secondary care to patient:

d. What advice/instructions are you given when you see the doctor at the hospital?

e. After you leave hospital, how do you know what to do to take best care of yourself (for the months ahead)?

f. Do you think that having a patient-held booklet for storing and transporting medical records would be useful? Would you carry a record booklet to healthcare appointments?

g. Do you think that having a record booklet would help your self-management?

h. Would you like to get detailed written information about your medicines, follow-up information and advice on lifestyle and self-management?

i. Do you think that having information sheets would help your self-management?

D - Participant information to be collected:

- Age
- Gender
- Religion
- Highest level of education
- Condition(s)
- Years of having this conditions
- Place village and district

For peer review only

Shortened interview topic guide for patients and carers

1. What advice/instructions are you given when you see the doctor at the outpatient clinic?

2. Do you think that having a patient-held booklet for storing and transporting medical records would be useful? Would you carry a record booklet to healthcare appointments?

3. Do you think that having a record booklet would help you to manage your self-care at home?

4. Do you think that having information sheets would help you to manage your self-care at home?

5. Would you like to get detailed written information about your medicines, follow-up and lifestyle advice?

Participant information to be collected:

- Age
- Gender
- Religion
- Highest level of education
- Condition(s)
- Years of having this conditions
- Place village and district

For peer review only

S5. Figure 1. Similarities and differences between the content of IP & HCP data with related sub-categories

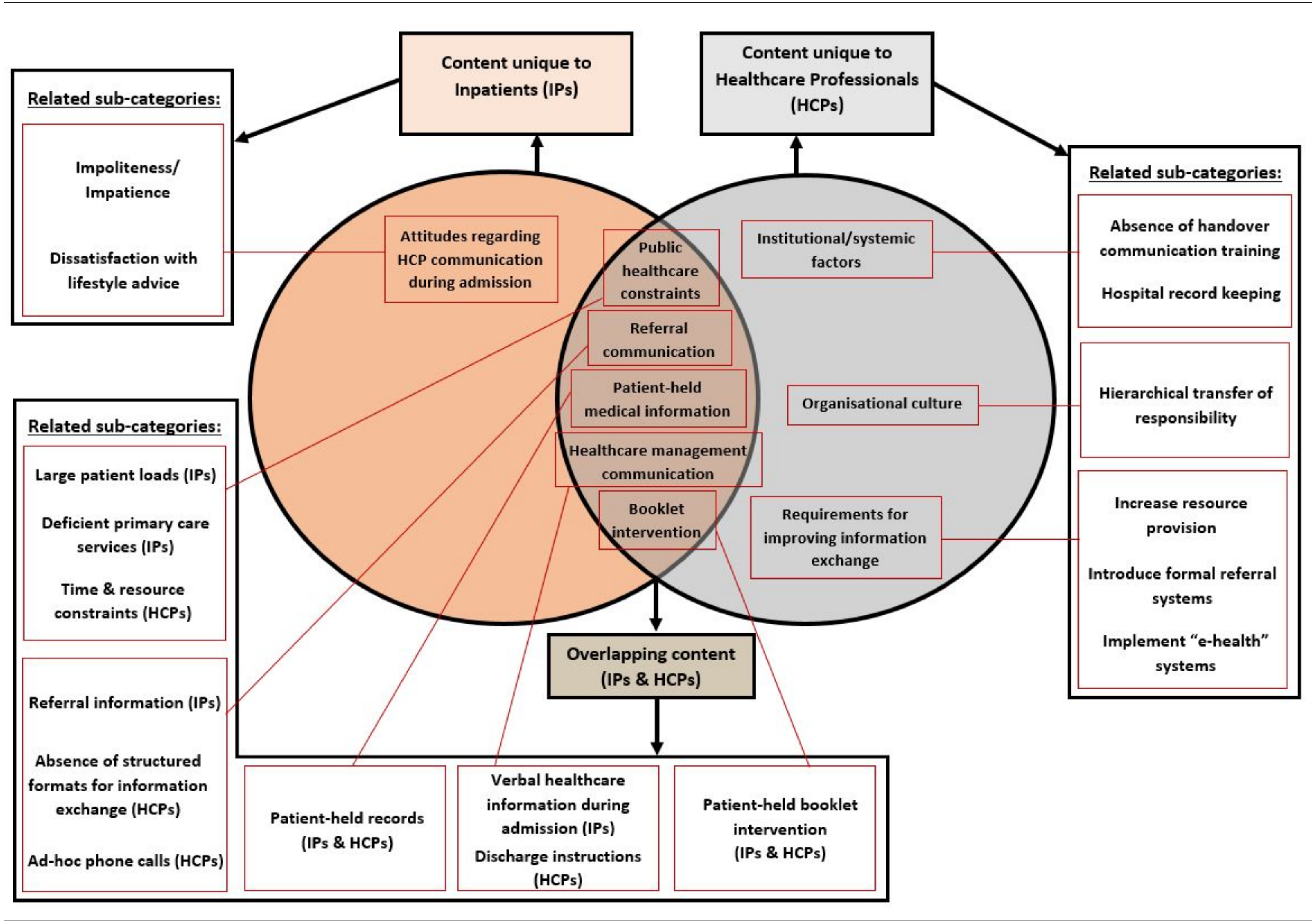

Reporting checklist for qualitative study.

Based on the SRQR guidelines.

Instructions to authors

Complete this checklist by entering the page numbers from your manuscript where readers will find each of the items listed below.

Your article may not currently address all the items on the checklist. Please modify your text to include the missing information. If you are certain that an item does not apply, please write "n/a" and provide a short explanation.

Upload your completed checklist as an extra file when you submit to a journal.

In your methods section, say that you used the SRQR reporting guidelines, and cite them as:

O'Brien BC, Harris IB, Beckman TJ, Reed DA, Cook DA. Standards for reporting qualitative research: a synthesis of recommendations. Acad Med. 2014;89(9):1245-1251.

		Reporting Item	Page Number
	#1	Concise description of the nature and topic of the study identifying the study as qualitative or indicating the approach (e.g. ethnography, grounded theory) or data collection methods (e.g. interview, focus group) is recommended	1
	#2	Summary of the key elements of the study using the abstract format of the intended publication; typically	2-3

For peer review only

		includes background, purpose, methods, results and conclusions	
Problem formulation	#3	Description and signifcance of the problem / phenomenon studied: review of relevant theory and empirical work; problem statement	4-5
Purpose or research question	#4	Purpose of the study and specific objectives or questions	5
Qualitative approach and research paradigm	#5	Qualitative approach (e.g. ethnography, grounded theory, case study, phenomenolgy, narrative research) and guiding theory if appropriate; identifying the research paradigm (e.g. postpositivist, constructivist / interpretivist) is also recommended; rationale. The rationale should briefly discuss the justification for choosing that theory, approach, method or technique rather than other options available; the assumptions and limitations implicit in those choices and how those choices influence study conclusions and transferability. As appropriate the rationale for several items might be discussed together.	6-10
Researcher characteristics and reflexivity	#6	Researchers' characteristics that may influence the research, including personal attributes, qualifications / experience, relationship with participants, assumptions and / or presuppositions; potential or actual interaction between researchers' characteristics and the research	7-10

For peer review only

		questions, approach, methods, results and / or transferability	
Context	#7	Setting / site and salient contextual factors; rationale	6
Sampling strategy	#8	How and why research participants, documents, or events were selected; criteria for deciding when no further sampling was necessary (e.g. sampling saturation); rationale	6-7
Ethical issues pertaining to human subjects	#9	Documentation of approval by an appropriate ethics review board and participant consent, or explanation for lack thereof; other confidentiality and data security issues	32
Data collection methods	#10	Types of data collected; details of data collection procedures including (as appropriate) start and stop dates of data collection and analysis, iterative process, triangulation of sources / methods, and modification of procedures in response to evolving study findings; rationale	7-9
Data collection instruments and technologies	#11	Description of instruments (e.g. interview guides, questionnaires) and devices (e.g. audio recorders) used for data collection; if / how the instruments(s) changed over the course of the study	7-9
Units of study	#12	Number and relevant characteristics of participants, documents, or events included in the study; level of participation (could be reported in results)	11-13

For peer review only

Data processing	#13	Methods for processing data prior to and during analysis, including transcription, data entry, data management and security, verification of data integrity, data coding, and anonymisation / deidentification of excerpts	9-15
Data analysis	#14	Process by which inferences, themes, etc. were identified and developed, including the researchers involved in data analysis; usually references a specific paradigm or approach; rationale	9-10
Techniques to enhance trustworthiness	#15	Techniques to enhance trustworthiness and credibility of data analysis (e.g. member checking, audit trail, triangulation); rationale	9-10
Syntheses and interpretation	#16	Main findings (e.g. interpretations, inferences, and themes); might include development of a theory or model, or integration with prior research or theory	11-25
Links to empirical data	#17	Evidence (e.g. quotes, field notes, text excerpts, photographs) to substantiate analytic findings	18-25
Intergration with prior work, implications, transferability and contribution(s) to the field	#18	Short summary of main findings; explanation of how findings and conclusions connect to, support, elaborate on, or challenge conclusions of earlier scholarship; discussion of scope of application / generalizability; identification of unique contributions(s) to scholarship in a discipline or field	26-29

For peer review only

Limitations	#19	Trustworthiness and limitations of findings	29
Conflicts of interest	#20	Potential sources of influence of perceived influence on study conduct and conclusions; how these were managed	32
Funding	#21	Sources of funding and other support; role of funders in data collection, interpretation and reporting	32

The SRQR checklist is distributed with permission of Wolters Kluwer © 2014 by the Association of American Medical Colleges. This checklist was completed on 26. November 2018 using http://www.goodreports.org/, a tool made by the EQUATOR Network in collaboration with Penelope.ai

For peer review only

BMJ Open

Patient and healthcare provider knowledge, attitudes and barriers to handover and healthcare communication during chronic disease inpatient care in India: A qualitative exploratory study

Journal:	*BMJ Open*
Manuscript ID	bmjopen-2018-028199.R1
Article Type:	Original research
Date Submitted by the Author:	13-Sep-2019
Complete List of Authors:	Humphries, Claire; University of Birmingham, UK, Institute of Applied Health Research<br>Jaganathan, Suganthi ; Centre for Chronic Disease Control<br>Panniyammakal, Jeemon ; Centre for Chronic Disease Control<br>Singh, Sanjeev; Amrita Institute of Medical Sciences and Research Centre; Amrita Institute of Medical Sciences and Research Centre<br>Goenka, Shifalika; Public Health Foundation of India<br>Dorairaj, Prabhakaran ; Centre for Chronic Disease Control<br>Gill, Paramjit ; University of Warwick<br>Greenfield, Sheila; University of Birmingham, Primary Care Clinical Sciences<br>Lilford, Richard; University of Warwick, Division of Health and Population Sciences<br>Manaseki-Holland, Semira; University of Birmingham,
**Primary Subject Heading**:	Public health
Secondary Subject Heading:	Health services research, Qualitative research
Keywords:	International health services < HEALTH SERVICES ADMINISTRATION & MANAGEMENT, Quality in health care < HEALTH SERVICES ADMINISTRATION & MANAGEMENT, PUBLIC HEALTH, QUALITATIVE RESEARCH

SCHOLARONE™
Manuscripts

Patient and healthcare provider knowledge, attitudes and barriers to handover and healthcare communication during chronic disease inpatient care in India: A qualitative exploratory study

Claire Humphries[1¶], Suganthi Jaganathan[2,3¶], Jeemon Panniyammakal[2,3,4], Sanjeev Singh[5], Shifalika Goenka[2,3], Prabhakaran Dorairaj[2,3], Paramjit Gill[5], Sheila Greenfield[1], Richard Lilford[6], Semira Manaseki-Holland[1*]

[1] Institute of Applied Health Research, University of Birmingham, Birmingham, West Midlands, United Kingdom

[2] Public Health Foundation of India, New Delhi, Delhi, India

[3] Centre for Chronic Disease Control, New Delhi, Delhi, India

[4] Sree Chitra Tirunal Institute of Medical Sciences and Technology, Trivandrum, Kerala, India

[5] Hospital Administration, Amrita Institute of Medical Sciences, Kochi, Kerala, India

[6] Centre for Applied Health Research and Delivery, University of Warwick, Warwick, Warwickshire, United Kingdom

*Corresponding author

Email: S.ManasekiHolland@bham.ac.uk

¶ These authors contributed equally to this work.

For peer review only - http://bmjopen.bmj.com/site/about/guidelines.xhtml

ABSTRACT

Objectives To investigate patient and healthcare provider (HCP) knowledge, attitudes and barriers to handover and healthcare communication during inpatient care and explore interventions for improving storage and transfer of healthcare information.

Methods *Design:* Qualitative study comprising 41 semi-structured, individual interviews. Thematic analysis using the Framework Method with analyst triangulation. *Setting*: Three hospitals in Himachal Pradesh and Kerala, India. *Participants:* 20 male (n=10) and female (n=10) chronic non-communicable disease (NCD) patients and 21 male (n=15) and female (n=6) HCPs. Purposive sampling was used to identify patients with chronic NCDs (Chronic Respiratory Disease, Cardiovascular Disease, Diabetes or Hypertension) and HCPs.

Results Chronic NCD patient themes: (1) *Public healthcare service characteristics;* (2) *HCP-patient communication;* (3) *Attitudes regarding medical information.* HCP themes: (1) *System factors;* (2) *Information exchange practices;* (3) *Quality improvement strategies.* Both patients and HCPs recognised public healthcare constraints that increased pressure on hospitals and subsequently limited consultation times. Systemic issues reported by HCPs were a lack of formal handover systems, training and accessible hospital-based records. Healthcare management communication during admission was inconsistent and lacked patient-centeredness, evidenced by patient reports of varying levels of information received and some dissatisfaction with lifestyle advice. Senior doctors reported passing the writing discharge notes to juniors when busy with high patient loads. Nurses reported providing the majority of discharge instructions to patients verbally. Patient-held

For peer review only - http://bmjopen.bmj.com/site/about/guidelines.xhtml

documents served as a vehicle for information exchange between HCPs, but were not always transported. HCPs and patients expressed positive views towards introducing patient-held booklets to improve organisation and transfer of documents.

Conclusions Handover and healthcare communication during chronic NCD inpatient care is suboptimal. Structured information exchange systems and HCP training are required to improve continuity and safety of care during critical transitions such as referral and discharge. Findings suggest that patient-held booklets may also assist in enhancing handover and patient-centred practices.

For peer review only - http://bmjopen.bmj.com/site/about/guidelines.xhtml

STRENGTHS AND LIMITATIONS OF THIS STUDY

- As far as the authors are aware, this is the first study to qualitatively explore factors affecting multiple areas of handover communication for chronic disease inpatients in India.
- The number of interviews conducted with both patients and healthcare providers ensured data saturation and provided a variety of critical perspectives.
- Analyst triangulation corroborated data analysis and strengthened the credibility of the study.
- The accuracy of recall of inpatients interviewed at home (i.e. following hospital discharge) may have been limited by the delay between study recruitment and subsequent data collection.
- Awareness of the interviewer's context as a public health researcher may have resulted in participants distorting their responses to minimise critical judgement.

For peer review only

1. INTRODUCTION

The increasing burden of chronic, non-communicable diseases (NCDs) such as cardiovascular disease, diabetes and chronic respiratory disease has become a global pandemic that is disproportionately affecting low and middle-income countries (LMICs).[1] This is placing great demand on under-resourced health systems that can only be relieved by employing efficient and integrated approaches to healthcare management. Central to efficiency and integration in healthcare is effective handover communication, which involves the exchange of patient-specific information between healthcare providers (HCPs) and between HCPs and patients/carers.[2]

Between HCPs, information exchange is critical during clinical handovers, which are the points in care where information, responsibility and accountability for patient care are transferred from one HCP to another.[3] Such exchange is vital because safe and effective treatment can only be maintained if all relevant information has been shared and understood.[4] A wealth of research from high-income countries (HICs) has evidenced the association between communicative breakdowns during care transitions and risks to patient safety. Such risks are pervasive throughout many aspects of inpatient care and include delays in diagnosis, medication errors and life-threatening adverse events.[5 6] In particular, one-in-five patients experience adverse events following hospital discharge and research has established a link between such events and deficient handover communication.[7-10] Effective information exchange between HCPs and patients is also vital, as patients can provide valuable information to HCPs involved at various stages of their care pathway.[4] Excellent HCP-patient healthcare communication also empowers patients to become active participants in their healthcare management; this is a key aspect of

For peer review only

patient-centred care, which has been linked to improved patient satisfaction and outcomes.[11]

Despite the established importance of handover communication for health systems functioning and patient safety in HICs, there has been a relative dearth of LMIC-based research on this topic.[12] One recent (2019) study from South Africa found inadequate discharge planning to be a significant contributor to avoidable causes of hospital readmission.[13] Across India, a handful of single-site studies have evaluated and described deficiencies in information exchange during hospital shift-change and discharge.[14-16] The current study forms part of a series completed for a project investigating handover and continuity of care for chronic NCD patients in Kerala and Himachal Pradesh states, India. The first study to have been disseminated focussed on outpatient care, which found issues of sub-optimal recording of information within patient-held medical documents and an absence of formal systems for exchanging information between levels of care.[17]

Given these emerging challenges and the established critical link between deficient handover communication and risks to inpatient safety, the current study was conducted to gain novel insight into the transfer of healthcare information during chronic NCD inpatient care across the same study areas of India. The primary objective was to explore knowledge, attitudes and barriers to handover and healthcare communication during the following points of inpatient care:

- Referral/transfer (i.e. communication between HCPs and between HCPs and patients when referring and/or transferring patients)
- Hospital admission and discharge (i.e. communication between HCPs and patients regarding condition, treatment and/or management during hospital admission and discharge)

For peer review only - http://bmjopen.bmj.com/site/about/guidelines.xhtml

A secondary objective was to explore possible interventions to improve the storage and transfer of critical healthcare information.

2. METHODS

2.1 Overview

We report findings from a qualitative study of handover communication for chronic NCD inpatients in two Indian states. This study was conducted from December 2014 to November 2015 across three hospitals: one rural secondary-care hospital in Himachal Pradesh state, and one peri-urban secondary-care and one urban tertiary-care hospital in Kerala state. These settings were selected to capture a range of hospital types within different geographical settings. We selected public rather than private facilities as this is where a large proportion of socio-economically vulnerable patients access healthcare. See supplementary files “S1” and “S2” for further information regarding the Indian healthcare system and study settings.

2.2 Participant recruitment

2.2.1 Patients

Inpatients were recruited opportunistically from hospitals by trained research assistants (n=6).[18] Purposive sampling was used to identify individuals who met the following inclusion criteria: adults (18 years+),[19] admitted to hospital within 24 hours of a researcher first meeting them, due to complications from one of the following

For peer review only - http://bmjopen.bmj.com/site/about/guidelines.xhtml

chronic NCDs: cardiovascular disease, chronic respiratory disease, diabetes mellitus, or hypertension. The identification process took place via researchers approaching ward nurses and asking them about patient demographic and admission details; patients were excluded if judged too unwell to participate by ward nurses. Patients who met the inclusion criteria were provided with verbal and documented study information. Written consent was obtained from literate patients. For illiterate patients, oral consent was obtained along with a thumbprint and signature from a literate witness (i.e. family member/carer) in line with World Health Organisation guidelines.[20] Inpatients were recruited until theoretical saturation was achieved;[21] A total of 20 inpatients participated.

2.2.2 Healthcare Professionals

Healthcare professionals (HCPs) were recruited from study hospitals by trained research assistants (n=6). Due to the busy nature of the study settings, opportunistic sampling was used to recruit as many HCPs as possible with a range of roles and experience.[18] During recruitment, if HCPs stated they were too busy to answer questions they were marked as “unavailable” and not approached again that day - this did not exclude them from participating at another time. HCPs were also recruited until theoretical saturation was achieved;[21] A total of 21 HCPs participated.

2.3 Sample Size

As well as saturation being reached for both participant groups independently, the resulting sample size of 41 participants for this study was in accordance with

For peer review only - http://bmjopen.bmj.com/site/about/guidelines.xhtml

Baker and Edwards' review of sample sizes utilised in qualitative literature, indicating it was sufficient for achieving overall data saturation.[22]

2.4 Data collection

The inpatient data analysed for this study is independent from the outpatient study and was collected using separate topic guides. Regarding HCP data, this study involves secondary analysis of HCP interviews (n=17) included in the outpatient study from participants who were also involved in inpatient care. A small number of additional interviews with HCPs solely involved in inpatient care (n=4) have also been analysed in this study. All HCP interviews in the India handover project were conducted within the same study period and used the same topic guide (as most HCPs in the study areas worked with both outpatients and inpatients on a daily basis).

All interview data was collected entirely by the lead Indian researcher (SJ – an experienced public health researcher) who was familiar with, but not local to, all study areas and fluent in all local Indian dialects and English. Full consideration was given prior to and throughout data collection to ensure that SJ was aware of the potential limitations of working with participants from culturally and linguistically diverse backgrounds. SJ was not involved in patient treatment or previously known to HCPs.

The majority (n=16) of patient interviews took place in study hospitals. Due to a lack of private spaces, they were conducted on inpatient wards in as quiet and private a manner as possible. All participants consented to this and it was ensured that HCPs were not present during inpatient interviews. In addition, a small number

For peer review only - http://bmjopen.bmj.com/site/about/guidelines.xhtml

of patient interviews (n=4) took place in patients' homes either five weeks (n=2) or four months (n=2) following hospital discharge, as this was more convenient for them (i.e. during recruitment they were in the process of being discharged and leaving hospital). The specific follow-up times coincided with community visits being completed for another quantitative study within the India handover project, which the four patients were also participating in. All HCP interviews took place in hospital offices. Interviews with patients and HCPs were conducted in either Hindi, English, Malayalam or a mixture depending on interviewee preference and audio recorded using a digital Dictaphone.

Data collection took place in two stages. In the first stage (December 2014–October 2015), pre-prepared topic guides were used to guide interviews (see supplementary file "S3"). These were developed using relevant handover literature and local knowledge of health systems functioning within the study areas. They were also piloted over three rounds prior to commencement of data collection to ensure they were clear as well as culturally and contextually appropriate. Patient topic guides included open-ended questions focussing on healthcare utilisation, experiences and attitudes of healthcare visits and information exchange. The HCP topic guides differed slightly to capture information on health systems policies and/or practices; it also included questions regarding handover training and potential interventions for improving practices.

Following the first stage of data collection, on the 11th of October 2015, a handover expert's meeting took place in Delhi, India to present preliminary findings and discuss possible interventions. Researchers from the University of Birmingham and University of Warwick (UK) facilitated the presentation of results and group discussions at the meeting. Representatives (n=27) from the following international,

For peer review only - http://bmjopen.bmj.com/site/about/guidelines.xhtml

Indian national and state-level organisations participated: The World Health Organisation; The World Bank; ACCESS Health International; The Ministry of Health and Family Welfare; The Public Health Foundation of India; The National Centre for Disease Control; The Centre for Chronic Disease Control; The National Health System Resource Centre; The All India Institute of Medical Sciences; Aga Khan Health Services; AMRITA Institute of Medical Sciences and Fortis Hospitals. During discussions, a consensus was reached that patient-held booklets were likely to be an acceptable and sustainable intervention to improve information exchange. This was based on the international success of similar patient-held records used in maternal healthcare around the world.[23-27] It also took into account the delays in developing universal electronic information systems and the fact that such systems will not necessarily address the quality of communication between HCPs and patients. Overall, it was opted as the most pragmatic, cost-effective intervention and multiple experts felt that booklets could also improve patient self-management if they contained disease-specific advice.

Therefore, following the meeting the second stage of qualitative data collection (October–November 2015) commenced. Topic guides were updated to include questions regarding the utility of patient-held booklets (see supplementary file “S4”). In addition, if participants stated they had limited time then researchers interviewed them using a shortened topic guide containing targeted questions on patient-held booklets (see supplementary file “S4”).

2.5 Data Analysis

For peer review only - http://bmjopen.bmj.com/site/about/guidelines.xhtml

All audio recordings of interviews were transcribed verbatim and, if necessary, translated into English by SJ. All translations were crosschecked for accuracy by a qualitative expert and co-author in India (SG), who was also familiar with the local context and fluent in all languages utilised during interviews. Following this, all transcripts were sent to the lead UK researcher (CH – public health PhD student) for analysis. CH became familiar with all study sites prior to analysis during research-related visits that were facilitated by the Public Health Foundation of India and the Ministry of Health and Family Welfare in Kerala; visits took place after participation in the handover expert's meeting in Delhi.

Data was analysed using the Framework Method,[28] as this is the method most commonly used for semi-structured interview transcripts. An inductive thematic approach to analysis utilised in Grounded Theory was employed,[29 30] which focused on analysing interviews in their entirety and identifying concepts relevant to handover and healthcare communication during inpatient care that emerged from interviews. Analysis occurred through the following stages central to the Framework Method: transcription, familiarisation, coding, charting, and interpretation. Over a one-month period, familiarisation with the data took place via slow reading of transcripts and CH consulted with SJ to gain a clear understanding of interview contexts. Once this was complete, coding began and two transcripts were chosen at random from each batch of interviews (i.e. 2 inpatients and 2 HCP transcripts) for independent coding by an additional UK analyst (SG – professor of medical sociology with expertise in cross-cultural research) for analyst triangulation.[31] Inpatient and HCP transcripts were coded separately in order to be able to assess similarities and differences between participant groups - Inpatient transcripts were coded first. The coding process involved further familiarisation with the data, followed by open coding where certain

For peer review only

transcript content was highlighted and allocated descriptive labels (codes) to interpret the phenomena identified in the text. The development of codes and themes was entirely data-led and analysed manually.[32]

Microsoft Excel was used to organise participant codes. CH created initial categories by clustering similar codes developed from the two randomly selected inpatient and HCP transcripts. CH and the additional analyst (SG) then met to discuss their analyses. As both had produced similar codes and concepts, the categories that were created were mutually agreed upon. CH then continued with category development until all transcripts had been coded and inserted onto the spreadsheet. Following analysis of 20 inpatient and 26 HCP transcripts, no new categories had been produced. This served as confirmation that data saturation had been met.[21]

Following coding, categories were grouped into subcategories and linked to produce themes. Then, via the process of charting,[28 32] themes for each participant group were used to create a framework matrix into which participants' quotes were inserted, corresponding to their representative subcategory. This provided a visual representation of themes, which facilitated the mapping and interpretation of the data. After completing separate analysis of patient and HCP data, results of both participant groups were compared to assess similarities and differences between their reports of knowledge, attitudes and barriers to handover and healthcare communication. A Venn diagram was used to summarise the separate and overlapping content, which was linked to sub-categories from original themes.

2.7 Patient and public involvement

For peer review only - http://bmjopen.bmj.com/site/about/guidelines.xhtml

Patients and the public were not involved in the initial design of this study. Patients and carers were first involved during the pilot phase prior to formal data collection, where the topic guides, consent and information sheets were piloted over three rounds. During this time, they were consulted and given the opportunity to provide feedback in order to ensure the study materials were comprehensible and culturally and contextually appropriate. Patients and the public were not involved in any other aspect of the study recruitment or conduct, but findings have been disseminated publicly via an experts meeting (including professionals working with patient groups) and open access web pages.

3. RESULTS

3.1 Inpatient characteristics

20 male (n=10) and female (n=10) inpatients aged between 25 and 71 were interviewed. Participants' background characteristics were varied (Table 1). Inpatients completed interviews in English (n=11), Hindi (n=4), Malayalam (n=4) and a mixture of Hindi and English (n=1).

For peer review only - http://bmjopen.bmj.com/site/about/guidelines.xhtml

Table 1. Inpatient characteristics

Characteristics	1	2	3	4	5	6	7	8	9	10	11	12	13	14	15	16	17	18	19	20	N (%)
**Age**	65	45	70	58	71	56	57	70	55	25	72	50	55	69	70	50	70	70	70	70	25-71
**Sex**																					
Male	✓	✓	✓	✓	✓				✓	✓	✓	✓				✓					10 (50)
Female						✓	✓	✓					✓	✓	✓		✓	✓	✓	✓	10 (50)
**Literacy**																					
Illiterate								✓					✓		✓	✓	✓	✓	✓	✓	8 (40)
Literate	✓	✓	✓	✓	✓	✓	✓		✓	✓	✓	✓		✓							12 (60)
**Education Level**																					
None/minimal primary school-level								✓				✓	✓		✓	✓	✓	✓	✓	✓	9 (45)
Completed lower primary school					✓		✓														2 (10)
Completed upper primary school						✓															1 (5)
Completed secondary school									✓												1 (5)
Completed higher vocational studies																					0 (0)
University graduate (or above)	✓			✓						✓	✓										4 (20)
No data		✓	✓											✓							3 (15)
**Employment Status**																					
Employed		✓		✓	✓				✓			✓	✓	✓		✓					8 (40)
Unemployed						✓	✓	✓							✓		✓	✓	✓	✓	8 (40)
Student										✓											1 (5)
Retired	✓		✓								✓										3 (15)
**Chronic NCD(s) (related to admission)**																					
Chronic Respiratory Disease			✓	✓		✓		✓					✓			✓					6 (30)
Diabetes	✓													✓	✓	✓	✓	✓	✓	✓	8 (40)
Hypertension									✓		✓	✓				✓	✓	✓			6 (30)
Cardiovascular Disease (other than Hypertension alone)	✓	✓			✓		✓	✓	✓	✓	✓	✓									9 (45)
**Language(s) used during Interview**																					
English (only)	✓	✓	✓	✓										✓	✓	✓	✓	✓	✓	✓	11 (55)
Hindi (only)						✓	✓	✓	✓												4 (20)
Malayalam (only)										✓	✓	✓	✓								4 (20)
English & Hindi (mixture)					✓																1 (5)

For peer review only

For peer review only - http://bmjopen.bmj.com/site/about/guidelines.xhtml

3.2 Healthcare Professional characteristics

21 male (n=15) and female (n=6) HCPs aged between 22 and 55 were interviewed. HCP roles included doctors (n=17), nurses (n=2), pharmacists (n=1) and medical records officers (n=1). HCP qualifications and experience were varied (Table 2). HCPs completed interviews in English (n=15), Hindi (n=2), Malayalam (n=2) and a mixture of Hindi and English (n=2).

For peer review only

Table 2. Healthcare professional characteristics

Characteristics	1	2	3	4	5	6	7	8	9	11	14	15	18	19	20	21	22	23	24	25	26	N (%)
**Age**	44	24	33	25	23	39	44	35	52	50	50	43	50	40	46	55	22	35	35	45	35	22-55
**Sex**																						
Male	✓		✓	✓	✓		✓		✓	✓	✓	✓	✓	✓	✓	✓	✓			✓		15 (71.4)
Female		✓				✓		✓										✓	✓		✓	6 (28.6)
**Qualification/s***																						
Doctor of Medicine (MD)	✓		✓				✓	✓	✓	✓	✓	✓	✓	✓	✓	✓	✓					13 (61.9)
Master of Public Health (MPH)						✓																1 (4.8)
Bachelor of Medicine (MBBS)	✓	✓	✓	✓	✓	✓	✓	✓	✓	✓	✓	✓	✓	✓	✓	✓	✓					17 (81.0)
BSc Nursing																		✓			✓	2 (9.5)
BSc Pharmacy																			✓			1 (4.8)
Graduate (i.e. non-medical degree)																				✓		1 (4.8)
**Official position**																						
Medical Superintendent						✓																1 (4.8)
Chief Medical Officer									✓													1 (4.8)
Medical Officer												✓										1 (4.8)
Consultant	✓		✓				✓	✓					✓	✓	✓	✓	✓					9 (42.9)
Surgeon		✓								✓												2 (9.5)
General Medicine											✓											1 (4.8)
Intern Doctor				✓	✓																	2 (9.5)
Ward Nurse																		✓			✓	2 (9.5)
Pharmacist																			✓			1 (4.8)
Medical Records Officer																				✓		1 (4.8)
**Years of experience in position**																						
<1		✓			✓																	2 (9.5)
1 – 3				✓																		1 (4.8)
4 – 6						✓		✓										✓		✓	✓	5 (23.8)
7 – 10			✓				✓												✓			3 (14.3)
>10	✓								✓	✓	✓	✓	✓	✓	✓	✓	✓					10 (47.6)
**Place of work**																						
General Hospital		✓	✓	✓	✓									✓		✓	✓	✓	✓			9 (42.9)
Regional Hospital	✓								✓	✓	✓		✓							✓	✓	7 (33.3)
Taluk Hospital						✓	✓	✓				✓			✓							5 (23.8)
**Language(s) used during interview**																						
English (only)		✓	✓	✓	✓	✓	✓	✓	✓	✓	✓	✓		✓	✓	✓	✓					15 (71.4)
Hindi (only)																				✓	✓	2 (9.5)
Malayalam (only)																		✓	✓			2 (9.5)
English & Hindi (mixture)	✓												✓									2 (9.5)

* HCPs could select more than one answer for this question

For peer review only

3.3 Charted Data

During analysis of patient and HCP data, three themes (with subcategories) emerged for each participant group. Patient themes were 1.Public healthcare service characteristics, 2. HCP to patient communication and 3.Attitudes regarding medical information (Table 3). HCP themes were 1.System factors, 2.Information exchange practices and 3.Quality improvement strategies (Table 4).

Following separate analysis of patient and HCP data, the results of both participant groups were compared to assess similarities and differences between their reports of knowledge, attitudes and barriers to handover communication. The results of this comparison are displayed in Figure 1 (see supplementary file "S5").The similarities will be described first, followed by the differences. To ensure confidentiality, numerical pseudonyms have been used when presenting quotes.

For peer review only

For peer review only - http://bmjopen.bmj.com/site/about/guidelines.xhtml

Table 3. Summary of charted data for inpatients (IPs)

	Public healthcare service characteristics		HCP to patient communication			Attitudes regarding medical information		
IP	Large patient loads (overcrowding)	Deficient primary care services	Verbal healthcare information (during admission)	Referral information	Impoliteness/ impatience	Transportation of medical documents	Patient-held booklet intervention	Dissatisfaction with lifestyle advice
1						✓		
2			✓					
3	✓		✓		✓	✓		
4			✓			✓		
5		✓	✓	✓	✓			
6				✓		✓		
7	✓	✓		✓		✓		
8				✓				
9			✓					
10			✓					
11	✓		✓			✓		
12			✓	✓		✓		
13			✓			✓		
14		✓	✓				✓	✓
15		✓	✓		✓		✓	✓
16		✓	✓			✓	✓	
17			✓			✓	✓	✓
18		✓	✓			✓	✓	
19			✓			✓	✓	
20			✓				✓	✓

For peer review only - http://bmjopen.bmj.com/site/about/guidelines.xhtml

Table 4. Summary of charted data for healthcare professionals (HCPs)

HCP	System factors			Information exchange practices					Quality improvement strategies			
	Time & resource constraints	Absence of handover communication training	Absence of structured formats for information exchange between HCPs	Hospital record keeping	Ad-hoc phone calls	Patient-held medical documents	Discharge instructions	Hierarchical transfer of responsibility	Increase resource provision	Introduce formal referral systems	Implement "e-health" systems	Patient-held booklet intervention
1			✓			✓	✓		✓			
2			✓			✓	✓					
3						✓			✓	✓	✓	
4	✓		✓			✓	✓					
5			✓	✓		✓						
6	✓		✓						✓		✓	
7	✓		✓		✓							
8	✓				✓							
9									✓			
11	✓	✓	✓		✓		✓					
14	✓	✓	✓						✓	✓		
15				✓		✓			✓		✓	
18	✓										✓	✓
19	✓											✓
20	✓					✓						✓
21												✓
22	✓							✓				✓
23				✓		✓	✓	✓				
24	✓			✓								
25				✓								
26	✓							✓				

For peer review only

For peer review only - http://bmjopen.bmj.com/site/about/guidelines.xhtml

3.4 Overlapping content

3.4.1 Public healthcare constraints

During interviews, a number of patients reported that they chose to visit public hospitals because of the better availability of healthcare staff compared to local healthcare facilities, such as smaller hospitals and primary/community health centres:

> "*We have very limited time, we did go to local hospital but doctors are not there. So if we get time we will come here rather than going to a hospital where there are no doctors. (IP 15)"*

However, multiple patients also reported that government hospitals were often crowded with high daily patient loads:

> *"There is so much crowd there you can't ask or hear anything there… so many people are there now, you cannot do anything. (IP 11)"*

The human resource issues at government primary and community healthcare facilities were also mentioned by HCPs:

> *"It will be useful if availability of doctors is ensured at the peripheral institutions around the clock. At times it is not there. (DOC 1)"*

Additionally, in our study settings most hospital doctors worked in both outpatient clinics and inpatient wards on a daily basis. Many doctors expressed concerns of time pressures due to the large patient volumes seen at hospital outpatient clinics and the subsequent lack of time they had to attend to all patients:

For peer review only - http://bmjopen.bmj.com/site/about/guidelines.xhtml

"We can hardly spend five minutes with each patient, seeing the crowd you will just want to finish everyone soon. (DOC 7)"

Some doctors also reported that human and medical resource constraints across public healthcare facilities were hindering quality of care:

"[It's] not [about] motivation, [it's about] resource limitation. It's not humanly possible to see people every day for seven days. Quality definitely gets compromised. (DOC 3)"

3.4.2 Referral communication

A number of patients who recalled being referred from a previous healthcare facility to the hospital reported that they were not provided with any referral information:

"No, they didn't give any parchi [papers]. We were getting medicines right only that is with us. (IP 8)"

HCPs also discussed referral communication. Doctors explained that there were no structured processes to follow for information exchange during referrals:

"Yeah there is no proper way of doing it… inpatients sometimes we have to [refer] but as I told you we never had a structured format. (DOC 14)"

However, despite a lack of structured systems, some doctors described making ad-hoc calls to ensure that some information was transferred when referring a patient:

"Sometimes I call the doctor to tell them that so and so is coming. Please do the needful. If I know the patient or doctor. (DOC 11)"

For peer review only - http://bmjopen.bmj.com/site/about/guidelines.xhtml

3.4.3 Patient-held medical information

At the point of hospital admission, patient-held notes and/or medical records can facilitate optimal care by providing HCPs with key patient-specific information. When asked about whether they brought medical papers to the hospital, most patients reported that they regularly stored and transported papers to HCP visits; these included referral notes, prescription cards, test results and other records from inpatient/outpatient/primary care:

> *“"Yeah we have always kept everything safely. [Shows researcher a bag with all sort of papers like reports, lab tests, etc.] (IP 3)”*

Doctors also talked about patient-held medical information during interviews. For example, some doctors reported that patients regularly kept and transported their medical records:

> *“Almost everyone comes with medical reports. (DOC 11)”*

However, other doctors described that, in their experience, the availability of patient-held records was less consistent and that this could negatively impact continuity of care:

> *“Some of them do bring investigations and all others don’t bring much and we have to work out what happened from the start. (DOC 3)”*

3.4.4 Healthcare management communication

When asked about verbal HCP communication, many patients reported that during admission and/or discharge a HCP had provided them with some basic verbal

For peer review only - http://bmjopen.bmj.com/site/about/guidelines.xhtml

healthcare management information (i.e. medication, treatment, lifestyle and/or follow-up requirements). However, the quantity of information received appeared to vary notably between patients. For example, some recalled being given detailed instructions:

> *"Doctor says everything. I was given medicines and now they asked me to take injections also. Doctor is saying I am not controlling my sugar. The nurse taught me how to take injection. (IP 19)"*

Conversely, others appeared to receive relatively limited information and one carer reported having to seek healthcare advice from alternative sources:

> *"Doctors don't explain everything. We speak to our friends and get details from them. (Carer - IP 16)"*

HCPs also discussed their healthcare communication practices with inpatients. Whilst talking about discharge, a nurse described the usual amount of time taken to explain information to each patient:

> *"Usually we take 20-25 minutes to instruct the patients. If the patients understand then it can be even faster. (NUR 1)"*

Doctors reported that they provided patients with documented information on discharge cards and verbally advised inpatients to return to their local HCP/healthcare institution during the discharge consultation:

> *"We give them a discharge card. Discharge card is there we have written and then we refer them to the local hospital or where they come from. (DOC 15)"*

For peer review only - http://bmjopen.bmj.com/site/about/guidelines.xhtml

3.4.5 Booklet intervention

During interviews that took place after the handover expert meeting, patients were asked for their opinion regarding the utility of a patient-held booklet where records could be stored and transported to HCP visits. Most appeared to think that it might be effective and could help with self-management, including those who were illiterate:

> *"Yeah, sometimes we don't know what to do so it would be good if some paper is there to help us. We can't read it ourselves but our son or daughter-in-law can help us. (IP 17)"*

HCPs were also asked for their opinions regarding the booklet intervention. Many generally felt it could be useful, but various conditions and/or reservations were also expressed. For example, doctors felt that the success of the booklet would rely on patient attitudes:

> *"That will depend on the patients, if they maintain that and bring it every time. For us there is no change, we write our observations in paper or notebook, doesn't matter… Might be helpful. (DOC 22)"*

Related to this, one doctor felt that to see the most benefit, patients needed to be regularly instructed to keep and transport their medical documents:

> *"We write the communication but the patients don't keep them proper. I think we have to tell the patients to keep the letters and papers. (DOC 4)"*

For peer review only - http://bmjopen.bmj.com/site/about/guidelines.xhtml

3.5 Content unique to patients

3.5.1 Attitudes regarding HCP communication during admission

A few patients recalled receiving some unfavourable/impolite treatment from healthcare staff during their hospital admission:

> *"The doctor's don't speak much. They explain but get angry if you don't understand them. (IP 3)"*

In addition, some patients expressed dissatisfaction with the lifestyle advice provided. In particular, patients of lower socio-economic status felt that nutritional instructions were not suitable for them due to their time and financial constraints:

> *"We are daily labourers we can't follow all the instructions… We can't follow that, we are poor we do hard work and we just can't concentrate on eating. Whatever is there we just eat. (IP 15)"*

3.6 Content unique to HCPs

3.6.1 Institutional/systemic factors

Some doctors displayed good knowledge of the key information should be transferred during patient referrals/transfers and/or hospital discharge.

> *"To another hospital, yeah first we have to write what are the main complaints of patients presenting illness and write about the past history, then we will write about what all investigations we have done here 'til the day of transfer, then what is the condition of the patient we are discharging, why we are*

For peer review only - http://bmjopen.bmj.com/site/about/guidelines.xhtml

discharging (and) any investigations, major investigations, to be done. (DOC 2)"

However, when asked about training opportunities numerous doctors mentioned that they had not received any formal handover training. Some recalled that this type of training was not provided at medical school:

"I think it was not there in medical curriculum. (IP 1)"

Others reported that training was not provided in their workplace/s and instead they learned on the job:

"We are sent to the wards, we see what our seniors do and we do that's all. We have to develop our communication skills ourselves no formal training is there. (DOC 14)"

When asked about hospital record keeping, a medical records officer stated that inpatient records are stored in hospitals following patient discharge for up to ten years. However, the same officer also indicated that these paper-based records are not easily accessible:

"Definitely I can locate any record but it might take some time to locate them. (MRO 1)"

3.6.2 Organisational culture

Based on reports from both doctors and nurses, it appeared as though some hierarchical transfer of responsibility for documented handover communication took place in hospitals. For example, a senior doctor mentioned that they instructed medical interns to write notes for them when their patient load was high:

For peer review only

"We do write in the papers, whether it's discharge card or outpatient sheets. When patient load is high, then we tell our interns to do it for us, we check that and then sign. (DOC 22)"

3.6.3 Requirements for improving information exchange

During interviews, HCPs were asked for their thoughts on requirements to improve information exchange between HCPs and between HCPs and patients. Numerous doctors felt that there needed to be a notable increase in public healthcare resource provision:

"Infrastructure is very small but the outpatient department is ten times more than it can manage, so more posts should be created… We have to increase the manpower and also our materials. (DOC 15)"

In addition, doctors discussed the idea of introducing structured referral documents and systems to improve referral communication:

"You can supply people with [referral] forms and make it mandatory that residents have to maintain a register. In that case they will maintain the register. (DOC 3)"

Whilst discussing current information systems one doctor in Kerala reported that an application had been made for a near-future transition to computerised healthcare information systems. This appeared to be a state-wide plan for public healthcare facilities:

"We have submitted a proposal for paperless computerisation system for doctors, so I think state-wide they are planning to do that. (DOC 6)"

For peer review only - http://bmjopen.bmj.com/site/about/guidelines.xhtml

4. DISCUSSION

4.1 Main findings

This study presents qualitative data on patient and HCP knowledge, attitudes and barriers to handover and healthcare communication during public hospital inpatient care in Kerala and Himachal Pradesh states, India. The main findings are that verbal and documented information exchange between HCPs and between HCPs and patients is often suboptimal during referrals/transfers, hospital admission and discharge, with a lack of structured systems and HCP education in place to ensure sufficient continuity of care. Whilst unique themes emerged for both patients and HCPs, comparison of the results showed that there was also a notable amount of overlapping content. The results have highlighted the multifaceted nature of handover and healthcare communication during inpatient care in India. With regard to public health, the findings have also elucidated a number of key areas to address to improve the continuity and safety of chronic NCD patient care.

Some of the results from the current study reflect and reinforce findings from previous research focussing on outpatient care in the same study areas of India.[33] In particular, during interviews in both studies, patients and HCPs recognised the resource constraints affecting public healthcare. The main issue reported was deficient primary healthcare services, which is in line with well-established findings of limited primary care infrastructure across India and numerous LMICs.[34] In our study settings, under-resourced primary care resulted in many patients preferring to visit hospitals as the first point of care. Subsequently, large patient loads were seen in both outpatient and inpatient departments, which limited HCP consultation times. Other key areas of discussion in the currently study reflected in the outpatient

For peer review only - http://bmjopen.bmj.com/site/about/guidelines.xhtml

findings were inconsistent transportation of patient-held medical documents and views regarding the utility of patient-held record booklets. Whilst more inpatients than outpatients reported that they regularly transported records to HCP visits, doctors recalled seeing many who did not bring information to hospital. This was problematic as if patients did not bring their records then doctors had to gather details from scratch, potentially compromising continuity of care. When asked about the possible utility of introducing patient-held record booklets to store and transport documents, inpatients had similar views to outpatients which were generally positive, but also felt that the inclusion of self-management information would be beneficial. Doctors in the current study expressed a wider variety of views, but broadly thought that booklets could be useful if patients had positive attitudes towards their maintenance and use.

Regarding referral communication, the current study also highlighted similar issues of deficient information exchange observed in the previous outpatient study.[33] For example, reports from HCPs and inpatients revealed that that documented information was often provided in the form of minimal, hand-written notes on papers provided for other purposes (e.g. prescription cards). These findings reflect results from other LMIC studies that have evidenced the exchange of poor-quality referral documents.[35-37] However, the current study also evidenced inpatient reports of not being provided with any documented information during referrals. Further, whilst a small number of inpatient HCPs in the current study explained that they called HCPs to discuss a referral case, it was revealed that this was dependent on how well they knew the patient and/or HCP. Such findings indicate that there are further inconsistencies in referral communication practices than previously described. Overall, such deficits are unsurprising given that multiple HCPs in both the current and previous outpatient study reported an absence of structured systems and

For peer review only - http://bmjopen.bmj.com/site/about/guidelines.xhtml

education provided for handover communication. These findings are also in line with the few previous descriptions from India of an absence of training and protocols for handover practices.[14-16]

In addition to similarities found with previous research, the current study has elucidated numerous novel insights regarding handover and healthcare communication during critical points in inpatient care, which were previously unexplored in the study areas of India. Regarding inpatient medical record keeping, a records officer reported that hospital records were not easily accessible and rarely retrieved. This lack of available and accurate medical information, paired with inconsistent transportation of patient-held records, carries notable risks for patient safety. This is because without key patient background and/or treatment details, critical oversights can be made that result in adverse events.[4 5 7] Additionally, there were notable variations in patient reports of the provision of healthcare management information during hospital admission and discharge; whilst some patients reported being given clear self-care instructions, others stated that they sought information from external sources due to the lack of detail provided by hospital HCPs. It appears that the time pressures experienced by HCPs and were a significant contributory factor to inconsistencies in HCP to patient communication, particularly at the point of discharge. During interviews, senior doctors reported often being so busy with high patient loads that they would pass the duty of writing discharge notes to interns or nurses. Additionally, it seemed that more time was spent on verbal discharge communication, with a nurse reporting that they typically took approximately twenty minutes per patient to explain discharge instructions. Such practices may be compromising the retention of key healthcare information, as global literature suggests that patients can struggle to absorb verbal details provided during

For peer review only - http://bmjopen.bmj.com/site/about/guidelines.xhtml

consultations.[38] The potential implications of these are findings are significant, given the associations that have been found between deficient discharge communication and an increased likelihood of adverse events.[7-10]

Other key issues affecting handover and healthcare communication during admission and discharge were mentioned solely by each participant group. For patients, some recalled receiving impolite treatment from hospital doctors during admission. Additionally, a small number of patients were dissatisfied with the take-home nutritional advice provided, as they felt it failed to take into account their socio-economic deprivation. These results may be explained by the reported lack of communication training in medical education, as well as a historic tendency for paternalistic physician conduct in India.[39] In other areas of India and Asia, research on HCP-patient communication has also evidenced asymmetric power balances and patient dissatisfaction during patient consultations.[40] Such findings reveal the need for more patient-centred communication, particularly for poorer patients that make up a significant proportion of public healthcare users. As for HCPs, during interviews many doctors recognised the need for an increase in public healthcare resource provision, as well as structured systems for information exchange. Some also discussed the promise of implementing “e-health” systems, with a doctor in Kerala reporting that public healthcare facilities across the state will be transitioning to computerised systems. Whilst our colleagues from Kerala report that this development is in its early stages, it holds potential as similar systems have advanced the accessibility and quality of healthcare information across the globe.[41 42]

For peer review only - http://bmjopen.bmj.com/site/about/guidelines.xhtml

4.2 Strengths and limitations

As far as the authors are aware, this is the first study to qualitatively explore factors affecting multiple areas of handover communication for chronic disease inpatients in India. The use of multiple sites and qualitative methodology has revealed a number of key issues that are supported among the HIC and emerging LMIC literature, suggesting likely transferability to other LMIC settings. Additionally, interviews with both patients and HCPs have provided multiple key perspectives regarding critical areas of handover communication for inpatient care. The number of interviews conducted helped to ensure data saturation for both participant groups and study credibility was strengthened via use of multi-analyst triangulation.[31]

The lack of documented inclusion/exclusion rates for participation is a study limitation, as this could not be recorded. In addition, the accuracy of recall of the minority of patients interviewed at home may have been limited by the delay between recruitment and data collection. Recruitment challenges meant that patient participants were predominantly older (i.e. 45yrs+), which limited exploration of younger patient experience; this was, however, largely unsurprising given the study exclusively recruited inpatients with chronic NCDs. The cross-cultural nature of this research may have also resulted in constraints during data collection and analysis, as in-group bias could have affected participants' willingness to openly converse with a non-local researcher.[43] Social desirability bias from the use of individual interviews and participant's awareness that the interviewer was a public health professional may have also affected truthfulness of the data.[44] Despite these challenges, the recurrence of themes indicating data saturation and the finding that our results are supported by existing literature suggests that they had minimal impact.

For peer review only - http://bmjopen.bmj.com/site/about/guidelines.xhtml

4.3 Conclusions and next steps

This study has found that handover communication for chronic NCD inpatients during referrals/transfers, hospital admission and discharge is often fragmented. The critical barriers appear to be an absence of structured information exchange systems and HCP education. There is also a growing recognition of the need for the government to strengthen primary healthcare infrastructure in line with the declaration of Alma Alta.[45] This will greatly assist in increasing accessibility of care and subsequently reducing pressure on hospital services. It will also be required to address the United Nations sustainable development goals regarding universal health coverage and reducing premature deaths from NCDs.[46] In addition, the implementation of structured documentation, systems and training is urgently required to manage critical care transitions such as referral and discharge. Research from both HIC and LMIC settings has proven that such interventions can improve continuity and safety of care.[4 16 35 47] Regarding future steps, during HCP interview it was reported that public healthcare facilities in Kerala will be transitioning to computerised “e-health” information systems the Indian government has since pledged to digitise all public healthcare information systems via an “Integrated Health Information Platform”.[48] Whilst such developments hold promise and are making progress in Kerala, they remain in their initial stages in many states and face numerous technological challenges. Additionally, they are not likely to target issues regarding HCP to patient communication, patient access to healthcare information and information exchange between public and private providers.

For peer review only - http://bmjopen.bmj.com/site/about/guidelines.xhtml

Therefore, a mixed-methods pilot study exploring the design and implementation of patient-held record booklets is suggested. This could ameliorate some of the current issues by incorporating disease-specific and structured documents, which have been shown to improve recording of clinical information,[47 49 50] and providing a means of organising records in a logical and accessible way. The patient-held nature of this strategy would increase patient access to key healthcare information, which may improve self-management. Given the unstructured, predominantly paper-based systems utilised across the study sites, this is an area for development that has been welcomed by Indian national and international experts, as well as patients and HCPs in our study areas. There have also been multiple international successes of improved continuity of care via utilisation of similar patient-held/home-based records in outpatient care and maternal and child health.[23-27 51] In order to maximise booklet utilisation, it would be necessary to address the issues surrounding patient retention and understanding of the importance of medical documents. Initial key steps could be to involve both patients and HCPs in the design process and accompany the introduction of booklets with relevant promotion, training and incentives.

Finally, given the rising burden of NCDs across LMICs, this research is timely and crucial for effective health systems development. It is important that further LMIC research is conducted to explore critical factors affecting quality, continuity and safety of care and to develop sustainable and cost-effective interventions.

For peer review only - http://bmjopen.bmj.com/site/about/guidelines.xhtml

ACKNOWLEDGEMENTS

This research was supported by the National Institute for Health Research (NIHR) Collaboration for Leadership in Applied Health Research and Care West Midlands (NIHR CLAHRC WM). We would like to extend our thanks to all patients, healthcare staff and researchers who kindly took the time to participate in this project. We are also indebted and give thanks to the participating hospitals and community healthcare facilities. Finally, we are very grateful to the Directors of Health from both Himachal Pradesh and Kerala States, India, for their assistance in facilitating this project. Without their support this research would not have been possible.

AUTHOR CONTRIBUTIONS

In order of the authors list: CH: Data curation, formal analysis, visualisation, writing (both original draft and final review and editing). SJ: Investigation, data curation, project administration, writing (review and editing). JP: Conceptualisation, funding acquisition, project administration, supervision, writing (review and editing). SS: Funding acquisition, project administration, supervision, writing (review and editing). SG(oenka): Funding acquisition, projection administration, supervision, writing (review and editing). PD: Conceptualisation, funding acquisition, project administration, supervision, writing (review and editing). PG: Conceptualisation, data curation, funding acquisition, writing (review and editing). SG(reenfield): Funding acquisition, formal analysis, writing (review an editing). RL: Conceptualisation,

For peer review only - http://bmjopen.bmj.com/site/about/guidelines.xhtml

funding acquisition, writing (review and editing). SMH: Conceptualisation, funding acquisition, investigation, methodology, project administration, supervision, writing (review and editing).

FUNDING STATEMENT

This research was joint-funded by the Department of International Development, the Economic and Social Research Council, the Medical Research Council and the Wellcome Trust - grant number: MR/M00287X/1. The funders had no role in study design, conduct or reporting.

COMPETING INTERESTS STATEMENT

The authors of this manuscript have no competing interests to disclose.

ETHICS APPROVAL

This study was reviewed and approved by the Centre for Chronic Disease Control Independent Ethics Committee, India, and the Amrita Institute of Medical

For peer review only

Sciences Institutional Ethics Committee, India. Data archives will be stored at the University of Birmingham, in accordance with the University's code of practice.

DATA SHARING STATEMENT

Deidentified participant transcript data generated and analysed during the current study are available from the corresponding author on reasonable request.

Contact Details: Dr. Semira Manaseki-Holland, s.manasekiholland@bham.ac.uk.

For peer review only

REFERENCES

1. Islam SMS, Purnat TD, Phuong NTA, et al. Non-Communicable Diseases (NCDs) in developing countries: a symposium report. *Globalization and health* 2014;10(1):81.
2. World Health Organisation. Communication during patient hand-overs. *Patient safety solutions* 2007;1(3):1-4.
3. British Medical Association. Safe handover: safe patients. *Guidance on clinical handover for clinicians and managers London: BMA* 2004;7:141.
4. Merten H, van Galen LS, Wagner C. Safe handover. *Bmj* 2017;359:j4328. doi: https://dx.doi.org/10.1136/bmj.j4328
5. Australian Council for Safety Quality in Health Care. Clinical handover and patient safety: Literature review report. 2005
6. Hesselink G, Schoonhoven L, Barach P, et al. Improving patient handovers from hospital to primary care: a systematic review. *Ann Intern Med* 2012;157(6):417-28. doi: https://dx.doi.org/10.7326/0003-4819-157-6-201209180-00006
7. Forster AJ, Murff HJ, Peterson JF, et al. The incidence and severity of adverse events affecting patients after discharge from the hospital. *Ann Intern Med* 2003;138(3):161-7.
8. Moore C, Wisnivesky J, Williams S, et al. Medical errors related to discontinuity of care from an inpatient to an outpatient setting. *J Gen Intern Med* 2003;18(8):646-51.
9. Li JY, Yong TY, Hakendorf P, et al. Timeliness in discharge summary dissemination is associated with patients' clinical outcomes. *Journal of evaluation in clinical practice* 2013;19(1):76-79.
10. van Walraven C, Seth R, Austin PC, et al. Effect of discharge summary availability during post-discharge visits on hospital readmission. *J Gen Intern Med* 2002;17(3):186-92.
11. Bradley S, Mott S. Adopting a patient-centred approach: an investigation into the introduction of bedside handover to three rural hospitals. *J Clin Nurs* 2014;23(13-14):1927-36. doi: https://dx.doi.org/10.1111/jocn.12403
12. Humphries C MS, Krynicki C, Nandhra K, Novielli N, Amin A, Sheikh A, Saha P, Joseph L, Gummaraju A,Lilford R, Cheng Y F, Manasek-Holland S. Handover communication in low and middle-income countries: A systematic review PROSPERO international prospective register of systematic reviews2019 [Available from: https://www.crd.york.ac.uk/prospero/display_record.php?RecordID=9309.
13. Dreyer R, Viljoen AJ. Evaluation of factors and patterns influencing the 30-day readmission rate at a tertiary-level hospital in a resource-constrained setting in Cape Town, South Africa. *South African Medical Journal Suid-Afrikaanse Tydskrif Vir Geneeskunde* 2019;109(3):164-68.
14. Kumar PJ, V.; Vij, A.; Gupta, S K. . Who is more hands on with hand-offs? A comparative study of clinical handovers among doctors and nurses in a tertiary care center in India. *International Journal of Research Foundation of Hospital & Healthcare Administration* 2015;3(1):33-40.
15. Kumar P, Jithesh V, Vij A, et al. Need for a hands-on approach to hand-offs: A study of nursing handovers in an Indian Neurosciences Center. *Asian journal of neurosurgery* 2016;11(1):54.
16. Rajavelu P, Rajagopalan A. Pre-formatted written discharge summary—a step towards quality assurance in the emergency department. *International journal of emergency medicine* 2008;1(4):321-25.
17. Humphries C, Jaganathan S, Panniyammakal J, et al. Investigating clinical handover and healthcare communication for outpatients with chronic disease in India: A mixed-methods study. *PLoS One* Forthcoming
18. Marshall MN. Sampling for qualitative research. *Fam Pract* 1996;13(6):522-26.
19. Palys T. Purposive Sampling. In: Given LM, ed. The Sage Encylopedia of Qualitative Research Methods. Los Angeles: Sage 2008:697-98.
20. World Health Organistion. Information for Researchers Concerning Informed Decision Making [Internet]. Available from:

For peer review only - http://bmjopen.bmj.com/site/about/guidelines.xhtml

http://www.who.int/rpc/research_ethics/Process_seeking_IF_printing2.pdf accessed 01/05/18.
21. Seale C. Quality in qualitative research. *Qualitative inquiry* 1999;5(4):465-78.
22. Baker SE, Edwards R, Doidge M. How many qualitative interviews is enough?: Expert voices and early career reflections on sampling and cases in qualitative research. 2012
23. Shah PM, Selwyn BJ, Shah K, et al. Evaluation of the home-based maternal record: a WHO collaborative study. *Bull World Health Organ* 1993;71(5):535-48.
24. Osaki K, Hattori T, Kosen S. The role of home-based records in the establishment of a continuum of care for mothers, newborns, and children in Indonesia. *Glob Health Action* 2013;6:1-12. doi: https://dx.doi.org/10.3402/gha.v6i0.20429
25. Turner KE, Fuller S. Patient-held maternal and/or child health records: meeting the information needs of patients and healthcare providers in developing countries? *Online journal of public health informatics* 2011;3(2)
26. Takeuchi J, Sakagami Y, Perez RC. The Mother and Child Health handbook in Japan as a health promotion tool: An overview of its history, contents, use, benefits, and global influence. *Global pediatric health* 2016;3:2333794X16649884.
27. Walton S, Bedford H, Dezateux C. Use of personal child health records in the UK: findings from the millennium cohort study. *Bmj* 2006;332(7536):269-70.
28. Spencer L, Ritchie J. Qualitative data analysis for applied policy research. Analyzing qualitative data: Routledge 2002:187-208.
29. Braun V, Clarke V. Using thematic analysis in psychology. *Qualitative research in psychology* 2006;3(2):77-101.
30. Corbin JM, Strauss A. Grounded theory research: Procedures, canons, and evaluative criteria. *Qualitative sociology* 1990;13(1):3-21.
31. Lincoln YS, Guba EG. Naturalistic inquiry. Beverley Hills: ca: sage, 1985.
32. Gale NK, Heath G, Cameron E, et al. Using the framework method for the analysis of qualitative data in multi-disciplinary health research. *BMC medical research methodology* 2013;13(1):117.
33. Humphries C, Jaganathan S, Panniyammakal J, et al. Investigating clinical handover and healthcare communication for outpatients with chronic disease in India: A mixed-methods study. *PLoS ONE [Electronic Resource]* 2018;13(12):e0207511.
34. Das J, Hammer J. Quality of primary care in low-income countries: facts and economics. *Annu Rev Econ* 2014;6(1):525-53.
35. Janati A, Amini A, Adham D, et al. Assessing the quality of referral letters written by general practitioners: a cross-sectional study in rural Iran. *Cadernos de Saude Publica* 2017;33(2):e00043016.
36. Gyedu A, Baah EG, Boakye G, et al. Quality of referrals for elective surgery at a tertiary care hospital in a developing country: an opportunity for improving timely access to and cost-effectiveness of surgical care. *Int J Surg* 2015;15:74-8. doi: https://dx.doi.org/10.1016/j.ijsu.2015.01.033
37. Osinaike BO, Esezobor CI, Akinsola OJ. Quality of Referral Letters to the Paediatric Department of a Tertiary Hospital in Nigeria. *Nigerian Quarterly Journal of Hospital Medicine* 2013;23(4):273-9.
38. Kessels RP. Patients' memory for medical information. *Journal of the Royal Society of Medicine* 2003;96(5):219-22.
39. Ghooi R, Deshpande S. Patients' rights in India: an ethical perspective. *Indian J Med Ethics* 2012;9(277):e8.
40. Pun J, Chan EA, Wang S, et al. Health professional-patient communication practices in East Asia: An integrative review of an emerging field of research and practice in Hong Kong, South Korea, Japan, Taiwan, and Mainland China. *Patient education and counseling* 2018

For peer review only

For peer review only - http://bmjopen.bmj.com/site/about/guidelines.xhtml

41. Piette JD, Lun KC, Moura LA, Jr., et al. Impacts of e-health on the outcomes of care in low- and middle-income countries: where do we go from here? *Bull World Health Organ* 2012;90(5):365-72. doi: https://dx.doi.org/10.2471/BLT.11.099069
42. Campanella P, Lovato E, Marone C, et al. The impact of electronic health records on healthcare quality: a systematic review and meta-analysis. *Eur J Public Health* 2016;26(1):60-4. doi: https://dx.doi.org/10.1093/eurpub/ckv122
43. Tajfel H. Social psychology of intergroup relations. *Annual review of psychology* 1982;33(1):1-39.
44. Spector P. Social desirability bias. *Encyclopedia of social science research methods* 2004:1045-46.
45. World Health Organization. Declaration of Alma-Ata: International Conference on Primary Health Care, Alma-Ata, USSR, 6–12 September 1978. *Retrieved February* 1978;14:2006.
46. United Nations. Sustainable Development Goals. [Internet]. Available from: https://www.un.org/sustainabledevelopment/sustainable-development-goals/2018.
47. Ramanayake R. Structured printed referral letter (form letter); saves time and improves communication. *Journal of family medicine and primary care* 2013;2(2):145.
48. Government of India - Press Information Bureau. Health Ministry launches a new state-of-the art Information Platform to monitor public health surveillance 2018 [Available from: http://pib.nic.in/PressReleaseIframePage.aspx?PRID=1553941 accessed 01/01/2019 2019.
49. Doyle M-A, Malcolm JC, Liu D, et al. Using a structured discharge letter template to improve communication during the transition from a specialized outpatient diabetes clinic to a primary care physician. *Canadian journal of diabetes* 2015;39(6):457-66.
50. Lilford RJ, Kelly M, Baines A, et al. Effect of using protocols on medical care: randomised trial of three methods of taking an antenatal history. *Bmj* 1992;305(6863):1181-4.
51. Ibrahim H, Munkhbayar U, Toivgoo A, et al. Can universal patient-held health booklets promote continuity of care and patient-centred care in low-resource countries? The case of Mongolia. *BMJ Qual Saf* 2019:bmjqs-2018-008941.

For peer review only - http://bmjopen.bmj.com/site/about/guidelines.xhtml

S1. Additional information regarding the Indian healthcare system

1. National context: structure of public healthcare system

The basic structure of the public healthcare system in India is as follows:[1]

- National level: Ministry of Health and Family Welfare.
- State level: State Department of Health and Family welfare in each state.
- Regional level: covers 3 - 5 districts. Headed by State Directorate of Health.
- District level: Middle level management organisation serving as a link between the regional and state structures and the peripheral and PHC structures.
- Sub-divisional/Taluk level: Hospitals/hospitals with specialty care (Taluk headquarters hospitals). Healthcare services are rendered via the office of Assistant District Health and Family Welfare Officer.
- Community level: CHCs that cater for 80,000-120,000 population and PHCs that cover approximately 20,000 – 30,000 population (often upgrades of rural dispensaries).

2. Public healthcare across India

The quality of public healthcare across India varies notably between states and between urban and rural areas. Aside from some pockets of excellence in a select few states, the public sector is generally falling short of meeting the basic healthcare needs of the growing population. Some of the main reasons for this include: services being too far away, a lack of trained personnel and supplies, and limited facility

For peer review only

opening times that are often unreliable.[2] With regard to primary health centres, government estimates indicate that 10% are without a doctor, 37% are without a laboratory technician and 25% are without a pharmacist.[3] Issues with public health centres are particularly rife within poor communities, where facilities have been found to be closed more than half the time and lack basic medical supplies. Public facilities are the often the only source of qualified healthcare professionals in rural areas, which is where much of the poor live.[2]

3. National context: private healthcare

Public healthcare in India has lacked funding over a series of decades, resulting from a lack of prioritisation from economic planners. Therefore, the increasing prevalence of chronic, non-communicable diseases alongside unresolved challenges of infectious diseases has placed more strain on public health systems than what can feasibly be managed. The private healthcare sector has subsequently proliferated to meet rising needs, expectations and incomes and surveys indicate that private healthcare providers now dominate service provision. According to reports, public facilities provided just 20% of primary and community-level healthcare services, and 40% of hospital visits in 2004-05 (down from 25% and 60% respectively in 1986-87).[2] At the higher end of the market the private sector has world class facilities that have grown substantially. As a result, hospital care is now an export sector for medical tourism that cares for approximately 200,000 foreign patients per year.[4] However, private healthcare providers are poorly regulated, with uneven quality across facilities. This is resulting in a large number of private facilities delivering services without appropriate equipment or expertise. Additionally, although visiting private providers is preferential for many, it is common for high out-of –pocket costs

For peer review only

to be incurred; more than 40% of all private hospital inpatients have to borrow money or sell assets in order to fund their care.[5] This means that many poorer patients are unable to access healthcare while others fall into poverty as a result of spending.

REFERENCES

1. Care Continuum Alliance, India. India – Health Care System Structure and Organization. 2009. Available from: http://dmaa.pbworks.com/w/page/17960772/India%20-%20Health%20Care%20System%20Structure%20and%20Organization

2. Joumard I, Kumar A. Improving health outcomes and health care in India. Paris: Organisation for Economic Cooperation and Development (OECD) Working Papers, No. 1184, OECD Publishing. 2015. Available from: http://www.oecd-ilibrary.org/economics/improving-health-outcomes-and-health-care-in-india_5js7t9ptcr26-en

3. Ministry of Health and Family Welfare, Government of India. Rural Health Statistics in India. 2012. Available from: https://nrhm.gujarat.gov.in/images/pdf/publications/RHS_2012.pdf

4. Lunt N, Smith R, Exworthy M, Green S, Horsfall D, Mannion R. Medical Tourism: Treatments, Markets and Health System Implications: a Scoping Review. OECD Publishing. 2010. Available from: Paris http://www.oecd.org/els/health-systems/48723982.pdf

5. The World Bank - Health, Nutrition, Population Sector Unit India South Asian Region. Raising the sights: better health systems for India's poor. Available from: https://openknowledge.worldbank.org/handle/10986/14080

For peer review only

S2. S1. Additional information regarding the study settings

2.1.1. Study setting - Himachal Pradesh

Himachal Pradesh is principally a rural state in northern India. It has a population of 6.86 million people and the average literacy rate is 83.3%, which is higher than the national average (74%). However, rates remain notably lower for women compared to men (76.6% vs. 90.8%, respectively).[6] Private HCPs are less prevalent in Himachal Pradesh and public healthcare utilisation remains relatively high.[7] A recent study found that the availability of public health services in the state was deemed adequate as compared to standards of other hill states, but with an unequal distribution of resources across regions.[8]

2.1.2 Study setting - Kerala

Kerala state is in the south-west of India. It has a population of 34.8 million people and a greater than national average urban-based population of 47.7%. It has the highest overall literacy rate in India (93.9%; men 96.1%, women 92.1).[9] There are a relatively large number of government healthcare facilities in Kerala but the healthcare environment is becoming increasingly complex due to a growing presence of private healthcare providers. Despite this, public health facilities in Kerala are generally the first point of care and continue to deliver essential services.[10]

For peer review only

REFERENCES

6. Government of India. Himachal Pradesh Population Census Data 2011 [Internet]. Available from: https://www.census2011.co.in/census/state/himachal+pradesh.html accessed 13/06/18 2017.

7. Government of India. Himachal Pradesh Development Report. New Delhi: Academic Foundation, 2005.

8. Suman.; Bhutani S. Availability of health services in Himachal Pradesh *International Journal of Advanced Research and Development* 2017;2(6):231-38.

9. Government of Kerala. Kerala Population Census Data 2011 [Internet]. Available from: https://www.census2011.co.in/census/state/kerala.html accessed 13/06/18.

10. Government of Kerala. Kerala Development Report. New Delhi: Academic Foundation, 2008.

For peer review only

S5. **Figure 1. Similarities and differences between the content of IP & HCP data with related sub-categories**

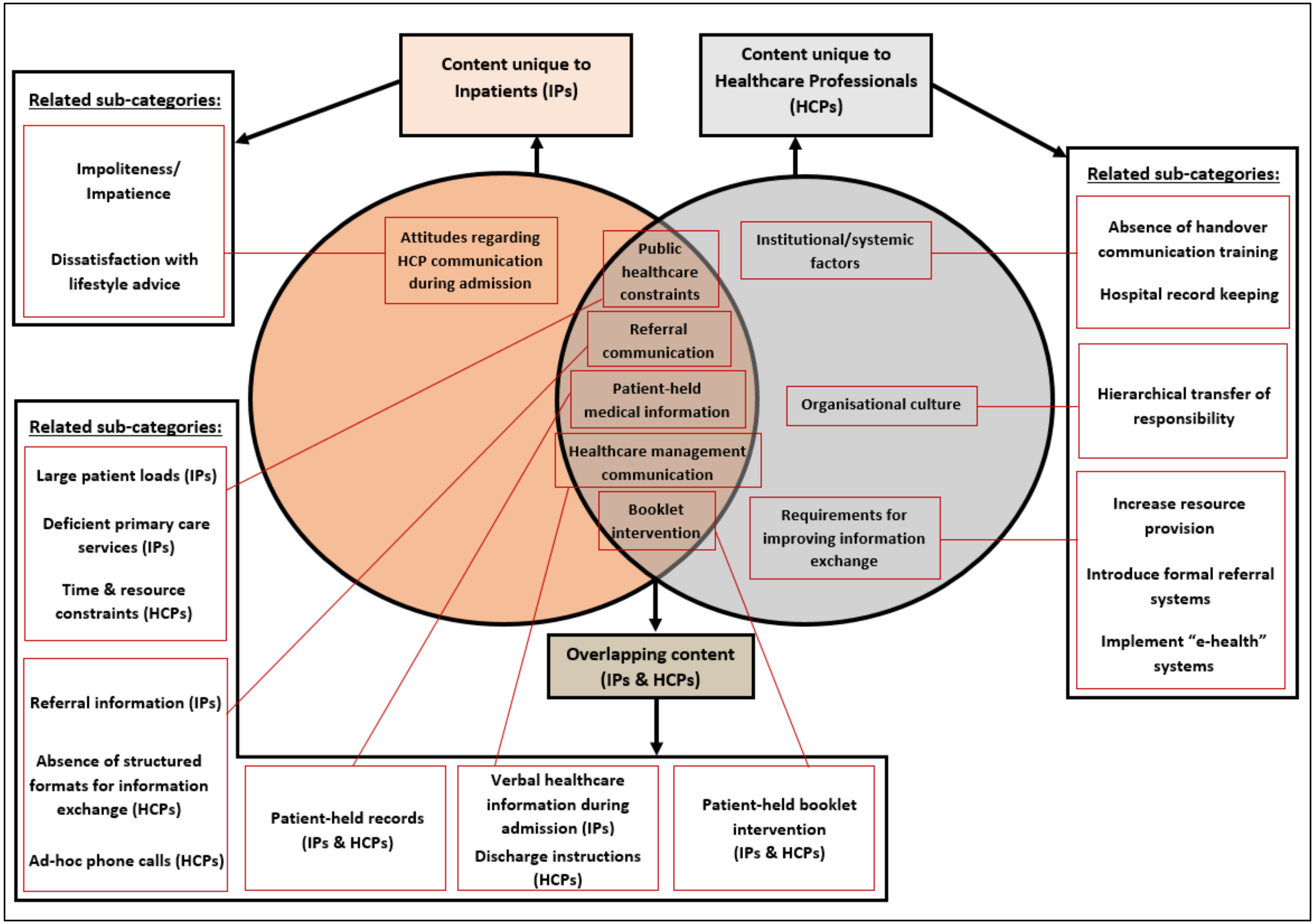

Reporting checklist for qualitative study.

Based on the SRQR guidelines.

Instructions to authors

Complete this checklist by entering the page numbers from your manuscript where readers will find each of the items listed below.

Your article may not currently address all the items on the checklist. Please modify your text to include the missing information. If you are certain that an item does not apply, please write "n/a" and provide a short explanation.

Upload your completed checklist as an extra file when you submit to a journal.

In your methods section, say that you used the SRQR reporting guidelines, and cite them as:

O'Brien BC, Harris IB, Beckman TJ, Reed DA, Cook DA. Standards for reporting qualitative research: a synthesis of recommendations. Acad Med. 2014;89(9):1245-1251.

		Reporting Item	Page Number
	#1	Concise description of the nature and topic of the study identifying the study as qualitative or indicating the approach (e.g. ethnography, grounded theory) or data collection methods (e.g. interview, focus group) is recommended	1
	#2	Summary of the key elements of the study using the abstract format of the intended publication; typically	2-3

For peer review only

		includes background, purpose, methods, results and conclusions	
Problem formulation	#3	Description and signifcance of the problem / phenomenon studied: review of relevant theory and empirical work; problem statement	5-7
Purpose or research question	#4	Purpose of the study and specific objectives or questions	6-7
Qualitative approach and research paradigm	#5	Qualitative approach (e.g. ethnography, grounded theory, case study, phenomenolgy, narrative research) and guiding theory if appropriate; identifying the research paradigm (e.g. postpositivist, constructivist / interpretivist) is also recommended; rationale. The rationale should briefly discuss the justification for choosing that theory, approach, method or technique rather than other options available; the assumptions and limitations implicit in those choices and how those choices influence study conclusions and transferability. As appropriate the rationale for several items might be discussed together.	6-12
Researcher characteristics and reflexivity	#6	Researchers' characteristics that may influence the research, including personal attributes, qualifications / experience, relationship with participants, assumptions and / or presuppositions; potential or actual interaction between researchers' characteristics and the research	6-12

For peer review only

		questions, approach, methods, results and / or transferability	
Context	#7	Setting / site and salient contextual factors; rationale	7
Sampling strategy	#8	How and why research participants, documents, or events were selected; criteria for deciding when no further sampling was necessary (e.g. sampling saturation); rationale	7
Ethical issues pertaining to human subjects	#9	Documentation of approval by an appropriate ethics review board and participant consent, or explanation for lack thereof; other confidentiality and data security issues	37
Data collection methods	#10	Types of data collected; details of data collection procedures including (as appropriate) start and stop dates of data collection and analysis, iterative process, triangulation of sources / methods, and modification of procedures in response to evolving study findings; rationale	6-12
Data collection instruments and technologies	#11	Description of instruments (e.g. interview guides, questionnaires) and devices (e.g. audio recorders) used for data collection; if / how the instruments(s) changed over the course of the study	6-12
Units of study	#12	Number and relevant characteristics of participants, documents, or events included in the study; level of participation (could be reported in results)	14-17

For peer review only

Data processing	#13	Methods for processing data prior to and during analysis, including transcription, data entry, data management and security, verification of data integrity, data coding, and anonymisation / deidentification of excerpts	12-13
Data analysis	#14	Process by which inferences, themes, etc. were identified and developed, including the researchers involved in data analysis; usually references a specific paradigm or approach; rationale	12-13
Techniques to enhance trustworthiness	#15	Techniques to enhance trustworthiness and credibility of data analysis (e.g. member checking, audit trail, triangulation); rationale	12-13
Syntheses and interpretation	#16	Main findings (e.g. interpretations, inferences, and themes); might include development of a theory or model, or integration with prior research or theory	14-28
Links to empirical data	#17	Evidence (e.g. quotes, field notes, text excerpts, photographs) to substantiate analytic findings	21-28
Intergration with prior work, implications, transferability and contribution(s) to the field	#18	Short summary of main findings; explanation of how findings and conclusions connect to, support, elaborate on, or challenge conclusions of earlier scholarship; discussion of scope of application / generalizability; identification of unique contributions(s) to scholarship in a discipline or field	29-32

For peer review only

Limitations	#19	Trustworthiness and limitations of findings	33
Conflicts of interest	#20	Potential sources of influence of perceived influence on study conduct and conclusions; how these were managed	37
Funding	#21	Sources of funding and other support; role of funders in data collection, interpretation and reporting	37

The SRQR checklist is distributed with permission of Wolters Kluwer © 2014 by the Association of American Medical Colleges. This checklist was completed on 26. November 2018 using http://www.goodreports.org/, a tool made by the EQUATOR Network in collaboration with Penelope.ai

For peer review only

BMJ Open

Patient and healthcare provider knowledge, attitudes and barriers to handover and healthcare communication during chronic disease inpatient care in India: A qualitative exploratory study

Journal:	*BMJ Open*
Manuscript ID	bmjopen-2018-028199.R2
Article Type:	Original research
Date Submitted by the Author:	17-Oct-2019
Complete List of Authors:	Humphries, Claire; University of Birmingham, UK, Institute of Applied Health Research<br>Jaganathan, Suganthi ; Centre for Chronic Disease Control<br>Panniyammakal, Jeemon ; Centre for Chronic Disease Control<br>Singh, Sanjeev; Amrita Institute of Medical Sciences and Research Centre; Amrita Institute of Medical Sciences and Research Centre<br>Goenka, Shifalika; Public Health Foundation of India<br>Dorairaj, Prabhakaran ; Centre for Chronic Disease Control<br>Gill, Paramjit ; University of Warwick<br>Greenfield, Sheila; University of Birmingham, Primary Care Clinical Sciences<br>Lilford, Richard; University of Warwick, Division of Health and Population Sciences<br>Manaseki-Holland, Semira; University of Birmingham,
**Primary Subject Heading**:	Public health
Secondary Subject Heading:	Health services research, Qualitative research
Keywords:	International health services < HEALTH SERVICES ADMINISTRATION & MANAGEMENT, Quality in health care < HEALTH SERVICES ADMINISTRATION & MANAGEMENT, PUBLIC HEALTH, QUALITATIVE RESEARCH

SCHOLARONE™
Manuscripts

Patient and healthcare provider knowledge, attitudes and barriers to handover and healthcare communication during chronic disease inpatient care in India: A qualitative exploratory study

Claire Humphries[1¶], Suganthi Jaganathan[2,3¶], Jeemon Panniyammakal[2,3,4], Sanjeev Singh[5], Shifalika Goenka[2,3], Prabhakaran Dorairaj[2,3], Paramjit Gill[6], Sheila Greenfield[1], Richard Lilford[7], Semira Manaseki-Holland[1*]

[1] Institute of Applied Health Research, University of Birmingham, Birmingham, United Kingdom

[2] Public Health Foundation of India, Delhi, India

[3] Centre for Chronic Disease Control, Delhi, India

[4] Sree Chitra Tirunal Institute of Medical Sciences and Technology, Kerala, India

[5] Amrita Institute of Medical Sciences, Kerala, India

[6] Academic Unit of Primary Care, University of Warwick, Coventry, United Kingdom

[7] Centre for Applied Health Research and Delivery, University of Warwick, Coventry, United Kingdom

*Corresponding author

Email: S.ManasekiHolland@bham.ac.uk

¶ These authors contributed equally to this work

For peer review only - http://bmjopen.bmj.com/site/about/guidelines.xhtml

For peer review only

ABSTRACT

Objectives To investigate patient and healthcare provider (HCP) knowledge, attitudes and barriers to handover and healthcare communication during inpatient care and explore interventions for improving the storage and transfer of healthcare information.

Methods *Design:* Qualitative study comprising 41 semi-structured, individual interviews. Thematic analysis using the Framework Method with analyst triangulation. *Setting*: Three public hospitals in Himachal Pradesh and Kerala, India. *Participants:* 20 male (n=10) and female (n=10) chronic non-communicable disease (NCD) patients and 21 male (n=15) and female (n=6) HCPs. Purposive sampling was used to identify patients with chronic NCDs (Cardiovascular Disease, Chronic Respiratory Disease, Diabetes or Hypertension) and HCPs.

Results Patient themes: (1) *Public healthcare service characteristics;* (2) *HCP-patient communication;* (3) *Attitudes regarding medical information.* HCP themes: (1) *System factors;* (2) *Information exchange practices;* (3) *Quality improvement strategies.* Both patients and HCPs recognised public healthcare constraints that increased pressure on hospitals and subsequently limited consultation times. Systemic issues reported by HCPs were a lack of formal handover systems, training and accessible hospital-based records. Healthcare management communication during admission was inconsistent and lacked patient-centeredness, evidenced by varying patient of information received and some dissatisfaction with lifestyle advice. Senior doctors reported passing the duty of writing discharge notes to juniors when busy with high patient loads. Nurses reported predominantly providing verbal discharge instructions to patients. Patient-held documents facilitated information

For peer review only - http://bmjopen.bmj.com/site/about/guidelines.xhtml

exchange between HCPs but were not always transported. HCPs and patients expressed positive views towards the idea of introducing patient-held booklets to improve the organisation and transfer of medical documents.

Conclusions Handover and healthcare communication during chronic NCD inpatient care are currently suboptimal. Structured information exchange systems and HCP training are required to improve continuity and safety of care during critical transitions such as referral and discharge. Our findings suggest that patient-held booklets may also assist in enhancing handover and patient-centred practices.

For peer review only

STRENGTHS AND LIMITATIONS OF THIS STUDY

- This is the first qualitative study, as far as the authors are aware, to explore both patient and healthcare provider knowledge, attitudes and barriers to multiple areas of handover and healthcare communication for chronic disease inpatients in India.
- The number of interviews from both patients and healthcare providers facilitated data saturation and provided a range of significant perspectives.
- Analyst triangulation corroborated data analysis and strengthened the credibility of the study.
- The accuracy of recall of patients interviewed at home (i.e. following hospital discharge) may have been limited by the delay between study recruitment and subsequent data collection.
- Awareness of the interviewer's context as a public health researcher may have resulted in participants distorting their responses to minimise critical judgement.

For peer review only

1. INTRODUCTION

The increasing burden of chronic, non-communicable diseases (NCDs) such as cardiovascular disease, diabetes and chronic respiratory disease has become a global pandemic that is disproportionately affecting low and middle-income countries (LMICs).[1] This is placing great demand on under-resourced health systems that can only be relieved by employing efficient and integrated approaches to healthcare management. Central to efficiency and integration in healthcare is effective handover communication, which involves the exchange of patient-specific information between healthcare providers (HCPs) and between HCPs and patients/carers.[2]

Between HCPs, information exchange is critical during clinical handovers, which are the points in care where information, responsibility and accountability for patient care are transferred from one HCP to another.[3] Such exchange is vital because safe and effective treatment can only be maintained if all relevant information has been shared and understood.[4] A wealth of research from high-income countries (HICs) has evidenced the association between communicative breakdowns during care transitions and risks to patient safety. These risks are pervasive throughout inpatient care and include delays in diagnosis, medication errors and life-threatening adverse events.[5 6] In addition, one-in-five patients experience adverse events following hospital discharge and research has established a link between such events and deficient handover communication.[7-10] Between HCPs and patients, effective information exchange is also vital as patients can provide valuable information to those involved at various stages of their care pathway.[4] Excellent HCP-patient healthcare communication further empowers patients to become active participants in their healthcare management; this is a key

For peer review only - http://bmjopen.bmj.com/site/about/guidelines.xhtml

aspect of patient-centred care, which has been linked to improved patient satisfaction and outcomes.[11]

Despite the established importance of handover communication for health systems functioning and patient safety in HICs, there has been a relative dearth of LMIC-based research focussing on this topic.[12] A recent (2019) study from South Africa has found inadequate discharge planning to be a significant contributor to avoidable causes of hospital readmission.[13] Across India, a handful of predominantly single-site studies have evaluated and described deficiencies in information exchange during referrals, hospital shift-change and discharge.[14-19] The current study forms part of a series completed for a project investigating handover and continuity of care for chronic NCD patients in Kerala and Himachal Pradesh states, India. The first study to have been disseminated focussed on outpatient care, which found issues such as suboptimal recording of information within patient-held medical documents and an absence of formal systems for exchanging information between levels of care.[20]

Given these emerging challenges and the established link between deficient handover communication during inpatient care and risks to patient safety, the current study was conducted to gain novel insight into the transfer of healthcare information during chronic NCD inpatient care across the same study areas of India. The primary objective was to explore knowledge, attitudes and barriers to handover and healthcare communication during the following points of inpatient care: 1) Referral/transfer (i.e. communication between HCPs and between HCPs and patients when referring and/or transferring patients) and 2) Hospital admission and discharge (i.e. communication between HCPs and patients regarding condition, treatment and/or management during hospital admission and discharge). A

For peer review only - http://bmjopen.bmj.com/site/about/guidelines.xhtml

For peer review only

secondary objective was to explore possible interventions to improve the storage and transfer of key healthcare information.

2. METHODS

2.1 Overview

We report findings from a qualitative study of handover communication for chronic NCD inpatients in two Indian states. This study was conducted from December 2014 to November 2015 across three public hospitals: one rural secondary-care hospital in Himachal Pradesh state, and one peri-urban secondary-care and one urban tertiary-care hospital in Kerala state. These settings were selected to capture a range of hospital types within different geographical settings. We selected public rather than private facilities as this is where a large proportion of socioeconomically vulnerable patients access healthcare. See supplementary files "S1" and "S2" for further information regarding the Indian healthcare system and study settings.

2.2 Participant recruitment

2.2.1 Patients

Patients were recruited opportunistically from hospitals by trained research assistants (n=6).[21] Purposive sampling was used to identify individuals who met the following inclusion criteria:[22] adults (18 years+), admitted to hospital within 24 hours

For peer review only - http://bmjopen.bmj.com/site/about/guidelines.xhtml

of a researcher first meeting them, due to complications from one of the following chronic NCDs: cardiovascular disease, chronic respiratory disease, diabetes mellitus, or hypertension. The identification process took place via researchers approaching ward nurses and asking them about patient demographics and admission details; patients were excluded if judged too unwell to participate by ward nurses. Patients who met the inclusion criteria were provided with verbal and documented study information. Written consent was obtained from literate patients. For illiterate patients, oral consent was obtained along with a thumbprint and signature from a literate witness (i.e. family member/carer) in line with World Health Organisation guidelines.[23] Patients were recruited until theoretical saturation was achieved;[24] A total of 20 patients participated.

2.2.2 Healthcare Professionals

Healthcare professionals (HCPs) were recruited from study hospitals by trained research assistants (n=6). Due to the busy nature of the study settings, opportunistic sampling was used to recruit as many HCPs as possible with a range of roles and experience.[21] During recruitment, if HCPs stated they were too busy to answer questions they were marked as “unavailable” and not approached again that day; this did not exclude them from participating at another time. HCPs were also recruited until theoretical saturation was achieved;[24] A total of 21 HCPs participated.

2.3 Sample Size

For peer review only - http://bmjopen.bmj.com/site/about/guidelines.xhtml

As well as saturation being reached for both participant groups independently, the resulting sample size of 41 participants for this study was in accordance with Baker and Edwards' review of sample sizes utilised in qualitative literature, indicating it was sufficient for achieving overall data saturation.[25]

2.4 Data collection

The inpatient data analysed for this study is independent from the outpatient study and was collected from different patients using separate topic guides. Regarding HCP data, this study involves secondary analysis of HCP interviews (n=17) included in the outpatient study from participants who were also involved in inpatient care. A small number of additional interviews with HCPs solely involved in inpatient care (n=4) have also been analysed in this study. All HCP interviews in the India handover project were conducted within the same study period and used the same topic guide (as most HCPs in the study areas worked with both outpatients and inpatients on a daily basis).

All interview data was collected entirely by the lead Indian researcher (SJ – an experienced public health researcher) who was familiar with, but not local to, all study areas and fluent in all local Indian dialects and English. Full consideration was given prior to and throughout data collection to ensure that SJ was aware of the potential limitations of working with participants from culturally and linguistically diverse backgrounds. SJ was not involved in the treatment of patients or previously known to HCPs.

The majority (n=16) of patient interviews took place in study hospitals. Due to a lack of private spaces, they were conducted on inpatient wards in as quiet and

For peer review only - http://bmjopen.bmj.com/site/about/guidelines.xhtml

private a manner as possible. All participants consented to this and it was ensured that HCPs were not present during patient interviews. In addition, a small number of patient interviews (n=4) took place in patients' homes either five weeks (n=2) or four months (n=2) following hospital discharge, as this was more convenient for them (i.e. during recruitment they were in the process of being discharged and leaving hospital). The specific follow-up times coincided with community visits being completed for another quantitative study within the India handover project, which the four patients were also participating in. All HCP interviews took place in hospital offices. Interviews with patients and HCPs were conducted in either Hindi, English, Malayalam or a mixture depending on interviewee preference and audio recorded using a digital Dictaphone.

Data collection took place in two stages. In the first stage (December 2014–October 2015), pre-prepared topic guides were used to guide interviews. These were developed using relevant handover literature and local knowledge of health systems functioning within the study areas. They were also piloted over three rounds prior to commencement of data collection to ensure they were clear as well as culturally and contextually appropriate. Patient topic guides included open-ended questions focussing on healthcare utilisation and experiences and attitudes of healthcare visits and information exchange. The HCP topic guides differed slightly to capture information on health systems policies and/or practices; it also included questions regarding handover training and potential interventions for improving practices.

Following the first stage of data collection, on the 11th of October 2015, a handover expert meeting took place in Delhi, India to present preliminary findings and discuss possible interventions. Researchers from the University of Birmingham and the University of Warwick (UK) facilitated the presentation of results and group

For peer review only - http://bmjopen.bmj.com/site/about/guidelines.xhtml

discussions at the meeting. Representatives (n=27) from the following international, Indian national and state-level organisations participated: The World Health Organisation; The World Bank; ACCESS Health International; The Ministry of Health and Family Welfare; The Public Health Foundation of India; The National Centre for Disease Control; The Centre for Chronic Disease Control; The National Health System Resource Centre; The All India Institute of Medical Sciences; Aga Khan Health Services; AMRITA Institute of Medical Sciences and Fortis Hospitals. During discussions, a consensus was reached that patient-held booklets were likely to be an acceptable and sustainable intervention to improve information exchange. This was based on the international success of similar patient-held records used in maternal healthcare around the world.[26-30] It also took into account the delays in developing universal electronic information systems and the fact that such systems will not necessarily address the quality of communication between HCPs and patients. Overall, it was opted as the most pragmatic, cost-effective intervention and multiple experts felt that booklets could also improve patient self-management if they contained disease-specific advice.

Therefore, following the meeting the second stage of qualitative data collection (October–November 2015) commenced. Topic guides were updated to include questions regarding the utility of patient-held booklets. In addition, if participants stated they had limited time then researchers interviewed them using a shortened topic guide containing targeted questions on patient-held booklets.

2.5 Data Analysis

For peer review only - http://bmjopen.bmj.com/site/about/guidelines.xhtml

All audio recordings of interviews were transcribed verbatim and, if necessary, translated into English by SJ. All translations were crosschecked for accuracy by a qualitative expert in India (SG – professor of bioethics and social and behavioural sciences with expertise in NCDs), who was also familiar with the local context and fluent in all languages used during interviews. Following this, all transcripts were sent to the lead UK researcher (CH – public health PhD student) for analysis. CH became familiar with all study sites prior to analysis during multiple research-related visits that were facilitated by the Public Health Foundation of India and the Ministry of Health and Family Welfare in Kerala.

Data was analysed using the Framework Method,[31] as this is the method most commonly used for semi-structured interview transcripts. An inductive thematic approach to analysis utilised in Grounded Theory was employed,[32 33] which focused on analysing interviews in their entirety and identifying concepts relevant to handover and healthcare communication during inpatient care that emerged from interviews. Analysis occurred through the following stages central to the Framework Method: transcription, familiarisation, coding, charting, and interpretation. Over a one-month period, familiarisation with the data took place via the slow reading of transcripts and CH consulted with SJ to gain a clear understanding of interview contexts. Once this was complete, coding began and two transcripts were chosen at random from each batch of interviews (i.e. 2 patient and 2 HCP transcripts) for independent coding by an additional UK analyst (SG – professor of medical sociology with expertise in cross-cultural research) for analyst triangulation.[34] Patient and HCP transcripts were coded separately in order to be able to assess similarities and differences between participant groups - patient transcripts were coded first. The coding process involved further familiarisation with the data, followed by open coding where certain transcript

For peer review only - http://bmjopen.bmj.com/site/about/guidelines.xhtml

content was highlighted and allocated descriptive labels (codes) to interpret the phenomena identified in the text. The development of codes and themes was entirely data-led and analysed manually.[35]

Microsoft Excel was used to organise participant codes. CH created initial categories by clustering similar codes developed from the two randomly selected patient and HCP transcripts. CH and the additional UK analyst (SG) then met to discuss their analyses. As both had produced similar codes and concepts, the categories that were created were mutually agreed upon. CH then continued with category development until all transcripts had been coded and inserted into the spreadsheet. Following analysis of 20 patient and 21 HCP transcripts, no new categories had been produced. This served as confirmation that data saturation had been met.[24]

Following coding, categories were grouped into subcategories and linked to produce themes. Then, via the process of charting,[31 35] themes for each participant group were used to create a framework matrix into which participants' quotes were inserted, corresponding to their representative subcategory. This provided a visual representation of themes, which facilitated the mapping and interpretation of the data. After completing separate analysis of patient and HCP data, the results of both participant groups were compared to assess similarities and differences between their reports of knowledge, attitudes and barriers to handover and healthcare communication. A Venn diagram was used to summarise the separate and overlapping content, which was linked to subcategories from original themes.

2.7 Patient and public involvement

For peer review only - http://bmjopen.bmj.com/site/about/guidelines.xhtml

Patients and the public were not involved in the initial design of this study. Patients and carers were first involved during the pilot phase prior to formal data collection, where the topic guides, consent and information sheets were piloted over three rounds. During this time, they were consulted and given the opportunity to provide feedback to ensure the study materials were comprehensible and culturally and contextually appropriate. Patients and the public were not involved in any other aspect of the study recruitment or conduct, but findings have been disseminated publicly via an expert meeting (including professionals working with patient groups) and open access web pages.

3. RESULTS

3.1 Patient characteristics

20 male (n=10) and female (n=10) patients aged between 25 and 72 years old were interviewed. Participants' background characteristics were varied (Table 1). Patients completed interviews in English (n=11), Hindi (n=4), Malayalam (n=4) and a mixture of Hindi and English (n=1).

For peer review only - http://bmjopen.bmj.com/site/about/guidelines.xhtml

Table 1. Patient characteristics

Characteristics	1	2	3	4	5	6	7	8	9	10	11	12	13	14	15	16	17	18	19	20	N (%)
**Age**	65	45	70	58	71	56	57	70	55	25	72	50	55	69	70	50	70	70	70	70	25-72
**Sex**																					
Male	√	√	√	√	√				√	√	√	√				√					10 (50.0)
Female						√	√	√					√	√	√		√	√	√	√	10 (50.0)
**Literacy**																					
Illiterate								√					√		√	√	√	√	√	√	8 (40.0)
Literate	√	√	√	√	√	√	√		√	√	√	√		√							12 (60.0)
**Education Level**																					
None/minimal primary school-level								√				√	√		√	√	√	√	√	√	9 (45.0)
Completed lower primary school					√		√														2 (10.0)
Completed upper primary school						√															1 (5.0)
Completed secondary school									√												1 (5.0)
University graduate (or above)	√			√						√	√										4 (20.0)
No data		√	√											√							3 (15.0)
**Employment Status**																					
Employed		√		√	√				√			√	√	√		√					8 (40.0)
Unemployed						√	√	√							√		√	√	√	√	8 (40.0)
Student										√											1 (5.0)
Retired	√		√								√										3 (15.0)
**Chronic NCD(s) (related to admission)***																					
Chronic Respiratory Disease			√	√		√		√					√			√					6 (30.0)
Diabetes	√													√	√	√	√	√	√	√	8 (40.0)
Hypertension									√		√	√				√	√	√			6 (30.0)
Cardiovascular Disease (other than Hypertension alone)	√	√			√		√	√	√	√	√	√									9 (45.0)
**Language(s) used during Interview**																					
English (only)	√	√	√	√										√	√	√	√	√	√	√	11 (55.0)
Hindi (only)						√	√	√	√												4 (20.0)
Malayalam (only)										√	√	√	√								4 (20.0)
English & Hindi (mixture)					√																1 (5.0)

* Patients could select more than one answer for this question

For peer review only

3.2 Healthcare Professional characteristics

21 male (n=15) and female (n=6) HCPs aged between 22 and 55 years old were interviewed. HCP roles included doctors (n=17), nurses (n=2), pharmacists (n=1) and a medical records officer (n=1). HCP qualifications and experience were varied (Table 2). HCPs completed interviews in English (n=15), Hindi (n=2), Malayalam (n=2) and a mixture of Hindi and English (n=2).

For peer review only

Table 2. Healthcare professional characteristics

Characteristics	1	2	3	4	5	6	7	8	9	10	11	12	13	14	15	16	17	18	19	20	21	N (%)
**Age**	44	24	33	25	23	39	44	35	52	50	50	43	50	40	46	55	22	35	35	45	35	22-55
**Sex**																						
Male	✓		✓	✓	✓		✓		✓	✓	✓	✓	✓	✓	✓	✓	✓			✓		15 (71.4)
Female		✓				✓		✓										✓	✓		✓	6 (28.6)
**Qualification/s***																						
Doctor of Medicine (MD)	✓		✓				✓	✓	✓	✓	✓	✓	✓	✓	✓	✓	✓					13 (61.9)
Master of Public Health (MPH)						✓																1 (4.8)
Bachelor of Medicine (MBBS)	✓	✓	✓	✓	✓	✓	✓	✓	✓	✓	✓	✓	✓	✓	✓	✓	✓					17 (81.0)
BSc Nursing																		✓			✓	2 (9.5)
BSc Pharmacy																			✓			1 (4.8)
Graduate (i.e. non-medical degree)																				✓		1 (4.8)
**Official position**																						
Medical Superintendent						✓																1 (4.8)
Chief Medical Officer									✓													1 (4.8)
Medical Officer												✓										1 (4.8)
Consultant	✓		✓				✓	✓					✓	✓	✓	✓	✓					9 (42.9)
Surgeon		✓								✓												2 (9.5)
General Medicine											✓											1 (4.8)
Intern Doctor				✓	✓																	2 (9.5)
Ward Nurse																		✓			✓	2 (9.5)
Pharmacist																			✓			1 (4.8)
Medical Records Officer																				✓		1 (4.8)
**Years of experience in position**																						
<1		✓			✓																	2 (9.5)
1 – 3				✓																		1 (4.8)
4 – 6						✓		✓										✓		✓	✓	5 (23.8)
7 – 10			✓				✓												✓			3 (14.3)
>10	✓								✓	✓	✓	✓	✓	✓	✓	✓	✓					10 (47.6)
**Place of work**																						
General Hospital		✓	✓	✓	✓									✓		✓	✓	✓	✓			9 (42.9)
Regional Hospital	✓								✓	✓	✓		✓							✓	✓	7 (33.3)
Taluk Hospital						✓	✓	✓				✓			✓							5 (23.8)
**Language(s) used during interview**																						
English (only)		✓	✓	✓	✓	✓	✓	✓	✓	✓	✓	✓		✓	✓	✓	✓					15 (71.4)
Hindi (only)																				✓	✓	2 (9.5)
Malayalam (only)																		✓	✓			2 (9.5)
English & Hindi (mixture)	✓												✓									2 (9.5)

* HCPs could select more than one answer for this question

For peer review only - http://bmjopen.bmj.com/site/about/guidelines.xhtml

3.3 Charted Data

During analysis of patient and HCP data, three themes (with subcategories) emerged for each participant group. Patient themes were: 1) Public healthcare service characteristics, 2) HCP to patient communication and 3) Attitudes regarding medical information (Table 3). HCP themes were: 1) System factors, 2) Information exchange practices and 3) Quality improvement strategies (Table 4).

Following separate analysis of patient and HCP data, the results of both participant groups were compared to assess similarities and differences between their reports of knowledge, attitudes and barriers to handover and healthcare communication; the results of this comparison are displayed in Figure 1. The similarities will be described first, followed by the differences. To ensure confidentiality, numerical pseudonyms have been used when presenting quotes.

For peer review only

Table 3. Summary of charted data for inpatients (IPs)

	Public healthcare service characteristics		HCP to patient communication			Attitudes regarding medical information		
IP	Large patient loads	Deficient primary care services	Verbal healthcare information during admission	Referral information	Impoliteness/ impatience	Transportation of medical documents	Patient-held booklet intervention	Dissatisfaction with lifestyle advice
1						✓		
2			✓					
3	✓		✓		✓	✓		
4			✓			✓		
5		✓	✓	✓	✓			
6				✓		✓		
7	✓	✓		✓		✓		
8				✓				
9			✓					
10			✓					
11	✓		✓			✓		
12			✓	✓		✓		
13			✓			✓		
14		✓	✓				✓	✓
15		✓	✓		✓		✓	✓
16		✓	✓			✓	✓	
17			✓			✓	✓	✓
18		✓	✓			✓	✓	
19			✓			✓	✓	
20			✓				✓	✓

For peer review only

Table 4. Summary of charted data for healthcare professionals (HCPs)

	System factors				Information exchange practices				Quality improvement strategies			
**HCP**	**Time & resource constraints**	**Absence of handover communication training**	**Absence of structured formats for information exchange between HCPs**	**Hospital record keeping**	**Ad-hoc phone calls**	**Patient-held medical documents**	**Discharge instructions**	**Hierarchical transfer of responsibility**	**Increase resource provision**	**Introduce formal referral systems**	**Implement "e-health" systems**	**Patient-held booklet intervention**
**1**			✓			✓	✓		✓			
**2**			✓			✓	✓					
**3**						✓			✓	✓	✓	
**4**	✓		✓			✓	✓					
**5**			✓	✓		✓						
**6**	✓		✓						✓		✓	
**7**	✓		✓		✓							
**8**	✓				✓							
**9**									✓			
**10**	✓	✓	✓		✓		✓					
**11**	✓	✓	✓						✓	✓		
**12**				✓		✓			✓		✓	
**13**	✓										✓	✓
**14**	✓											✓
**15**	✓					✓						✓
**16**												✓
**17**	✓							✓				✓
**18**				✓		✓	✓	✓				
**19**	✓			✓								
**20**				✓								
**21**	✓							✓				

For peer review only - http://bmjopen.bmj.com/site/about/guidelines.xhtml

3.4 Overlapping content

3.4.1 Public healthcare constraints

During interviews, a number of patients reported that they chose to visit public hospitals because of the better availability of healthcare staff compared to local healthcare facilities, such as smaller hospitals and primary/community health centres:

"*We have very limited time, we did go to local hospital but doctors are not there. So if we get time we will come here rather than going to a hospital where there are no doctors. (IP 15)"*

However, multiple patients also reported that public hospitals were often crowded with high daily patient loads:

"There is so much crowd there you can't ask or hear anything there… so many people are there now, you cannot do anything. (IP 11)"

The human resource issues at public primary and community healthcare facilities were also mentioned by HCPs:

"It will be useful if availability of doctors is ensured at the peripheral institutions around the clock. At times it is not there. (DOC 1)"

Additionally, in our study settings most hospital doctors worked in both outpatient clinics and inpatient wards on a daily basis. Many doctors expressed concerns of time pressures due to the large patient volumes seen at hospital outpatient clinics and the subsequent lack of time they had to attend to all patients:

"We can hardly spend five minutes with each patient, seeing the crowd you will just want to finish everyone soon. (DOC 7)"

Some doctors also reported that human and medical resource constraints across public healthcare facilities were hindering the quality of care that could be provided:

"[It's] not [about] motivation, [it's about] resource limitation. It's not humanly possible to see people every day for seven days. Quality definitely gets compromised. (DOC 3)"

3.4.2 Referral communication

A number of patients who recalled being referred from a previous healthcare facility to the hospital reported that they were not provided with any referral information:

"No, they didn't give any parchi [papers]. We were getting medicines right only that is with us. (IP 8)"

HCPs also discussed referral communication. Doctors explained that there were no structured processes to follow for information exchange during referrals:

"Yeah there is no proper way of doing it… inpatients sometimes we have to [refer] but as I told you we never had a structured format. (DOC 14)"

Despite the lack of structured systems, some doctors explained that they would make ad-hoc calls to ensure that some information was transferred when referring a patient. However, this appeared to depend on how well they knew the patient or doctor:

For peer review only

"Sometimes I call the doctor to tell them that so and so is coming. Please do the needful. If I know the patient or doctor. (DOC 11)"

3.4.3 Patient-held medical information

At the point of hospital admission, patient-held notes and/or medical records can facilitate optimal care by providing HCPs with key patient-specific information. When asked about whether they brought medical papers to the hospital, most patients reported that they regularly stored and transported papers to HCP visits; these included referral notes, prescription cards, test results and other records from inpatient/outpatient/primary care:

""Yeah we have always kept everything safely. [Shows researcher a bag with all sort of papers like reports, lab tests, etc.] (IP 3)"

Doctors also talked about patient-held medical information during interviews. For example, some doctors reported that patients regularly kept and transported their medical records:

"Almost everyone comes with medical reports. (DOC 11)"

However, other doctors described that, in their experience, the availability of patient-held records was less consistent and that this could have a negative impact on the continuity of care provided:

"Some of them do bring investigations and all others don't bring much and we have to work out what happened from the start. (DOC 3)"

For peer review only - http://bmjopen.bmj.com/site/about/guidelines.xhtml

3.4.4 Healthcare management communication

When asked about verbal HCP communication, many patients reported that during admission and/or discharge a HCP had provided them with some basic verbal healthcare management information (i.e. medication, treatment, lifestyle and/or follow-up requirements). However, the quantity of information received appeared to vary notably between patients. For example, some recalled being given detailed instructions:

“Doctor says everything. I was given medicines and now they asked me to take injections also. Doctor is saying I am not controlling my sugar. The nurse taught me how to take injection. (IP 19)”

Conversely, others appeared to receive relatively limited information and one carer reported having to seek healthcare advice from alternative sources:

“Doctors don’t explain everything. We speak to our friends and get details from them. (Carer - IP 16)”

HCPs also discussed their healthcare communication practices with patients. Whilst talking about discharge, a nurse described the usual amount of time taken to explain information to each patient:

“Usually we take 20 to 25 minutes to instruct the patients. If the patients understand then it can be even faster. (NUR 1)”

Doctors reported that they provided patients with documented information on discharge cards and verbally advised patients to return to their local HCP/healthcare institution during the discharge consultation:

For peer review only - http://bmjopen.bmj.com/site/about/guidelines.xhtml

"We give them a discharge card. Discharge card is there we have written and then we refer them to the local hospital or where they come from. (DOC 15)"

3.4.5 Booklet intervention

During interviews that took place after the handover expert meeting, patients were asked for their opinion regarding the utility of a patient-held booklet where records could be stored, organised and transported to HCP visits. Most appeared to think that it could be effective and help with self-management, including those who were illiterate:

"Yeah, sometimes we don't know what to do so it would be good if some paper is there to help us. We can't read it ourselves but our son or daughter-in-law can help us. (IP 17)"

HCPs were also asked for their opinions regarding the booklet intervention. Many generally felt it could be useful, but various conditions and/or reservations were also expressed. For example, doctors felt that the success of the booklet would rely on patient attitudes:

"That will depend on the patients, if they maintain that and bring it every time. For us there is no change, we write our observations in paper or notebook, doesn't matter… Might be helpful. (DOC 22)"

Related to this, one doctor felt that in order to see the most benefit, patients needed to be regularly instructed to keep and transport their medical documents:

"We write the communication but the patients don't keep them proper. I think we have to tell the patients to keep the letters and papers. (DOC 4)"

For peer review only - http://bmjopen.bmj.com/site/about/guidelines.xhtml

3.5 Content unique to patients

3.5.1 Attitudes regarding HCP communication during admission

A few patients recalled receiving some impolite/impatient treatment from healthcare staff during their hospital admission:

"The doctors don't speak much. They explain but get angry if you don't understand them. (IP 3)"

In addition, some patients expressed dissatisfaction with the lifestyle advice provided. In particular, patients of lower socioeconomic status felt that nutritional instructions were not suitable for them due to their time and financial constraints:

"We are daily labourers we can't follow all the instructions… We can't follow that, we are poor we do hard work and we just can't concentrate on eating. Whatever is there we just eat. (IP 15)"

3.6 Content unique to HCPs

3.6.1 Institutional/systemic factors

Some doctors displayed good knowledge of the key information that should be transferred during patient referrals/transfers and/or hospital discharge.

For peer review only - http://bmjopen.bmj.com/site/about/guidelines.xhtml

"To another hospital, yeah first we have to write what are the main complaints of patients presenting illness and write about the past history, then we will write about what all investigations we have done here 'til the day of transfer, then what is the condition of the patient we are discharging, why we are discharging (and) any investigations, major investigations, to be done. (DOC 2)"

However, when asked about training opportunities, numerous doctors mentioned that they had not received any formal handover training. Some recalled that this type of training was not provided at medical school:

"I think it was not there in medical curriculum. (DOC 1)"

Others reported that training was not provided in their workplace/s and instead they learned on the job:

"We are sent to the wards, we see what our seniors do and we do that's all. We have to develop our communication skills ourselves no formal training is there. (DOC 14)"

When asked about hospital record keeping, a medical records officer stated that inpatient records are stored in hospitals following patient discharge for up to ten years. However, the same officer also indicated that these paper-based records are not easily accessible:

"Definitely I can locate any record but it might take some time to locate them. (MRO 1)"

3.6.2 Organisational culture

For peer review only

Based on reports from both doctors and nurses, it appeared as though some hierarchical transfer of responsibility for documented handover communication took place in hospitals. For example, a senior doctor mentioned that they instructed medical interns to write notes for them when their patient load was high:

"We do write in the papers, whether it's discharge card or outpatient sheets. When patient load is high, then we tell our interns to do it for us, we check that and then sign. (DOC 22)"

3.6.3 Requirements for improving information exchange

During interviews, HCPs were asked for their thoughts on requirements to improve information exchange between HCPs and between HCPs and patients. Numerous doctors felt that there needed to be a notable increase in public healthcare resource provision:

"Infrastructure is very small but the outpatient department is ten times more than it can manage, so more posts should be created… We have to increase the manpower and also our materials. (DOC 15)"

Some doctors also discussed the idea of introducing structured referral documents and systems to improve referral communication:

"You can supply people with [referral] forms and make it mandatory that residents have to maintain a register. In that case they will maintain the register. (DOC 3)"

In addition, whilst discussing current information systems, one doctor in Kerala reported that an application had been made for a near-future transition to

For peer review only - http://bmjopen.bmj.com/site/about/guidelines.xhtml

computerised healthcare information systems. This appeared to be a state-wide plan for public healthcare facilities:

"We have submitted a proposal for paperless computerisation system for doctors, so I think state-wide they are planning to do that. (DOC 6)"

4. DISCUSSION

4.1 Main findings

This study presents qualitative data on patient and HCP knowledge, attitudes and barriers to handover and healthcare communication during public hospital inpatient care in Kerala and Himachal Pradesh states, India. The main findings are that verbal and documented information exchange between HCPs and between HCPs and patients is often suboptimal during referrals/transfers, hospital admission and discharge, with a lack of structured systems and HCP education in place to ensure sufficient continuity of care. Whilst unique themes emerged for both patients and HCPs, a comparison of the results from each participant group showed that there was also a notable amount of overlapping content. The results have highlighted the challenging and multifaceted nature of handover and healthcare communication during inpatient care in India. With regard to public health, the findings have also elucidated a number of key areas to address to improve the continuity and safety of chronic NCD patient care.

For peer review only

Some of the results from the current study reflect and reinforce findings from previous research focussing on outpatient care in the same study areas of India.[20] In particular, during interviews in both studies, patients and HCPs recognised the resource constraints affecting public healthcare. The main issue reported was deficient primary healthcare services, which is in line with well-established findings of limited primary care infrastructure across India and numerous LMICs.[36] In our study settings, under-resourced primary care resulted in many patients preferring to visit hospitals as the first point of care. Subsequently, large patient loads were seen in both outpatient and inpatient departments, which limited HCP consultation times. Other key areas of discussion in the current study reflected in the outpatient findings were inconsistent transportation of patient-held medical documents and views regarding the utility of patient-held booklets. Whilst more inpatients than outpatients reported that they regularly transported records to HCP visits, doctors recalled seeing many patients who did not bring information to the hospital. This was problematic as if patients did not bring their records, then doctors had to gather details from scratch, potentially compromising their continuity of care. When asked about the possible utility of introducing patient-held booklets to store and transport medical documents, inpatients had similar views to outpatients which were generally positive but also felt that the inclusion of self-management information would be beneficial. Doctors in the current study expressed a wider variety of views regarding booklets but broadly thought that they could be useful if patients had positive attitudes towards their maintenance and use.

Regarding referral communication, the current study also highlighted similar issues of deficient information exchange observed in the previous outpatient study.[20] For example, reports from both HCPs and patients revealed that that documented

For peer review only - http://bmjopen.bmj.com/site/about/guidelines.xhtml

information was often provided in the form of minimal, hand-written notes on papers provided for other purposes (e.g. prescription cards). These findings reflect results from other LMIC studies that have evidenced the exchange of poor-quality referral documents.[14 37-39] However, the current study also evidenced patient reports of not being provided with any documented information during referrals. Further, whilst a small number of inpatient HCPs in the current study explained that they called HCPs to discuss a referral case, this appeared to be dependent on how well they knew the patient or HCP. Such findings indicate that there are further inconsistencies in referral communication practices than previously described. Overall, such deficits are unsurprising given that multiple HCPs in both the current and previous outpatient study reported an absence of structured systems and education provided for handover communication. These findings are also in line with the few previous descriptions from India of an absence of training and protocols for handover practices.[15-17]

In addition to similarities found with previous research, the current study has elucidated numerous novel insights regarding handover and healthcare communication during critical points in inpatient care, which were previously unexplored in the study areas of India. Regarding inpatient medical record keeping, a records officer reported that hospital records were not easily accessible and rarely retrieved. Alongside the inconsistent transportation of patient-held records, this lack of available medical information carries notable risks for patient safety. This is because, without key patient background and/or treatment details, critical oversights can be made that result in adverse events.[4 5 7] Additionally, there were notable variations in patient reports of the provision of healthcare management information during hospital admission and discharge; whilst some patients reported being given

For peer review only - http://bmjopen.bmj.com/site/about/guidelines.xhtml

clear self-care instructions, others stated that they sought information from external sources due to the lack of detail provided by hospital HCPs. It appears that the time pressures experienced by HCPs were a significant contributory factor to inconsistencies in HCP to patient communication, particularly at the point of discharge. During interviews, senior doctors reported often being so busy with high patient loads that they would pass the duty of writing discharge notes to interns or nurses. Additionally, it seemed that more time was spent on verbal discharge communication, with a nurse reporting that they typically took approximately twenty minutes per patient to explain discharge instructions. Such practices may be compromising the retention of key healthcare information, as global literature suggests that patients can struggle to absorb verbal details provided during consultations.[40] The potential implications of these are findings are significant, given the associations that have been found between deficient discharge communication and an increased likelihood of adverse events.[7-10]

Other key issues affecting handover and healthcare communication during admission and discharge were mentioned solely by each participant group. For patients, some recalled receiving impolite treatment from hospital doctors during admission. Additionally, a small number of patients were dissatisfied with the take-home nutritional advice provided, as they felt it failed to take into account their socioeconomic deprivation. These results may be explained by the reported lack of communication training in medical education, as well as a historic tendency for paternalistic physician conduct in India.[41] In other areas of India and Asia, research on HCP-patient communication has also evidenced asymmetric power balances and patient dissatisfaction during patient consultations.[42] Such findings reveal the need for more patient-centred communication, particularly for poorer patients that make up

For peer review only - http://bmjopen.bmj.com/site/about/guidelines.xhtml

a significant proportion of public healthcare users. As for HCPs, during interviews many doctors recognised the need for an increase in public healthcare resource provision, as well as structured systems for information exchange. Some also discussed the promise of implementing "e-health" systems, with a doctor in Kerala reporting that public healthcare facilities across the state will be transitioning to computerised systems. Whilst our colleagues from Kerala report that this development is in its early stages, it holds potential as similar systems have advanced the accessibility and quality of healthcare information around the world.[43 44]

4.2 Strengths and limitations

As far as the authors are aware, this is the first study to qualitatively explore both patient and HCP knowledge, attitudes and barriers to multiple areas of handover and healthcare communication during chronic NCD inpatient care in India. The use of qualitative methodology and inclusion of multiple healthcare sites has revealed a number of key issues that are supported among the HIC and emerging LMIC literature, suggesting likely transferability to other settings. Interviews with both patients and HCPs have provided of a variety of valuable perspectives, which has helped to identify critical areas impacting the continuity of chronic NCD inpatient care. The number of interviews conducted helped to achieve data saturation for both participant groups and study credibility was strengthened via the use of multi-analyst triangulation.[34]

The lack of documented inclusion/exclusion rates for participation is a limitation, as this could not be recorded. In addition, the accuracy of recall of the

For peer review only - http://bmjopen.bmj.com/site/about/guidelines.xhtml

minority of patients interviewed at home may have been limited by the delay between recruitment and data collection. Recruitment challenges meant that patient participants were predominantly older (i.e. 45yrs+), which limited exploration of younger patient experiences; this was, however, largely unsurprising given the study exclusively recruited patients with chronic NCDs. The cross-cultural nature of this research may have resulted in constraints during data collection and analysis, as in-group bias could have affected participants' willingness to openly converse with a non-local researcher.[45] Social desirability bias from the use of individual interviews and participant's awareness that the interviewer was a public health professional may have also affected truthfulness of the data.[46] Despite these challenges, the recurrence of themes indicating data saturation and the finding that our results are supported by existing literature suggests that they had minimal impact.

4.3 Conclusions and next steps

This study has found that handover and healthcare communication for chronic NCD inpatients during referrals/transfers, hospital admission and discharge is often fragmented. The critical barriers appear to be an absence of structured information exchange systems and HCP education. There is also a growing recognition of the need for the government to strengthen primary healthcare infrastructure in line with the declaration of Alma Alta.[47] This will greatly assist in increasing accessibility of care and subsequently reducing pressure on hospital services. It will also be required to address the United Nations' sustainable development goals regarding universal health coverage and reducing premature deaths from NCDs.[48] In addition, the implementation of structured documentation, systems and training is urgently

For peer review only - http://bmjopen.bmj.com/site/about/guidelines.xhtml

required to manage critical care transitions such as referral and discharge. Research from both HIC and LMIC settings has proven that such interventions can improve continuity and safety of care.[4 17 37 49] Regarding future steps, during HCP interviews it was reported that public healthcare facilities in Kerala will be transitioning to computerised “e-health” information systems. The Indian government has also since pledged to digitise all public healthcare information systems in the country via an “Integrated Health Information Platform”.[50] Whilst such developments hold promise and are progressing in Kerala, they remain in their initial stages in many states and face numerous infrastructural challenges. Additionally, they are not likely to target issues regarding HCP to patient communication, patient access to healthcare information and information exchange between public and private healthcare providers.

Therefore, a mixed-methods pilot study exploring the design and implementation of patient-held record booklets is suggested. This could ameliorate some of the current issues by incorporating disease-specific and structured documents, which have been shown to improve the recording of clinical information and can provide a means of organising records in a logical and accessible way.[49 51 52] The patient-held nature of this strategy could also increase patient access to key healthcare information, which may improve self-management. Given the unstructured, predominantly paper-based systems utilised across the study sites, this is an area for development that has been welcomed by Indian national and international experts, as well as by patients and HCPs in our study areas. There have also been multiple international successes of improved continuity of care via utilisation of similar patient-held/home-based records in both outpatient and maternal and child healthcare.[26-30 53] In order to maximise booklet utilisation, it would be

For peer review only

necessary to address the issues surrounding patient retention and understanding of the importance of medical documents. Initial key steps could be to involve both patients and HCPs in the design process and accompany the introduction of booklets with relevant promotion, training and incentives.

Finally, given the rising burden of NCDs across LMICs, this research is timely and crucial for effective health systems development. It is vital that further LMIC research is conducted to explore critical factors affecting handover, continuity of care and health systems integration and to develop sustainable and cost-effective interventions.

ACKNOWLEDGEMENTS

This research was supported by the National Institute for Health Research (NIHR) Collaboration for Leadership in Applied Health Research and Care West Midlands (NIHR CLAHRC WM). We would like to extend our thanks to all patients, healthcare staff and researchers who kindly took the time to participate in this project. We are also indebted and give thanks to the participating hospitals and community healthcare facilities. Finally, we are very grateful to the Directors of Health from both Himachal Pradesh and Kerala States, India, for their assistance in facilitating this project. This research would not have been possible without their support.

For peer review only - http://bmjopen.bmj.com/site/about/guidelines.xhtml

AUTHOR CONTRIBUTIONS

In order of the author list: CH: Data curation, formal analysis, visualisation, writing (both original draft and final review and editing). SJ: Investigation, data curation, project administration, writing (review and editing). JP: Conceptualisation, funding acquisition, project administration, supervision, writing (review and editing). SS: Funding acquisition, project administration, supervision, writing (review and editing). SG(oenka): Funding acquisition, projection administration, supervision, writing (review and editing). PD: Conceptualisation, funding acquisition, project administration, supervision, writing (review and editing). PG: Conceptualisation, data curation, funding acquisition, writing (review and editing). SG(reenfield): Funding acquisition, formal analysis, writing (review and editing). RL: Conceptualisation, funding acquisition, writing (review and editing). SMH: Conceptualisation, funding acquisition, investigation, methodology, project administration, supervision, writing (review and editing).

FUNDING STATEMENT

This research was joint-funded by the Department of International Development, the Economic and Social Research Council, the Medical Research Council and the Wellcome Trust; grant number: MR/M00287X/1. The funders had no role in study design, conduct or reporting.

For peer review only

For peer review only - http://bmjopen.bmj.com/site/about/guidelines.xhtml

COMPETING INTERESTS STATEMENT

The authors of this manuscript have no competing interests to disclose.

ETHICS APPROVAL

This study was reviewed and approved by the Centre for Chronic Disease Control Independent Ethics Committee, India, and the Amrita Institute of Medical Sciences Institutional Ethics Committee, India. Data archives will be stored at the University of Birmingham, in accordance with the University's code of practice.

DATA SHARING STATEMENT

De-identified participant transcript data generated and analysed during the current study are available from the corresponding author on reasonable request.

Contact Details: Dr. Semira Manaseki-Holland, s.manasekiholland@bham.ac.uk.

For peer review only

REFERENCES

1. Islam SMS, Purnat TD, Phuong NTA, et al. Non-Communicable Diseases (NCDs) in developing countries: a symposium report. *Globalization and health* 2014;10(1):81.
2. World Health Organisation. Communication during patient hand-overs. *Patient safety solutions* 2007;1(3):1-4.
3. British Medical Association. Safe handover: safe patients. *Guidance on clinical handover for clinicians and managers London: BMA* 2004;7:141.
4. Merten H, van Galen LS, Wagner C. Safe handover. *Bmj* 2017;359:j4328. doi: https://dx.doi.org/10.1136/bmj.j4328
5. Australian Council for Safety Quality in Health Care. Clinical handover and patient safety: Literature review report. 2005
6. Hesselink G, Schoonhoven L, Barach P, et al. Improving patient handovers from hospital to primary care: a systematic review. *Ann Intern Med* 2012;157(6):417-28. doi: https://dx.doi.org/10.7326/0003-4819-157-6-201209180-00006
7. Forster AJ, Murff HJ, Peterson JF, et al. The incidence and severity of adverse events affecting patients after discharge from the hospital. *Ann Intern Med* 2003;138(3):161-7.
8. Moore C, Wisnivesky J, Williams S, et al. Medical errors related to discontinuity of care from an inpatient to an outpatient setting. *J Gen Intern Med* 2003;18(8):646-51.
9. Li JY, Yong TY, Hakendorf P, et al. Timeliness in discharge summary dissemination is associated with patients' clinical outcomes. *Journal of evaluation in clinical practice* 2013;19(1):76-79.
10. van Walraven C, Seth R, Austin PC, et al. Effect of discharge summary availability during post-discharge visits on hospital readmission. *J Gen Intern Med* 2002;17(3):186-92.
11. Bradley S, Mott S. Adopting a patient-centred approach: an investigation into the introduction of bedside handover to three rural hospitals. *J Clin Nurs* 2014;23(13-14):1927-36. doi: https://dx.doi.org/10.1111/jocn.12403
12. Humphries C, Majothi S, Krynicki C, et al. Handover communication in low and middle-income countries: A systematic review PROSPERO international prospective register of systematic reviews2019 [Available from: https://www.crd.york.ac.uk/prospero/display_record.php?RecordID=9309.
13. Dreyer R, Viljoen AJ. Evaluation of factors and patterns influencing the 30-day readmission rate at a tertiary-level hospital in a resource-constrained setting in Cape Town, South Africa. *South African Medical Journal Suid-Afrikaanse Tydskrif Vir Geneeskunde* 2019;109(3):164-68.
14. Azim A, Bhatia P, Baronia A, et al. Deficits in referral notes during inter-hospital transfers of critically ill patients: an observational study from a developing nation. *International Journal of Perioperative Ultrasound & Applied Technologies* 2013;2(2):89.
15. Kumar P, Jithesh V, Vij A, et al. Who is more hands on with hand-offs? A comparative study of clinical handovers among doctors and nurses in a tertiary care center in India. *International Journal of Research Foundation of Hospital & Healthcare Administration* 2015;3(1):33-40.
16. Kumar P, Jithesh V, Vij A, et al. Need for a hands-on approach to hand-offs: A study of nursing handovers in an Indian Neurosciences Center. *Asian journal of neurosurgery* 2016;11(1):54.
17. Rajavelu P, Rajagopalan A. Pre-formatted written discharge summary—a step towards quality assurance in the emergency department. *International journal of emergency medicine* 2008;1(4):321-25.
18. Kumar S. Assessment of quality of handwritten discharge tickets of breast cancer patients: The gaps are wide and need remedy. 2004
19. Saxena M, Srivastava A, Dwivedi P, et al. Is quality of care during childbirth consistent from admission to discharge? A qualitative study of delivery care in Uttar Pradesh, India. *PLoS ONE [Electronic Resource]* 2018;13(9):e0204607.

For peer review only - http://bmjopen.bmj.com/site/about/guidelines.xhtml

20. Humphries C, Jaganathan S, Panniyammakal J, et al. Investigating clinical handover and healthcare communication for outpatients with chronic disease in India: A mixed-methods study. *PLoS ONE [Electronic Resource]* 2018;13(12):e0207511.
21. Marshall MN. Sampling for qualitative research. *Fam Pract* 1996;13(6):522-26.
22. Palys T. Purposive Sampling. In: Given LM, ed. The Sage Encylopedia of Qualitative Research Methods. Los Angeles: Sage 2008:697-98.
23. World Health Organistion. Information for Researchers Concerning Informed Decision Making [Internet]. Available from: http://www.who.int/rpc/research_ethics/Process_seeking_IF_printing2.pdf accessed 01/05/18.
24. Seale C. Quality in qualitative research. *Qualitative inquiry* 1999;5(4):465-78.
25. Baker SE, Edwards R, Doidge M. How many qualitative interviews is enough?: Expert voices and early career reflections on sampling and cases in qualitative research. 2012
26. Shah PM, Selwyn BJ, Shah K, et al. Evaluation of the home-based maternal record: a WHO collaborative study. *Bull World Health Organ* 1993;71(5):535-48.
27. Osaki K, Hattori T, Kosen S. The role of home-based records in the establishment of a continuum of care for mothers, newborns, and children in Indonesia. *Glob Health Action* 2013;6:1-12. doi: https://dx.doi.org/10.3402/gha.v6i0.20429
28. Turner KE, Fuller S. Patient-held maternal and/or child health records: meeting the information needs of patients and healthcare providers in developing countries? *Online journal of public health informatics* 2011;3(2)
29. Takeuchi J, Sakagami Y, Perez RC. The Mother and Child Health handbook in Japan as a health promotion tool: An overview of its history, contents, use, benefits, and global influence. *Global pediatric health* 2016;3:2333794X16649884.
30. Walton S, Bedford H, Dezateux C. Use of personal child health records in the UK: findings from the millennium cohort study. *Bmj* 2006;332(7536):269-70.
31. Spencer L, Ritchie J. Qualitative data analysis for applied policy research. Analyzing qualitative data: Routledge 2002:187-208.
32. Braun V, Clarke V. Using thematic analysis in psychology. *Qualitative research in psychology* 2006;3(2):77-101.
33. Corbin JM, Strauss A. Grounded theory research: Procedures, canons, and evaluative criteria. *Qualitative sociology* 1990;13(1):3-21.
34. Lincoln YS, Guba EG. Naturalistic inquiry. Beverley Hills: ca: sage, 1985.
35. Gale NK, Heath G, Cameron E, et al. Using the framework method for the analysis of qualitative data in multi-disciplinary health research. *BMC medical research methodology* 2013;13(1):117.
36. Das J, Hammer J. Quality of primary care in low-income countries: facts and economics. *Annu Rev Econ* 2014;6(1):525-53.
37. Janati A, Amini A, Adham D, et al. Assessing the quality of referral letters written by general practitioners: a cross-sectional study in rural Iran. *Cadernos de Saude Publica* 2017;33(2):e00043016.
38. Gyedu A, Baah EG, Boakye G, et al. Quality of referrals for elective surgery at a tertiary care hospital in a developing country: an opportunity for improving timely access to and cost-effectiveness of surgical care. *Int J Surg* 2015;15:74-8. doi: https://dx.doi.org/10.1016/j.ijsu.2015.01.033
39. Osinaike BO, Esezobor CI, Akinsola OJ. Quality of Referral Letters to the Paediatric Department of a Tertiary Hospital in Nigeria. *Nigerian Quarterly Journal of Hospital Medicine* 2013;23(4):273-9.
40. Kessels RP. Patients' memory for medical information. *Journal of the Royal Society of Medicine* 2003;96(5):219-22.

For peer review only - http://bmjopen.bmj.com/site/about/guidelines.xhtml

41. Ghooi R, Deshpande S. Patients’ rights in India: an ethical perspective. *Indian J Med Ethics* 2012;9(277):e8.
42. Pun J, Chan EA, Wang S, et al. Health professional-patient communication practices in East Asia: An integrative review of an emerging field of research and practice in Hong Kong, South Korea, Japan, Taiwan, and Mainland China. *Patient education and counseling* 2018
43. Piette JD, Lun KC, Moura LA, Jr., et al. Impacts of e-health on the outcomes of care in low- and middle-income countries: where do we go from here? *Bull World Health Organ* 2012;90(5):365-72. doi: https://dx.doi.org/10.2471/BLT.11.099069
44. Campanella P, Lovato E, Marone C, et al. The impact of electronic health records on healthcare quality: a systematic review and meta-analysis. *Eur J Public Health* 2016;26(1):60-4. doi: https://dx.doi.org/10.1093/eurpub/ckv122
45. Tajfel H. Social psychology of intergroup relations. *Annual review of psychology* 1982;33(1):1-39.
46. Spector P. Social desirability bias. *Encyclopedia of social science research methods* 2004:1045-46.
47. World Health Organization. Declaration of Alma-Ata: International Conference on Primary Health Care, Alma-Ata, USSR, 6–12 September 1978. *Retrieved February* 1978;14:2006.
48. United Nations. Sustainable Development Goals. [Internet]. Available from: https://www.un.org/sustainabledevelopment/sustainable-development-goals/2018.
49. Ramanayake R. Structured printed referral letter (form letter); saves time and improves communication. *Journal of family medicine and primary care* 2013;2(2):145.
50. Government of India - Press Information Bureau. Health Ministry launches a new state-of-the art Information Platform to monitor public health surveillance 2018 [Available from: http://pib.nic.in/PressReleaseIframePage.aspx?PRID=1553941 accessed 01/01/2019 2019.
51. Doyle M-A, Malcolm JC, Liu D, et al. Using a structured discharge letter template to improve communication during the transition from a specialized outpatient diabetes clinic to a primary care physician. *Canadian journal of diabetes* 2015;39(6):457-66.
52. Lilford RJ, Kelly M, Baines A, et al. Effect of using protocols on medical care: randomised trial of three methods of taking an antenatal history. *Bmj* 1992;305(6863):1181-4.
53. Ibrahim H, Munkhbayar U, Toivgoo A, et al. Can universal patient-held health booklets promote continuity of care and patient-centred care in low-resource countries? The case of Mongolia. *BMJ Qual Saf* 2019:bmjqs-2018-008941.

For peer review only

For peer review only - http://bmjopen.bmj.com/site/about/guidelines.xhtml

Figure 1. Similarities and differences between the content of patient & HCP data with related subcategories

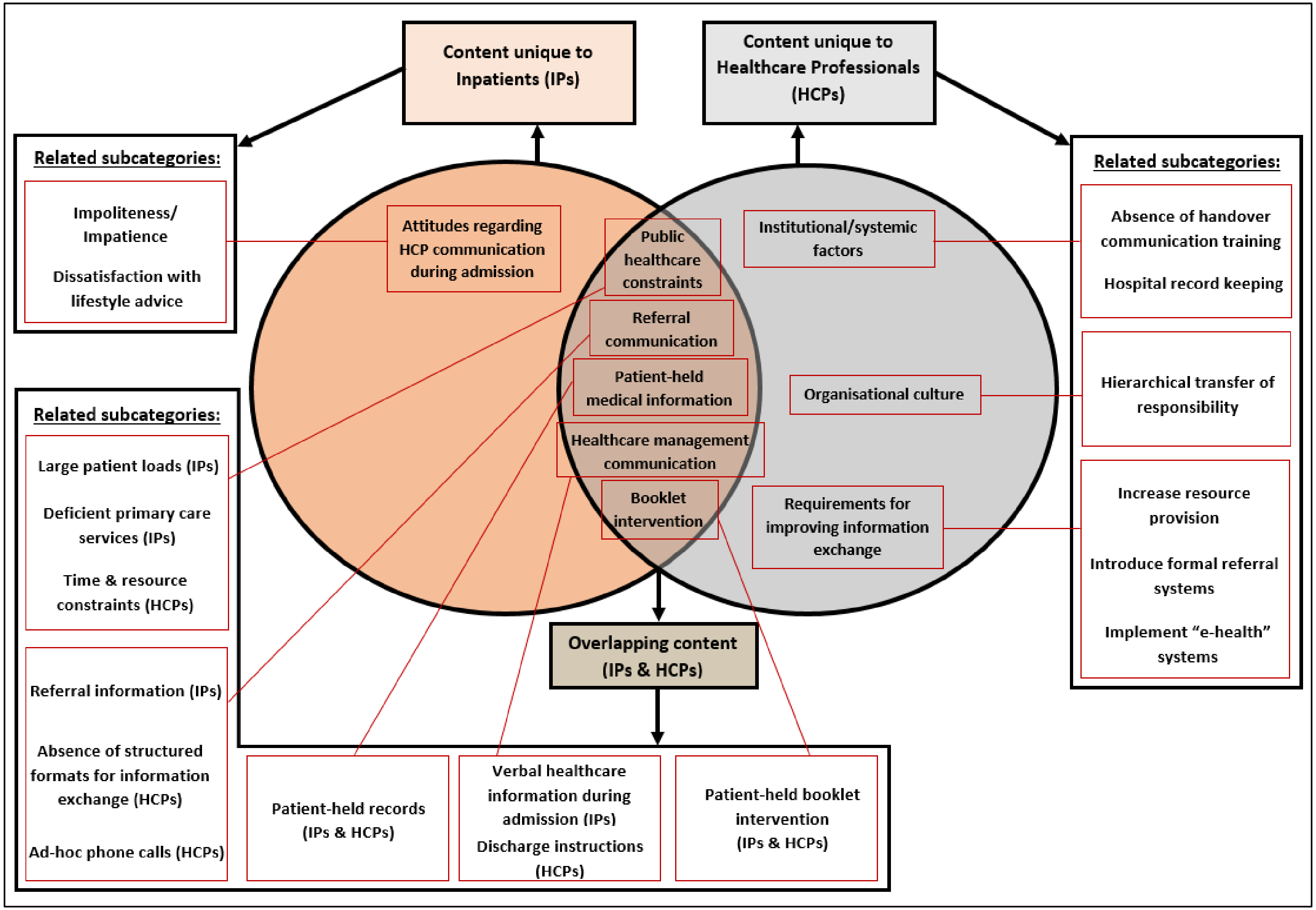

S1. Additional information regarding the Indian healthcare system

1. National context: structure of public healthcare system

The basic structure of the public healthcare system in India is as follows:[1]

- National level: Ministry of Health and Family Welfare.
- State-level: State Department of Health and Family welfare in each state.
- Regional level: covers 3 - 5 districts. Headed by State Directorate of Health.
- District level: Middle-level management organisation serving as a link between the regional and state structures and the peripheral and PHC structures.
- Sub-divisional/Taluk level: Hospitals/hospitals with specialty care (Taluk headquarters hospitals). Healthcare services are rendered via the office of Assistant District Health and Family Welfare Officer.
- Community level: CHCs that cater for 80,000-120,000 population and PHCs that cover approximately 20,000 – 30,000 population.

For peer review only

2. Public healthcare in India

The quality of public healthcare across India varies notably between states and urban and rural areas. Whilst there are some examples of excellence in a select few states, generally the public sector is generally failing to meet the basic healthcare needs of the growing population. Services being too far away, a lack of trained personnel, deficient medical supplies and limited facility opening times are some of the principal reasons for current shortfalls.[2] Regarding primary health

centres, government estimates have indicated that up to 10% are without a doctor, 37% are without a laboratory technician and 25% are without a pharmacist.[3] These issues appear to be particularly prevalent within socioeconomically vulnerable areas, where facilities have been reported to lack essential medical supplies and are closed more than 50% of the time. In addition, public healthcare facilities are often the sole source of qualified healthcare professionals in rural areas, which is where many of the most socioeconomically vulnerable live.[2]

3. Private healthcare in India

Due to a lack of prioritisation from economic planners, public healthcare in India has lacked funding over multiple decades. Therefore, the increasing prevalence of chronic diseases, as well as ongoing challenges of infectious diseases, has placed an enormous strain on public health systems that cannot be feasibly managed. Subsequently, the private healthcare sector has proliferated to meet the rising needs, expectations and earnings of the growing population. So much so in fact, that surveys now show that private providers dominate healthcare service provision in India.[2]

At the top end of the market, the private healthcare sector has grown extensively and now has world-class facilities. Private hospital care has become an export sector for medical tourism that cares for around 200,000 international patients each year.[4] However, private healthcare providers lack regulation and quality remains inconsistent across facilities. In many cases private facilities deliver services without appropriate equipment or expertise and, although it is preferential for many to visit private providers, high out-of-pocket costs are often incurred. It has been reported that more than 40% of private hospital patients have to sell assets or

For peer review only

borrow money to fund their care.[5] This means that many patients of lower socioeconomic status remain unable to access such care, while others fall further into poverty as a result of expenses.

REFERENCES

1. Care Continuum Alliance, India. India – Health Care System Structure and Organization. 2009. Available from: http://dmaa.pbworks.com/w/page/17960772/India%20-%20Health%20Care%20System%20Structure%20and%20Organization

2. Joumard I, Kumar A. Improving health outcomes and health care in India. Paris: Organisation for Economic Cooperation and Development (OECD) Working Papers, No. 1184, OECD Publishing. 2015. Available from: http://www.oecd-ilibrary.org/economics/improving-health-outcomes-and-health-care-in-india_5js7t9ptcr26-en

3. Ministry of Health and Family Welfare, Government of India. Rural Health Statistics in India. 2012. Available from: https://nrhm.gujarat.gov.in/images/pdf/publications/RHS_2012.pdf

4. Lunt N, Smith R, Exworthy M, Green S, Horsfall D, Mannion R. Medical Tourism: Treatments, Markets and Health System Implications: a Scoping Review. OECD Publishing. 2010. Available from: Paris http://www.oecd.org/els/health-systems/48723982.pdf

5. The World Bank - Health, Nutrition, Population Sector Unit India South Asian Region. Raising the sights: better health systems for India's poor. Available from: https://openknowledge.worldbank.org/handle/10986/14080

For peer review only

S2. Additional information regarding the study settings

1. Himachal Pradesh

Himachal Pradesh is principally a rural state in northern India. It has a population of 6.86 million people and the average literacy rate is 83.3%, which is higher than the national average (74%). However, rates remain notably lower for women compared to men (76.6% vs. 90.8%, respectively).[6] Private HCPs are less prevalent in Himachal Pradesh compared to many states and utilisation of public healthcare remains relatively high.[7] A recent study found that the availability of public health services in the state was deemed adequate as compared to standards of other hill states, but with an unequal distribution of resources across regions.[8]

2. Kerala

Kerala state is in the south-west of India. It has a population of 34.8 million people and a greater than national average urban-based population of 47.7%. It has the highest overall literacy rate of all the states in India (93.9%; men 96.1%, women 92.1).[9] There are a relatively large number of government healthcare facilities in Kerala but the healthcare environment has become increasingly complex due to the growing popularity and presence of private healthcare facilities, which are predominantly situated in urban settings. Despite this, public healthcare facilities in Kerala remain the first point of care for many patients and continue to deliver essential services.[10]

For peer review only

REFERENCES

6. Government of India. Himachal Pradesh Population Census Data 2011 [Internet]. Available from: https://www.census2011.co.in/census/state/himachal+pradesh.html accessed 13/06/18 2017.

7. Government of India. Himachal Pradesh Development Report. New Delhi: Academic Foundation, 2005.

8. Suman.; Bhutani S. Availability of health services in Himachal Pradesh *International Journal of Advanced Research and Development* 2017;2(6):231-38.

9. Government of Kerala. Kerala Population Census Data 2011 [Internet]. Available from: https://www.census2011.co.in/census/state/kerala.html accessed 13/06/18.

10. Government of Kerala. Kerala Development Report. New Delhi: Academic Foundation, 2008.

For peer review only

Reporting checklist for qualitative study.

Based on the SRQR guidelines.

Instructions to authors

Complete this checklist by entering the page numbers from your manuscript where readers will find each of the items listed below.

Your article may not currently address all the items on the checklist. Please modify your text to include the missing information. If you are certain that an item does not apply, please write "n/a" and provide a short explanation.

Upload your completed checklist as an extra file when you submit to a journal.

In your methods section, say that you used the SRQR reporting guidelines, and cite them as:

O'Brien BC, Harris IB, Beckman TJ, Reed DA, Cook DA. Standards for reporting qualitative research: a synthesis of recommendations. Acad Med. 2014;89(9):1245-1251.

		Reporting Item	Page Number
	#1	Concise description of the nature and topic of the study identifying the study as qualitative or indicating the approach (e.g. ethnography, grounded theory) or data collection methods (e.g. interview, focus group) is recommended	1
	#2	Summary of the key elements of the study using the abstract format of the intended publication; typically	2-3

For peer review only

		includes background, purpose, methods, results and conclusions	
Problem formulation	#3	Description and signifcance of the problem / phenomenon studied: review of relevant theory and empirical work; problem statement	5-7
Purpose or research question	#4	Purpose of the study and specific objectives or questions	6-7
Qualitative approach and research paradigm	#5	Qualitative approach (e.g. ethnography, grounded theory, case study, phenomenolgy, narrative research) and guiding theory if appropriate; identifying the research paradigm (e.g. postpositivist, constructivist / interpretivist) is also recommended; rationale. The rationale should briefly discuss the justification for choosing that theory, approach, method or technique rather than other options available; the assumptions and limitations implicit in those choices and how those choices influence study conclusions and transferability. As appropriate the rationale for several items might be discussed together.	6-12
Researcher characteristics and reflexivity	#6	Researchers' characteristics that may influence the research, including personal attributes, qualifications / experience, relationship with participants, assumptions and / or presuppositions; potential or actual interaction between researchers' characteristics and the research	6-12

For peer review only

		questions, approach, methods, results and / or transferability	
Context	#7	Setting / site and salient contextual factors; rationale	7
Sampling strategy	#8	How and why research participants, documents, or events were selected; criteria for deciding when no further sampling was necessary (e.g. sampling saturation); rationale	7
Ethical issues pertaining to human subjects	#9	Documentation of approval by an appropriate ethics review board and participant consent, or explanation for lack thereof; other confidentiality and data security issues	37
Data collection methods	#10	Types of data collected; details of data collection procedures including (as appropriate) start and stop dates of data collection and analysis, iterative process, triangulation of sources / methods, and modification of procedures in response to evolving study findings; rationale	6-12
Data collection instruments and technologies	#11	Description of instruments (e.g. interview guides, questionnaires) and devices (e.g. audio recorders) used for data collection; if / how the instruments(s) changed over the course of the study	6-12
Units of study	#12	Number and relevant characteristics of participants, documents, or events included in the study; level of participation (could be reported in results)	14-17

For peer review only

Data processing	#13	Methods for processing data prior to and during analysis, including transcription, data entry, data management and security, verification of data integrity, data coding, and anonymisation / deidentification of excerpts	12-13
Data analysis	#14	Process by which inferences, themes, etc. were identified and developed, including the researchers involved in data analysis; usually references a specific paradigm or approach; rationale	12-13
Techniques to enhance trustworthiness	#15	Techniques to enhance trustworthiness and credibility of data analysis (e.g. member checking, audit trail, triangulation); rationale	12-13
Syntheses and interpretation	#16	Main findings (e.g. interpretations, inferences, and themes); might include development of a theory or model, or integration with prior research or theory	14-28
Links to empirical data	#17	Evidence (e.g. quotes, field notes, text excerpts, photographs) to substantiate analytic findings	21-28
Intergration with prior work, implications, transferability and contribution(s) to the field	#18	Short summary of main findings; explanation of how findings and conclusions connect to, support, elaborate on, or challenge conclusions of earlier scholarship; discussion of scope of application / generalizability; identification of unique contributions(s) to scholarship in a discipline or field	29-32

For peer review only

Limitations	#19	Trustworthiness and limitations of findings	33
Conflicts of interest	#20	Potential sources of influence of perceived influence on study conduct and conclusions; how these were managed	37
Funding	#21	Sources of funding and other support; role of funders in data collection, interpretation and reporting	37

The SRQR checklist is distributed with permission of Wolters Kluwer © 2014 by the Association of American Medical Colleges. This checklist was completed on 26. November 2018 using http://www.goodreports.org/, a tool made by the EQUATOR Network in collaboration with Penelope.ai

For peer review only
